# Gut virome profiling identifies a widespread bacteriophage family associated with metabolic syndrome

**DOI:** 10.1038/s41467-022-31390-5

**Published:** 2022-06-23

**Authors:** Patrick A. de Jonge, Koen Wortelboer, Torsten P. M. Scheithauer, Bert-Jan H. van den Born, Aeilko H. Zwinderman, Franklin L. Nobrega, Bas E. Dutilh, Max Nieuwdorp, Hilde Herrema

**Affiliations:** 1 Departments of Internal and Experimental Vascular Medicine, Amsterdam University Medical Centers, Location AMC, Amsterdam, the Netherlands; 2 Amsterdam Gastroenterology Endocrinology Metabolism, Endocrinology, metabolism and nutrition, Amsterdam, the Netherlands; 3 Amsterdam Cardiovascular Sciences, Diabetes & Metabolism, Amsterdam, the Netherlands; 4 Department of Clinical Epidemiology, Biostatistics and Bioinformatics, Amsterdam University Medical Centers, Location AMC, University of Amsterdam, Amsterdam, the Netherlands; 5 School of Biological Sciences, Faculty of Environmental and Life Sciences, University of Southampton, Southampton, UK; 6 Theoretical Biology and Bioinformatics, Science for Life, Utrecht University, Utrecht, the Netherlands; 7 Institute of Biodiversity, Faculty of Biological Sciences, Cluster of Excellence Balance of the Microverse, Friedrich-Schiller-University Jena, Jena, Germany

**Keywords:** Bacteriophages, Microbiome, Phage biology, Obesity

## Abstract

There is significant interest in altering the course of cardiometabolic disease development via gut microbiomes. Nevertheless, the highly abundant phage members of the complex gut ecosystem -which impact gut bacteria- remain understudied. Here, we show gut virome changes associated with metabolic syndrome (MetS), a highly prevalent clinical condition preceding cardiometabolic disease, in 196 participants by combined sequencing of bulk whole genome and virus like particle communities. MetS gut viromes exhibit decreased richness and diversity. They are enriched in phages infecting *Streptococcaceae* and *Bacteroidaceae* and depleted in those infecting *Bifidobacteriaceae*. Differential abundance analysis identifies eighteen viral clusters (VCs) as significantly associated with either MetS or healthy viromes. Among these are a MetS-associated *Roseburia* VC that is related to healthy control-associated *Faecalibacterium* and *Oscillibacter* VCs. Further analysis of these VCs revealed the *Candidatus Heliusviridae*, a highly widespread gut phage lineage found in 90+% of participants. The identification of the temperate *Ca. Heliusviridae* provides a starting point to studies of phage effects on gut bacteria and the role that this plays in MetS.

## Abstract (web summary)

Here, the authors characterize gut viromes in a cohort of individuals with metabolic syndrome, which they find associated with a highly widespread family of gut bacteriophages they name *Candidatus Heliusviridae*.

## Introduction

The human gut microbiome influences many (metabolic) processes, including digestion, the immune system^1^, and endocrine functions^2^. It is also involved in diseases such as type 2 diabetes^3^, fatty liver disease^4^ and inflammatory bowel disease^5^. Though studies of these gut microbiome effects on health and disease mostly focus on bacteria, increasing attention is devoted to bacteriophages (or phages).

Phages are viruses that infect bacteria. By infecting bacteria, they can significantly alter gut bacterial communities, mainly by integrating into bacterial genomes as prophages (lysogeny) or killing bacteria (lysis). Such alterations to bacterial communities in turn affect the interactions between bacteria and host, making phages part of an interactive network with bacteria and hosts. For example, an increase in phage lytic action is linked to decreased bacterial diversity in inflammatory bowel disease^6,7^, prophage integration into *Bacteroides vulgatus* modifies bacterial bile acid metabolism^8^, and dietary fructose intake prompts prophages to lyse their bacterial hosts^9^.

Gut virome alterations have been linked to several disease states like inflammatory bowel diseases^6,7^, malnutrition^10^, and type 2 diabetes^11^. But many such studies have not been able to identify specific viral lineages that are involved in such diseases, mainly due to the lack of viral marker genes^12,13^ and high phage diversity due to their rapid evolution^14^. Consequently, human gut phage studies are limited to relatively low taxonomic levels. While recent efforts uncovered viral families that are widespread in human populations, such as the *Crassvirales* phages^15,16^, these have not been successfully linked to disease states. In order to develop microbiome-targeted interventions to benefit human health, it is pivotal to study such higher-level phage taxonomies in the gut among relevant cohorts.

Here, we report on gut virome alterations in metabolic syndrome (MetS) among 196 people. MetS is a collection of clinical manifestations that affects about a quarter of the world population, and is a major global health concern because it can progress into cardiometabolic diseases like type 2 diabetes, cardiovascular disease, and non-alcoholic fatty liver disease^17–19^. As gut bacteria are increasingly seen as contributing agents of MetS^20–22^, it stands to reason that the phages which infect these bacteria exhibit altered population compositions in MetS. Whereas recent research compared gut viromes in relation to MetS^23^, this study was limited to 28 children, in which MetS manifests markedly less well defined than in adults^24^. For our analysis, we focused on dsDNA phages, which form a large majority of gut phages in particular and gut viruses in general^14,25^.

Here, we detail differences in the gut virome in MetS versus healthy controls. We find MetS-connected decreases in virome richness and diversity, which are correlated to bacterial population patterns. We further find that MetS viromes are characterized by high levels of *Streptococcaceae* and *Bacteroidaceae* phages, while *Bifidobacteriaceae* phages were less abundant. Finally, among viral clusters (VC) that are differentially abundant in either MetS or controls, we identify four with significant interrelatedness. These phages are part of a previously undescribed family, which we dub the *Candidatus Heliusviridae*, and which is highly widespread in this and several validation cohorts.

## Results

### Metagenomic sequencing identifies high divergence in MetS viromes

To study gut phage populations, we performed metagenomic sequence analyses on fecal samples of subjects from the Healthy Life in an Urban Setting (HELIUS) cohort^26^, a large population study in Amsterdam, the Netherlands. Because gut phages largely exist in two forms: intracellularly (e.g., integrated into bacterial genomes as prophages) and as free-floating particles, we performed sequencing on two types of sample preparations (Supplementary Fig. 1). Firstly, for 97 MetS and 99 healthy participants we performed bulk whole genome shotgun (WGS) sequencing, which tends to bias in favor of intracellular phages. Secondly, for a subset of 48 participants (24 each of controls and MetS), we made filtrations of free-floating phage particles and sequenced viral-like particle (VLP) metagenomes. Among the MetS participants, central obesity and high blood pressure were nearly universal, being found in 94/97 participants and 91/97, respectively. For further details on the participants of the present study, see the Methods and Supplementary Table 1. Bulk sequencing yielded an average of 23 ± 3.4 million read pairs per sample (median: 22.6 million read pairs), while VLP sequencing yielded 16.5 ± 2.5 million read pairs (median: 16.3 million). Per sample read assemblies and viral sequence prediction resulted in a database of 45,421 unique phage contigs (non-redundant at 90% average nucleotide identity). We grouped these phage contigs by shared protein content^27^ into 6,635 viral clusters (VCs). These comprised 30,161 contigs, while the remainder were singletons that were too distinct to confidently cluster with other phage contigs. Treating such singletons as VCs with one member gave a final dataset of 21,895 VCs.

For further analysis, we mapped quality-controlled reads to viral contigs, and constructed a per-VC RPKM table, which we converted to relative abundances where between-sample comparisons were needed (Supplementary Fig. 1). Analysis of relative abundances per VC across the 196 WGS samples (Supplementary Data 1) showed an high inter-individual diversity in bulk gut viromes, as 19,970 VCs (97.4% of the 20,501 VCs present in WGS samples) were either specific to a single individual or present in fewer than 20/196 (i.e., <10%) of the participants. Only 59 VCs (0.3%), meanwhile, were putative members of the core human gut virome^28^, being present in over 30% of participants (Supplementary Fig. 2a). We notably found two VCs that were found in the bulk virome of over 30% of controls and none of the MetS participants, but none vice versa. In both cases, the viral contigs contained in the VCs were genome fragments (i.e., checkv^29^ completeness of <25%, Supplementary Data 5). The general prevalence pattern was mirrored among the 48 VLP samples, where 9,147 VCs (93.3% of the 9,800 VCs present in VLP samples) were present in less than 10% of the participants, while 61 (0.6%) were present in over 30% of participants (Supplementary Fig. 2b). Interestingly, VCs observed in fewer than 10% of the participants had much higher mean relative abundance among bulk than VLP viromes (WGS: mean 70.1 ± 10.2%, median: 71.8%, VLP: mean 42.1 ± 18.4%, median: 42.6%, Supplementary Fig. 2c, d). Much of the interpersonal gut phage diversity is thus contained in the bulk virome.

### Gut phage and bacterial populations show altered richness and diversity measures in MetS

To gain a deeper understanding of MetS virome community dynamics, we first examined total read fractions that mapped to VCs. In the bulk phage samples the fraction of reads mapping to VCs was significantly lower in MetS compared to controls (Wilcoxon signed-rank test, *p* = 0.023, Supplementary Fig. 3a). This was not caused by differential sequencing depth between the participant groups, as this did not significantly differ between the groups (Wilcoxon signed-rank test, *p* = 0.23). It could instead derive from higher bulk phage micro-diversity causing more fragmented assemblies, thereby decreasing the number of recognized phage sequences. To test this, we constructed cumulative VC ranked-abundance curves of bulk phage samples. These showed that fewer VCs represented the full relative abundance of bulk viromes in MetS than in controls, therefore indicating lower micro-diversity in MetS (Supplementary Fig. 3b). Our findings thus imply that MetS is characterized by lower intracellular phage-to-bacteria ratios, for example through decreased lysogeny rates. For VLP phage populations, we observed the opposite: higher fractions of viral reads among MetS (Wilcoxon signed-rank test, *p* = 0.011, Supplementary Fig. 3c), while sequencing depth again did not significantly differ (Wilcoxon signed-rank test, *p* = 0.65). But because VLP virome cumulative VC ranked-abundance curves showed the same pattern as those of the bulk viromes, thereby indicating decreased micro-diversity in MetS samples, the increase in viral-mapped read fractions for MetS may reflect less fragmented assemblies of these samples (Supplementary Fig. 3d). Thus, while our results suggest decreased lysogeny rates in MetS, we could not definitively determine whether these are paired with increased lytic rates.

**Fig. 1:**
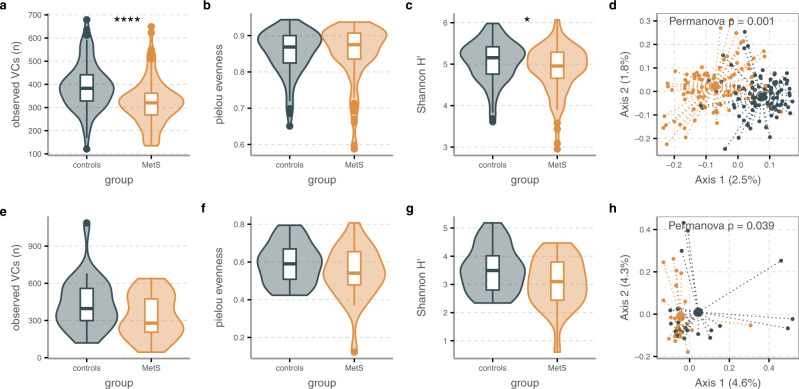
Gut phage populations are altered in MetS. **a–d** Bulk phage populations measured in WGS samples (*n* = 97/*n* = 99 biologically independent samples for MetS and controls, respectively), showing: **a** MetS-associated decreased species richness is evidenced by the number of unique VCs observed per sample, *p* = 7.1 × 10^−^^7^. **b** No change in Pielou evenness measurements, *p* = 0.49. **c** Significantly decreased α-diversity measured by Shannon diversity *p* = 0.02. **d** Clear separation between populations of MetS (orange) and control (blue) participants as shown by β-diversity depicted in a principal coordinates analysis (PCoA) of Bray–Curtis dissimilarities. **e**–**h** VLP phage populations measured in VLP samples (*n* = 24 biologically independent samples for both MetS and controls), showing no significant difference in **e** richness (*p* = 0.11), **f** evenness (*p* = 0.26), and **g** α-diversity (*p* = 0.089), but **h** significantly different populations between MetS (orange) and controls (blue) evidenced by β-diversity. For bulk viromes, Permanova test was adjusted for smoking, age, sex, alcohol use, and metformin use, while analysis of VLP phage populations involved balanced populations that did not need these adjustments. Statistical significance in **a**–**c** and **e**–**g** is according to the two-sided Wilcoxon signed-rank test, where *p* values are denoted as follows: * ≤ 0.05, ** ≤ 0.01, *** ≤ 0.001, **** ≤ 0.0001. The absence of significance level means *p* values were above 0.05. Box plots show the median (middle line), 25th, and 75th percentile (box), with the 25th percentile minus and the 75th percentile plus 1.5 times the interquartile range (whiskers), and outliers (single points). Source data are provided as a Source Data file.

For further analysis of phage communities, we examined virome richness and diversity. We determined phage richness by measuring the number of VCs that were present (i.e., had a relative abundance above 0) in each participant, using a horizontal coverage cutoff of 75%^30^. This showed that besides lowered phage-to-bacteria ratios, bulk phage populations in MetS also had lower VC richness than controls, but equal evenness (Wilcoxon signed-rank test, richness *p* = 7.1 × 10^−^^7^, Pielou evenness *p* = 0.49, Fig. 1a and b). Nevertheless, due to the strong differences in richness, bulk phage α-diversity was significantly decreased among MetS participants (Shannon H′ *p* = 0.02, Fig. 1c). This suggested that MetS bulk gut phage populations are distinct from healthy communities. These results were independent of sequencing depth, as significance levels in richness, evenness, and diversity were unchanged upon calculations with the median of 1000 random data sub-samplings. Indeed, the differences between the two participant groups were underscored by our observation of significant separation between controls and MetS when assessed by principal covariate analyses (PCoA) of β-diversity based on Bray-Curtis dissimilarities (Permanova *p* = 0.001, Fig. 1d). Similar analyses less notably differed among the VLP phage populations, where richness, evenness, and α-diversity were all non-significantly higher in controls (Wilcoxon signed-rank test, richness *p* = 0.11, evenness *p* = 0.26, and α-diversity *p* = 0.089, Fig. 1e–g), though β-diversity still displayed significant separation between the two groups (Permanova *p* = 0.038, Fig. 1h). As both richness and α-diversity were highly positively correlated between the VLP and WGS datasets among the subset of 48 participants (richness: Spearman *ρ* = 0.68, *p* = 1.1 × 10^−^^7^, α-diversity: *ρ* = 0.5, *p* = 3.6 × 10^−4^), we hypothesize that the lack of significance between controls and MetS VLP datasets was driven by the smaller sample size of the VLP dataset.

**Fig. 2:**
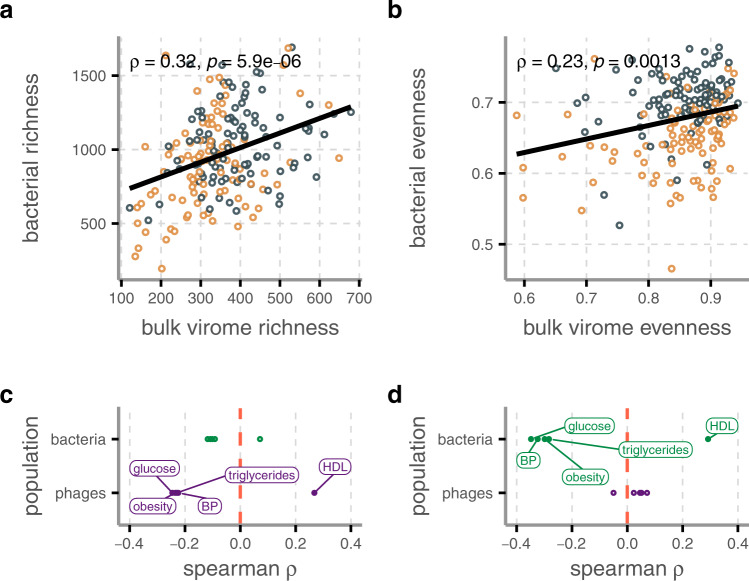
Correlations between phage and bacterial populations as well as between population measures and MetS clinical parameters. Strong correlations between **a** phage richness (observed VCs) and bacterial richness (Chao1 index), as well as between **b** phage and bacterial evenness (Pielou’s index), both with significant positive two-sided Spearman’s rank correlation coefficient. Colors refer to participant groups: MetS (orange) and controls (blue). Both of these measures were correlated to MetS clinical parameters. Plotted are the Spearman’s rank correlation coefficients between the five MetS risk factors and **c** richness and **d** evenness. Points with *q* values below 0.05 are colored in and labeled. *Q* values were obtained after adjusting *p* values for multiple testing with the Benjamini–Hochberg procedure. Source data are provided as a Source Data file.

Because phages are obligate parasites of bacteria, we also studied bacterial community using 16s rRNA amplicon sequencing data. We opted to analyze 16s rRNA amplicon sequencing data over analysis of the metagenomic samples for its greater taxonomic resolution. Bacterial gut populations are often found to be less diverse in obesity-related illnesses such as MetS^31^. Our data underscored this, and showed that MetS bulk viromes mirror bacterial communities in species richness and α-diversity, but not evenness, which was significantly lowered in MetS bacterial populations (Wilcoxon signed-rank test, Chao1 richness *p* = 9.1 × 10^−4^, Shannon *H*′ *p* = 1.5 × 10^−15^, Pielou evenness *p* = 1.8 ×10^−14^, Supplementary Fig. 4a–c). Additionally, bacterial communities separated in PCoA analysis in similar fashion to viromes (Permanova *p* = 0.001, Supplementary Fig. 4d). These results were replicable with data derived from taxonomic profiling of the bulk sequences. Population-level bulk virome changes in MetS are thus directly related to a depletion of host bacteria populations, an assertion strengthened by significant direct correlations between bulk phage and bacterial communities in richness (Spearman *ρ* = 0.42, *p* = 1.3 ×10^−9^, Fig. 2a), evenness (Spearman *ρ* = 0.24, *p* = 5.7 × 10^−4^, Fig. 2b). Though for the subset of 48 samples with VLP data no such correlations were detected, this could have been due to the smaller sample size.

Finally, we studied the relationship between both bulk phages and bacteria on the one hand and the five clinical parameters that constitute MetS on the other. As the bacterial and bulk phage populations did not equally decrease in richness and evenness, they also did not equally correlate with MetS clinical parameters. Rather, bulk phage richness was significantly negatively correlated with obesity, blood glucose levels, blood pressure, and triglyceride concentrations but bacterial richness was not (*q* < 0.05, Fig. 2c and Supplementary Fig. 5). Bacterial evenness, meanwhile, did significantly negatively correlate with these clinical parameters while bulk phage evenness did not (*q* < 0.05, Fig. 2d and Supplementary Fig. 5). Increasingly severe MetS phenotypes thus result in stronger decreases in bacterial evenness than richness, while bulk phage populations exhibit stronger decreases in richness than evenness. The decreasing bacterial evenness could be caused by depletion of certain bacterial species in MetS, which results in the bulk phages infecting these depleted bacteria to become undetectable, thereby decreasing richness more than evenness. Otherwise, the success of certain bacterial species could also decrease evenness. In the process this could conceal rare phage species, which could cause the decreased bulk phage richness. Combined with the results showing MetS-associated reduction in total bulk phage abundance and richness, but not those of VLP populations (Supplementary Fig. 3), our findings indicate that certain phages are either completely absent from the gut or are too rare to detect in MetS.

### Phages infecting select bacterial families are more abundant in MetS viromes

We next studied individual bacterial lineages and the phages that infect them. To do this, we linked viral contigs to bacterial hosts by determining CRISPR protospacer alignments, taxonomies of prophage-containing bacterial sequences, and hosts of previously isolated phages co-clustered in VCs (see methods for details). We found 50,322 host predictions between 7463 VCs (34.1% of all VCs) and 12 bacterial phyla, most commonly *Firmicutes* (5301 VCs) and *Bacteroidetes* (1284 VCs, Supplementary Data 2). We also identified 164 VCs with multi-phyla host range predictions, similar to previous works^32^.

**Fig. 3:**
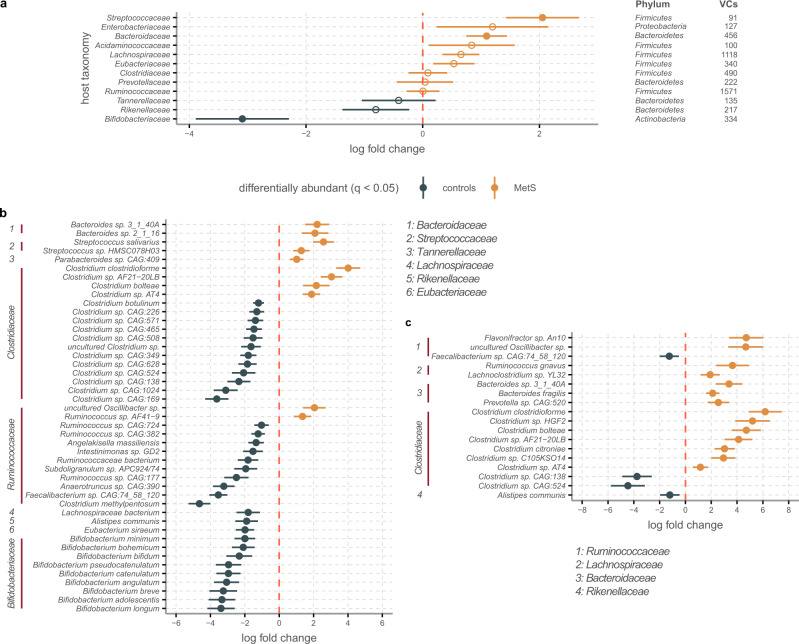
Phages infecting selected bacterial families are differentially abundant in MetS or healthy controls. **a** ANCOM-BC^33^ analysis of bulk phages that infect the 12 bacterial families to which the most VCs were linked shows significant association between *Bifidobacteriaceae* VCs and controls, as well as between *Streptococcaceae* and *Bacteroidaceae* VCs and MetS. Closed circles denote significance, open circles lack of significance. **b** ANCOM-BC of bulk phages infecting the families depicted in **a** and with host predictions at the species level. **c** Same as **b** for VLP phages. For **a** and **b**, *n* = 97/*n* = 99 biologically independent samples for MetS and controls, respectively. For **c**, *n* = 24 biologically independent samples for both MetS and controls. Points show the log fold change as given by ANCOM-BC, error bars denote the standard error adjusted by the Benjamini–Hochberg procedure for multiple testing. In **b** and **c** only, significant species are shown (*q* < 0.05) for brevity. Source data are provided as a Source Data file.

To increase statistical accuracy, we selected the predictions between the 12 most commonly occurring host families and 5188 VCs that were present in bulk viromes (23.7% of VCs). We then performed an analysis of compositions of microbiomes with bias correction (ANCOM-BC)^33^ on the bulk phage population datasets. This showed higher relative abundances in controls for *Bifidobacteriaceae* (*q* = 0.004), and in MetS for *Bacteroidaceae* (*q* = 0.004), and *Streptococcaceae* (*q* = 0.004, Fig. 3a). A complementary analysis of the same 12 families based on 16s rRNA amplicon data showed similar differentially abundance patterns for all three families (Supplementary Fig. 6). Notably, the *Ruminococcaceae* and *Clostridiaceae* bacteria were significantly more abundant in controls, while their bulk phages slightly trended toward MetS. This likely indicates that the various species within these families are unevenly predated upon by phages.

We next performed ANCOM-BC on a subset of 2440 VCs that infected within the most abundant host families and for which host predictions were resolved to the species level (Fig. 3b). This showed that MetS bulk viromes were dominated by phages infecting *Ruminococcaceae, Clostridiaceae, Bacteroidaceae*, and *Streptococcaceae*. Phages infecting species belonging to the former two families were also differentially abundant among controls, together with those infecting *Bifidobacteriaceae* species. Due to difficulties in taxonomic assignments across metagenomic and 16s rRNA amplicon datasets, we were unable to ascertain whether these specific host species were also differentially abundant in bacteriomes. However, the species found as significantly differentially abundant hosts in MetS and control bulk viromes largely conformed with previous findings linking these bacteria to either MetS and related diseases or healthy gut microbiomes^34^. Among free-floating viromes, the top 12 most common host families were the same as in the bulk populations, though no host family was differentially abundant in free-floating populations. At the host species level, differential abundance patterns lined up remarkably well to those in the bulk viromes, reflecting how both phage populations mirror each other (Fig. 3c).

The findings that *Bacteroidaceae* phages were more abundant in MetS led us to analyze abundance of the widespread *Crassvirales* gut phage order, members of which infect in this family^35,36^. Notably, while *Crassvirales* phage relative abundance did not significantly differ between MetS and controls in either free-floating or bulk phage populations, they were significantly more prevalent in control bulk viromes (prevalence controls: 78/99 participants, MetS: 58/97, Fisher’s exact test, *p* = 0.005). This apparent depletion of *Crassvirales* phages in MetS bulk viromes may indicate a decrease in their infectiousness, and is to our knowledge the first link observed between this prominent human gut phage order and a disease state. Alterations to *Crassvirales* phage composition may thus occur at an individual level.

### *Bacteroidaceae* VCs are markers of the MetS virome

**Fig. 4:**
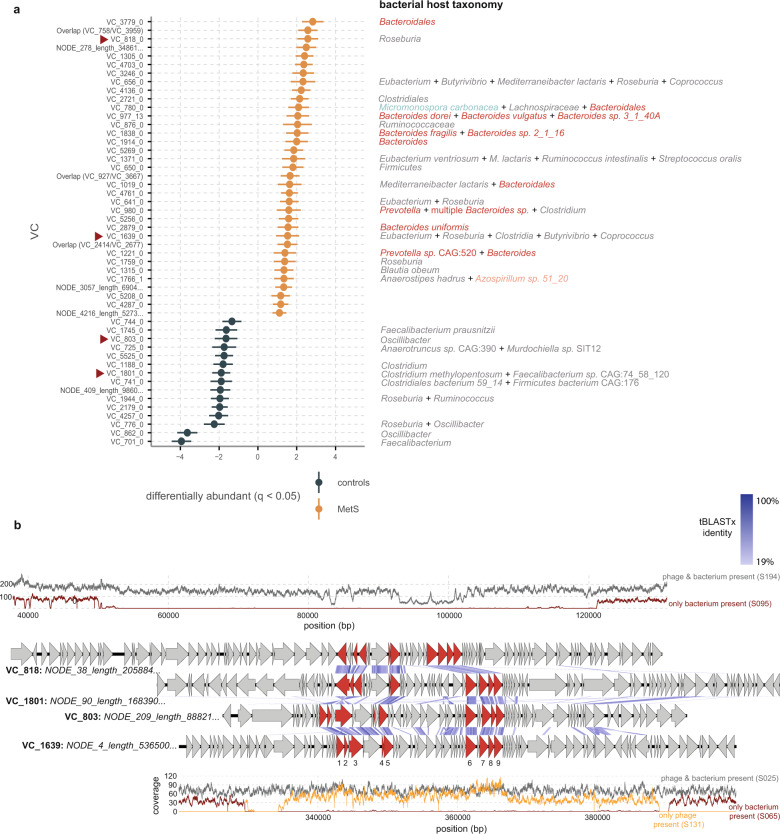
Among significantly differentially abundant VCs some are related. **a** VCs identified by ANCOM-BC as significantly abundant (*q* ≤ 0.05 after implementing the Benjamini–Hochberg procedure for multiple testing). Points show the log fold change as given by ANCOM-BC, error bars denote the standard error adjusted by the Benjamini–Hochberg procedure for multiple testing. The analysis was adjusted for smoking, age, sex, alcohol use, and metformin use. Red arrows mark related VCs further depicted in b. Taxonomic names to the right of the plot denote host predictions, which are colored as follows: *Firmicutes;* gray, *Bacteroidetes;* red, *Actinobacteria*; green, *Proteobacteria*; pink. The full taxonomies are listed in Supplementary Data 1 and 3. *n* = 97/*n* = 99 biologically independent samples for MetS and controls, respectively. **b** Whole-genome analysis of four contigs that belong to the VCs marked by red arrows in a. The top and bottom contigs are zoomed in on the prophage region. The read coverage depth of these contigs in samples where they are present/absent is depicted in the graphs at the top and bottom. The nine genes shared by all *Candidatus Heliusviridae* are colored red, and numbered as follows: 1: DUF2800-containing, 2: DUF2815-containing, 3: DNA polymerase I, 4: nuclease (VRR-NUC-containing), 5: SNF2-like helicase, 6: terminase large subunit, 7: portal protein, 8: Clp-protease, 9: major capsid protein. Source data are provided as a Source Data file.

The above results all indicate that MetS gut bulk viromes are distinct from those in healthy individuals. In light of this, we surveyed our cohort with ANCOM-BC for individual VCs that were correlated with bulk viromes in either MetS or healthy controls. This uncovered thirty-six VCs that were more abundant in MetS participants, and sixteen more in controls (*q* ≤ 0.05, Fig. 4a).

In line with the above findings that *Bacteroidaceae* VCs are hallmarks of the MetS bulk virome, six of the seventeen MetS-associated VCs with a positive host prediction infected this family. One of these (VC_1838_0) contained a non-prophage contig (*i.e*., no detected bacterial contamination) of 34,170 bp with a checkV^29^ completion score of 100%. It further co-clustered with a contig that checkV identified as a complete prophage flanked by bacterial genes. Analysis with the contig annotation tool (CAT^37^) identified this contig as *Bacteroides fragilis*. Additionally, the most complete VC_1838_0 contig shared 6/69 (8.7%) ORFs with *Bacteroides uniformis Siphoviridae* phage Bacuni_F1^38^ (BLASTp bit score ≥ 50). Besides this, none of the contigs shared marked homology with any isolated phages found in the NCBI nucleotide databases (nr/nt). Some of them did, however, show significant similarity (BLASTn bit score ≥ 50) to phage genomes from an earlier publication by Tisza et al.^39^ studying a large phage database in relation to various diseases. Most notably, the largest contig from VC_977_13 (checkv completeness 90.32%) was identical over 99.98% of its genome to a phage that Tisza et al. determined to be significantly associated with fatty liver and atherosclerosis, both diseases related to MetS. We found similar results (with 78% aligned nucleotides from a complete genome) for *Bacteroidaceae* VC_1838_0, of which the most similar Tisza et al. genome was related to atherosclerosis and cirrhosis, as well as for VC_1221_0 (with 62% aligned nucleotides from an 83% complete genome), where relations to atherosclerosis and obesity were found. These disease correlations from independent cohorts support our findings linking these *Bacteroidaceae* VCs to MetS.

### A widespread phage family contains markers for healthy and MetS viromes

Besides the above-mentioned *Bacteroidaceae* VCs, all other differentially abundant VCs with host links, twenty-six MetS- and nine control-associated, infected *Firmicutes*, particularly in the *Clostridiales* order. The sole exceptions to this remarkably had CRISPR protospacer matches to multiple phyla: either *Firmicutes* and *Proteobacteria*, *Fimicutes* and *Bacteroidetes*, or *Firmicutes*, *Bacteroidetes* and *Actinobacteria* (Fig. 4a). Though this might result from taxonomically closely related phages that infect taxonomically distant hosts, we also observed one genome fragment in VC_1766_1 that had CRISPR spacer hits from hosts in multiple phyla. This indicated that this may be a phage with an extraordinarily broad host range.

Besides this broad host range VC, our attention was drawn to MetS-associated *Clostridiales* VC_818_0 and VC_1639_0. Both were predicted to infect hosts from Clostridium clusters IV and XIVa^40^, which are usually associated with healthy gut microbiomes. Further examination of their largest genomes revealed that they were remarkably similar to each other and to two VCs that were significantly associated with healthy controls: *Faecalibacterium/Clostridium methylopentosum* VC_1801_0 and *Oscillibacter/Ruminococcaceae* VC_803_0 (Fig. 4b).

Intrigued by this apparent relatedness of VCs that included markers of MetS and healthy controls among our cohort, we sought to identify additional related sequences among our cohort. For this, we first determined the exact length of a full VC_818_0 genome by analyzing read coverage plots of a prophage flanked by bacterial genes (Fig. 4b). By analyzing coverage of the contig in subjects where bacterial genes were highly abundant but viral genes were absent, we extracted a genome of 68,665 bp long. Homology searches of all 74 ORFs encoded by this prophage against all ORFs from all phage contigs in the cohort identified 261 contigs of over 30,000 bp that all shared nine genes (BLASTp bit score ≥ 50, Fig. 4b), including thirteen assembled from VLP datasets. Additionally, we identified 61 *Siphoviridae* phage genomes in the National Center for Biotechnology Information (NCBI) nucleotide database that also shared these nine genes. With one exception, these were *Streptococcus* phages, the exception being *Erysipelothrix* phage phi1605.

The genes shared by all these phage genomes formed three categories. First are genes encoding structural functions: a major capsid protein, portal protein, CLP-like prohead maturation protease, and terminase. The second group are transcription-related genes encoding a DNA polymerase I, probable helicase, and nuclease. Finally, there are two genes that encode domains of unknown function, but which given their adjacency to the second group are likely transcription-related.

**Fig. 5:**
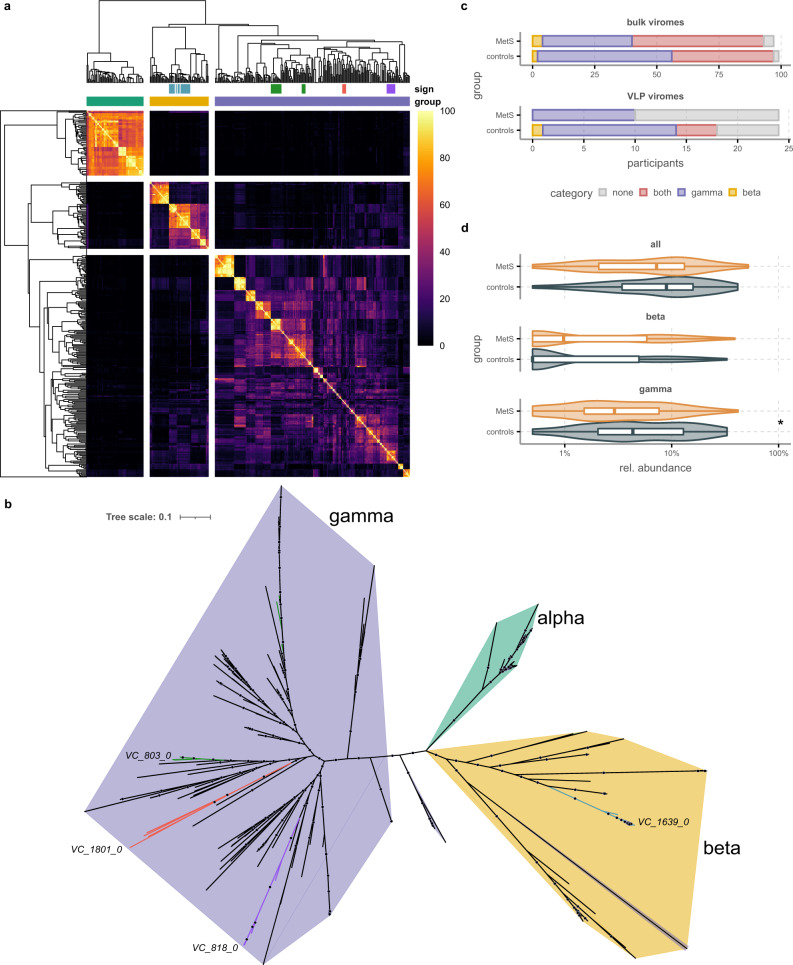
Three VCs that are hallmarks for either MetS or healthy control viromes are part of the widespread *Candidatus Heliusviridae* family of gut phages. **a** heatmap and hierarchical clustering of pairwise shared protein cluster values for 261 contigs from the current study and 61 previously isolated phages that all shared the same nine core *Ca. Heliusviridae* genes (blastp > 50). The dendrogram is cut to form three clusters, which are color coded above the heatmap as *Ca. alpha-* (green), *beta-* (yellow), and *gammaheliusvirinae* (purple). The top row of colors beneath the dendrogram denote the differentially abundant VCs, from left to right: VC_1639_0 (blue), VC_803_0 (green), VC_1801_0 (red), and VC_818_0 (purple). The legend denotes percent of total protein clusters that are shared. As some core genes formed several protein clusters, values can be below 10%. **b** An unrooted approximate maximum-likelihood tree built from a concatenated alignment of nine genes shared by all genomes in **a**, with colors defining subfamily membership according to **a**, and with the VCs significantly differentially abundant in either MetS or controls denoted. Dots on tree branches signify bootstrap values ≥95. **c** the prevalence of the *Candidatus Heliusviridae* groups among bulk and VLP phage populations. **d** The relative abundances of the *Candidatus Heliusviridae* and the groups in bulk phage populations. *n* = 97/*n* = 99 biologically independent samples for MetS and controls, respectively. *Q* values are denoted as follows * ≤ 0.05, ** ≤ 0.01, *** ≤ 0.001, **** ≤ 0.0001. Box plots show the median (middle line), 25th, and 75th percentile (box), with the 25th percentile minus and the 75th percentile plus 1.5 times the interquartile range (whiskers), and outliers (single points). Source data are provided as a Source Data file.

Earlier studies have used a cutoff of 10% gene similarity for phages that are in the same families, 20% for subfamilies, and 40% for genera^41,42^, while the international committee for the taxonomy of viruses (ICTV) proposes that phages that form a monophyletic group and share a significant number of genes constitute a family^43^. The nine shared genes form 10-25% of ORFs found on both the characterized phages and non-provirus contigs with checkV ‘high-quality’ designations. We thus tentatively classify these phages as a family, which we dubbed the *Candidatus Heliusviridae*. Next, we further studied the interrelatedness of *Ca. Heliusviridae* phages by performing pairwise blastp searches for all genes. The resulting bit-score table was then used to form protein clusters^27^, from which we calculated the pairwise percentages of shared protein clusters. Hierarchical clustering of the results showed that *Ca. Heliusviridae* phages form three groups (Fig. 5a). As the complete genomes in these groups shared less than 70% average nucleotide identity across their genome (median: 28.9%, 48.7%, and 21.8%, Fig. 5a), and following proposed guidelines^43^, these clusters form subfamilies. We thus designated them the *alphaheliusvirinae, betaheliusvirinae*, and *gammaheliusvirinae*. We confirmed these findings by building a concatenated approximate maximum-likelihood phylogenetic tree from alignments of nine conserved *Ca. Heliusviridae* genes. This also showed three main clades that almost completely aligned with the three groups based on shared protein cluster content (Fig. 5b, Supplementary Data 6 and 7).

Members of the *Ca. Heliusviridae* were present in the bulk phage populations of 190/196 participants (96.9%), 97 controls and 93 MetS participants (Fig. 5c). Among datasets of VLP phage populations, *Ca. Heliusviridae* phages were found in 25/48 participants (52.1%), 16 controls and 9 MetS, thus precluding the notion that they are defective prophages. It furthermore revealed that this phage family is a part of the core human gut microbiome. To validate our findings, we used three independent cohorts: the phage database constructed by Tisza et al. mentioned above^39^ and one cohort each studying gut virome relations to hypertension^44^ and type 2 diabetes^11^. To allow for incomplete assemblies, we searched for contigs in these three cohorts that contain the four conserved *Ca. Heliusviridae* structural genes. A phylogenetic tree containing concatenated alignments of the structural genes revealed two things. First, it clearly showed that contigs from all validation cohorts were interspersed among both *Ca. beta- and gammaheliusvirinae*. Second, the presence of divergent clades which did not contain any of the genomes in which earlier we identified all nine characteristic *Ca. Heliusviridae* genes hinted at further extensive diversity of the phage family (Supplementary Fig. 6). Among the gut viromes from an earlier cohort composed of school-aged children, of which 10 were controls, 10 were obese, and 8 had MetS, we further found Ca. Heliusviridae in 7/10 controls, while among obese and MetS they were present in 4/10 and 4/8, respectively.

Among the two cohorts studying hypertension and type 2 diabetes, *Ca. Heliusviridae* phages were present in 137/196 (69.9%, hypertension) and 98/145 (67.6%, T2D) participants (Supplementary Fig. 8). Meanwhile, for the 775 contigs with the four *Ca. Heliusviridae* structural genes, Tisza et al. previously determined the prevalence in the human microbiome project^45^. The data pertaining to this provided by Tisza et al. indicated that three individual *Ca. Heliusviridae* genomes found among their phage database were present in over 50% of human microbiome project participants, of which two had a prevalence of over 80%. Thus, not only are *Ca. Heliusviridae* phages as a family widespread in the human microbiome, several individual phage strains within it may be highly prevalent. In addition to prevalence, Tisza et al. also tested links between phages and various disease states. Among the *Ca. Heliusviridae* phages derived from this database, we found 74 that were previously significantly linked to obesity, and a further 82 related to various other cardiovascular diseases (non-alcoholic fatty liver/steatohepatitis, atherosclerosis, and type 2 diabetes). Our findings relating *Ca. Heliusviridae* phages to MetS are thus in line with findings relating to the Tisza et al. phage database.

### *Ca. Heliusviridae* subfamilies have distinct relations to MetS

The *Ca. alphaheliusvirinae* solely contained previously isolated *Streptococcus* phages, which both in the hierarchical clustering and the phylogenetic tree were distinct from the other genomes. Meanwhile, three of the four VCs that were significantly associated with either MetS (1) or controls (2) where part of the *Ca. gammaheliusvirinae*, by far the largest and most diverse group. Two of these, VC_818_0 and VC_1801_0, formed monophyletic clades in both hierarchical clustering and phylogenetic tree. Meanwhile, VC_803_0 was conversely spread out over multiple clades, indicating it was more heterogenous than the other two.

Of the subfamilies, phages in the *Ca. gammaheliusvirinae* were the most prevalent, being present in the bulk phage populations of 95 controls and 88 MetS participants. These phages were also significantly more abundant in the controls (Wilcoxon signed-rank test, *p* = 0.011, Fig. 5d) as a whole, despite the fact that in contains the MetS-associated VC_818_0. Among VLP populations, we also identified them in 15/24 controls and 9/24 MetS participants, though there was no significant difference in abundance. The bacterial hosts of these phages were predicted to be within various families in the *Clostridiales*, as well as the *Veillonellales, Coriobacteriales*, and *Acidaminacoccales*.

While less prevalent than *Ca. gammaheliusvirinae* phages, *Ca. betaheliusvirinae* phages were still identified in the bulk phage populations of 44 controls and 57 MetS participants (Fisher’s exact test *p* = 0.047, Fig. 5c), though they were not significantly more abundant in the latter (Wilcoxon signed rank test, *p* = 0.063). Remarkably, *Ca. betaheliusvirinae* phages were completely absent from MetS VLP phage populations whereas they were present in 6/24 controls, making the difference in prevalence significant (Fisher’s exact test *p* = 0.022). These results show that Ca. *Heliusviridae* phages are part of both the core human gut bulk and VLP viromes. Counter to *Ca. gammaheliusvirinae*, all host predictions of *Ca. betaheliusvirinae* phages were within the *Clostridiales*. In summary, *Ca. gammaheliusvirinae* is the largest and most prevalent subfamily of *Ca. Heliusviridae* phages, which as a whole is more related to the healthy human virome, while *Ca. betaheliusvirinae* phages are more prevalent in MetS bulk viromes but depleted among VLP populations.

### MetS-associated *Ca. gammaheliusvirinae* prophages encode possible metabolic genes

**Fig. 6:**
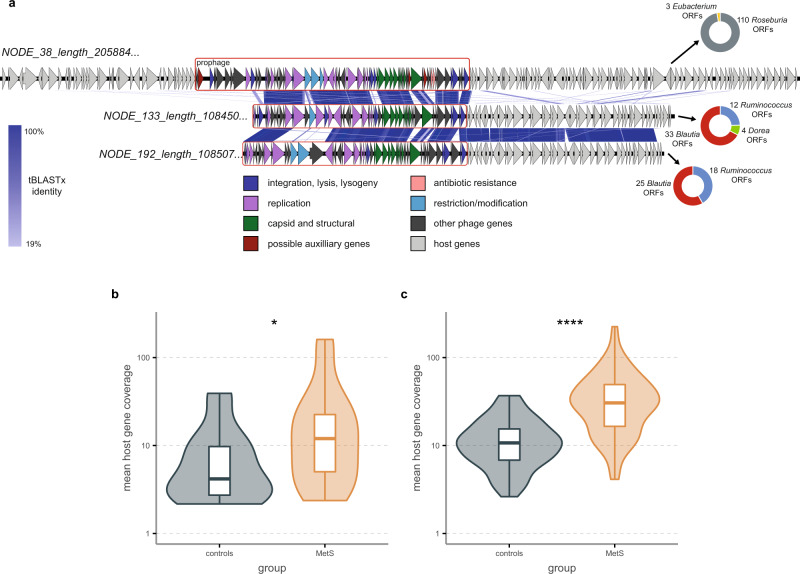
VC_818_0 infects *Roseburia* and *Blautia*, and carries possible auxiliary metabolic genes. **a** Whole-genome alignment of three prophages contained within VC_818_0, with pie charts denoting the top BLASTp hit of all host genes on the contigs. The mean coverage of host-derived regions in NODE_38 (*p* = 0.042) (**b**) and NODE_192 (*p* = 5.1 × 10^−^^4^) (**c**). *n* = 97/n = 99 biologically independent samples for MetS and controls, respectively. Significance according to two-sided Wilcoxon signed-rank test, p-values are denoted as follows * ≤ 0.05, ** ≤0.01, *** ≤ 0.001, **** ≤ 0.0001. Box plots show the median (middle line), 25th, and 75th percentile (box), with the 25th percentile minus and the 75th percentile plus 1.5 times the interquartile range (whiskers), and outliers (single points). Source data are provided as a Source Data file.

Members of the *Ca. Heliusviridae* are generally linked to bacteria that are associated with healthy human gut microbiomes. It is thus an apparent contradiction that *Ca. Heliusviridae* VC_818_0 (*Ca. gammaheliusvirinae*), which is associated with MetS viromes, contains phages that infect *Roseburia*, which is a short chain fatty acid producer and is often abundant in healthy microbiomes^46^. Due to this contradiction, we explored the phages in this VC further. These included two additional prophages, which where both incomplete (Fig. 6a, Supplementary Data 4). Whole-genome alignment showed that all three prophages shared their phage genes, and that the two incomplete ones also shared host-derived genes. Homology searches of the bacterial host ORFs found on these two contigs against the NCBI nr database (BLASTp, bit score ≥50) showed that the most common top hits were *Blautia*, and for the plurality *Blautia wexlerae* (Fig. 6a). Thus, VC_818_0 likely contains temperate phages with narrow host ranges that infect bacteria spread out across at least two genera within the *Lachnospiraceae*.

To examine if the hosts infected by VC_818_0 phages were more abundant in MetS participants, we determined mean coverage of bacterial genes found adjacent to the prophages. We thus assured that we analyzed the particular host strains infected by these phages, rather than unrelated strains in the same genera. This showed that both the *Blautia* and the *Roseburia* host genes were more abundant among MetS participants (Wilcoxon signed-rank test, *Blautia p* = 5.1 × 10^−4^, *Roseburia p* = 0.042, Fig. 6b, c). The specific *Lachnospiraceae* strains infected by VC_818_0 phages thus seem to thrive in MetS microbiomes. This could in part be due to functions conferred upon these bacteria by these prophages, as particularly the *Roseburia* prophage which carried several virulence- and metabolism-related genes, including ones encoding a chloramphenicol acetyltransferase 3 (2.3.1.28), Glyoxalase/Bleomycin resistance protein (IPR004360), multi antimicrobial extrusion protein (IPR002528), 2-succinyl-6-hydroxy-2,4-cyclohexadiene-1-carboxylate synthase (4.2.99.20), and NADPH-dependent FMN reductase (PF03358). The latter two in particular are both associated with vitamin K (menaquinone) metabolism, which is part of (an)aerobic respiration in bacteria^47^. We speculate that this opens up the possibility that this *Roseburia* prophage aids its host bacterium, which in turn may contribute to MetS phenotypes.

## Discussion

This is the first study of adult gut viromes in the context of MetS, a widespread global health concern to which the gut bacteria targeted by phages are believed to be a main contributor^18^. We have shown that MetS is associated with decreases in gut bulk virome total relative abundance and richness, but not in evenness. Due to their compositional nature, these virome alterations could be bacterially driven, as phage total relative abundance decreases could be caused by bacterial counts increasing rather than phage counts decreasing. But since we measured decreased bacterial richness and evenness, MetS gut metagenomes would need to have larger numbers of bacterial cells that are distributed among fewer strains that are more unevenly divided than in healthy individuals. Conversely, total phage relative abundances could be lower in MetS due to lower viral loads, which would be in line with decreased phage richness and is in agreement with recently reported direct correlations between gut viral and bacterial populations in healthy individuals^48^. Future confirmation of this would necessitate counts of viable bacterial cells and VLP. In either case, we surmise that the main driver of these effects is diet, which affects bacterial^49–51^ as well as viral^52^ populations. It is also possible that phage populations as described here may further exacerbate bacterial diversity losses, as low phage abundance may decrease their positive effects on bacterial diversity^53,54^. Our findings of increased richness and diversity in the bulk viromes were in line with a recent study of MetS among 28 school-aged children^23^. Interestingly, their results pertained to VLP datasets, which in our study showed no significant differences in richness and diversity. This could reflect the difference in cohort size, as we analyzed double the number of participants, or the previously reported changes in the gut virome with increasing age^14^.

We further found strong negative correlations between the risk factors that constitute MetS and bulk phage richness, but not evenness. This likely stems from the nature of bulk viromes, which reflect phages that are actively engaging with their hosts. As phages that target depleted bacteria are more likely to be low in abundance and extracellular, they are not observed among bulk viromes. Thus, the apparent species richness drops because low abundant extracellular phages are below the detection limit of our sequencing approach. This removal of rare phages in turn prohibits significant drops in species evenness in MetS. It could also be that bacteria depleted in MetS reside in phage-inaccessible locales within the gut^55^, which perhaps results in removal of the corresponding phages from the gut to below detectable levels. This would explain the stronger correlation between bacterial evenness than richness to MetS risk factors.

As most (gut) phages remain unstudied^14,56^, it is often difficult to link phages to host bacteria^57^. Here, we linked roughly one third of all VCs to a bacterial host. The remaining majority of VCs likely represent phages that infect bacterial lineages lacking CRISPR systems^58^, or that integrate into hosts which we could not taxonomically classify. Whichever is the case, our study underscores the great need for methods that link phages to hosts with high accuracy^59,60^. From the phage-host linkages that we obtained, we found that VCs containing phages infecting specific bacterial families tend to be either depleted (*Bifidobacteriaceae)* or enriched (*Streptococcaceae* and *Bacteroidaceae*) in tandem to their hosts. We notably found that several other bacterial families (*Enterobacteriaceae, Lachnospiraceae, Ruminococcaceae, Rikenellaceae*, and *Clostridiaceae*) were either significantly depleted or elevated in MetS microbiomes, but the accompanying phages were not. Though this could reflect an unevenness in predation by phages among the various bacterial families in the gut, it more likely results from the inability to link the majority of VCs to bacterial hosts, as mentioned above.

The identification of *Bifidobacteriaceae* bacteria and their phages as more abundant among healthy controls is in line with established studies that show depletion of these families in MetS^22^ and MetS-associated disease states^34^. Phages infecting both the *Bifidobacteriaceae* as a whole and specific *Bifidobacteria* species were strikingly only elevated in abundance among bulk viromes. Their absence among VLP populations may imply a preference of *Bifidobacteriaceae* gut phages toward intracellular lifestyles. This in turn could explain the dearth in isolated virulent *Bifidobacterium* phages when compared to other *Actinobacteria* lineages^61^. For the MetS-associated host families, *Streptococcaceae* are known to be more abundant in obesity-related ilnesses^34^. Within the *Bacteroidaceae*, the *Bacteroides* are often positively associated with high-fat and high-protein diets^62,63^. Simultaneously, however, reports disagree on individual *Bacteroides* species and their associations with MetS-related diseases like obesity, type 2 diabetes, and non-alcoholic fatty liver disease^34^. Such conflicting reports likely reflect the large diversity in metabolic effects at strain level among these bacteria^64^. Based on our results, we drew two conclusions. First, that *Bacteroidaceae*-linked VCs mirror their hosts in MetS-associated relative abundance increase, and second that *Bacteroidaceae*-linked VCs are of significant interest to studies of the MetS microbiome. The latter conclusion is strengthened by findings that *Bacteroides* prophages can alter bacterial metabolism in the gut^8^.

While *Bacteroidaceae* VCs at large were thus seemingly associated with MetS phenotypes, we did not find higher abundance of *Crassvirales* phages in MetS. However, we did find higher prevalence of these phages in the bulk viromes of healthy controls. This widespread and often abundant human gut phage family infects *Bacteroidetes*, including members of the *Bacteroidaceae*^65,66^. As these phages are commonly linked to healthy gut microbiomes^42,66,67^, it is conceivable that they would be negatively correlated with MetS viromes. But due to the great variety within this family^66^, and perhaps also the hypothesized aptitude of *Crassvirales* phages for host switching through genomic recombination^66^, more detailed study is needed to elucidate the exact links of this family to MetS gut viromes despite the apparent elevated abundance of their hosts.

Finally, our study revealed the *Candidatus Heliusviridae*, a highly widespread family of gut phages that largely infect *Clostridiales* hosts. This prospective family is also expansive, and includes at least three distinct groupings. Our uncovering of this human gut phage family underscores the usefulness of database-independent de novo sequence analyses^27,30,68^, as well as the need for a wider view on viral taxonomy than has presently been exhibited in the field of gut viromics.

The *Ca. Heliusviridae* are of particular interest to studies of MetS and related illnesses because its member phages include some associated with MetS and others with healthy controls. Most striking is the fact that most of the bacteria infected by MetS-associated *Ca. Heliusviridae* phages are generally producers of short chain fatty acids (SCFA) such as butyrate and commonly depleted in MetS^34^. Such SCFA-producing bacteria are commonly positively associated with healthy microbiomes, as SCFAs that result from microbial digestion of dietary fibers have a role in the regulation of satiation^69,70^. The exception to this is the *Veillonellaceae* that is infected by a phage the *Ca. gammaheliusvirinae*, which displays elevated abundance in non-alcoholic fatty liver disease^34^. While higher abundance of some of the other butyrate-producers infected by *Ca. Heliusviridae* phages is associated with metformin use^71^, this is used to treat type 2 diabetes rather than MetS.

Particularly interesting are the *Roseburia/Blautia* phages in VC_818_0, which was the most strongly correlated with MetS out of all VCs. The positive correlation between the relative abundance of these phages and that of their hosts indicates that they have a stable relation with their hosts in the MetS microbiome. This is to be expected, as large-scale prophage induction is generally associated with sudden alterations to the microbiome, such as the addition of a specific food supplement that acts as an inducer of prophages^9^. Such sudden alterations in phage behavior are unlikely to be captured in large cohorts with single measurements. In fact, as phages are strongly dependent on their host, one might expect the abundance of many gut phages to be positively correlated to that of their particular hosts under the relatively temporally stable conditions of MetS. The strong correlation of VC_818_0 to MetS phenotypes, coupled to the commonly found correlation to healthy microbiomes of VC_818_0 host bacteria, and the presence of potential auxiliary metabolic genes in VC_818_0 phage sequences combined introduce the possibility that prophage formation of these *Ca. Heliusviridae* phages alters the metabolic behavior of their host bacteria, as is known to happen in marine environments^72,73^. This could make these bacteria detrimental to health. Proving this hypothesis necessitates future isolation of VC_818_0 phages.

Despite efforts to catalog the human gut virome^14,32^, taxonomically higher structures are still largely absent. This study shows the worth of analyzing phages at higher taxonomic levels than genomes or VCs, similarly to what has been shown in recent years regarding the *Crassvirales* phage order^15,16^. Unlike the *Crassvirales*, however, *Ca. Heliusviridae* phages seem to be strongly correlated with human health. We hope that further research will provide a deeper understanding of the effect that these phages have on their bacterial hosts and the role that this plays in MetS, as well as a refinement of their taxonomy.

## Methods

### Whole-genome shotgun sequencing

The Healthy Life in an Urban Setting (HELIUS) cohort includes some 25,000 ethnically diverse participants from Amsterdam, the Netherlands. The cohort details were published previously^26^. The HELIUS cohort conformed to all relevant ethical considerations. It complied with the Declaration of Helsinki (6th, 7th revisions), and was approved by the Amsterdam University Medical Centers Medical Ethics Committee. All participants provided written informed consent. For details on stool sample collection from among the participants, their storage, and DNA extraction, see Deschasaux et al.^74^. In summary, participants were asked to deliver stool samples to the research location within 6 h after collection with pre-provided kit consisting of a stool collection tube and safety bag. If not possible, they were instructed to store their sample in a freezer overnight. Samples were stored at the study visit location at −20 °C until daily transportation to a central −80 °C freezer. Total genomic DNA was extracted using a repeated bead beating method described previously^74,75^. Libraries for shotgun metagenomic sequencing were prepared using a PCR-free method at Novogene (Nanjing, China) on a HiSeq instrument (Illumina Inc. San Diego, CA, USA) with 150 bp paired-end reads and 6 Gb data/sample. All bioinformatics software was run using standard settings, unless otherwise stated.

Following previously set definitions^76^, participants were classified in the MetS group if three of the following five health issues occurred: abdominal obesity measured by waist circumference, insulin resistance measured by elevated fasting blood glucose, hypertriglyceridemia, low serum high-density lipoprotein (HDL), and high blood pressure^76^. All participants of the HELIUS cohort reside in Amsterdam, the Netherlands. Participants were roughly evenly divided by ethnicity, with European Dutch comprising 49 controls and 49 MetS participants, and African Surinamese 50 controls and 49 MetS participants. The MetS group contained 55 women and had a median age of 58 (mean 56.8 ± 8.09), and the controls 71 and had a median age of 50 (mean 49.1 ± 12). Of the 196 participants, 26 used metformin, of whom 2 were controls who did not concur to the MetS criteria.

### VLP isolation and DNA extraction

To gain a full understanding of the dsDNA virome in the current cohort, we performed viral-like particle (VLP) sequencing on fecal matter from a subset of 48 participants. These included 24 controls and 24 MetS participants, with each group being composed of 12 European Dutch and 12 African Surinamese persons. This sub-selection was balanced for age (controls 55.9 ± 8.47, MetS 58.7 ± 7.05, Wilcoxon signed-rank test, *p* = 0.27) and sex (controls 14 women, MetS 14 women).

Studies of the VLP fractions were modelled after Garmaeva et al.^77^ and Shkoporov et al.^78^. First, 0.5 g of feces were resuspended in 5 ml of sterile SM buffer (100 mM NaCl, 8 mM MgSO_4_ × 7H_2_O, pH 7.5), chilled on ice for 10 min and centrifuged at 27,000 × *g* for 10 min at 4 °C. Supernatant was collected and filtered through a 0.45 µm pore polyethersulfone membrane filter, whereafter the volume of the filtrate was adjusted to 5 ml. Next, free DNA was digested by incubating the VLPs with 5 µl 2.5 U/µl of DNase I (ThermoFisher Cat#R0561) and 555 µl of 10× DNase buffer at 37 °C for 1 h. VLPs were lysed by the addition of 100 µl of 100 mg/ml SDS (Invitrogen Cat#1.5525.017) and 2.5 µl of 20 mg/ml proteinase K (Promega Cat#MC5005) to the samples, which were incubated at 56 °C for 1 h.

Nucleic acids were purified using a two-step phenol/chloroform extraction protocol. First, samples were extracted by mixing with an equal volume (5.7 ml) of phenol/chloroform/isoamyl alcohol 25:24:1 (Sigma Cat#77617) followed by centrifugation at 4000 × *g* for 10 min at room temperature. Subsequently, 5.2 ml of the aqueous upper phase was mixed with an equal volume of chloroform (Merck Cat#102445) and again centrifuged as described above. To precipitate the nucleotides, 4.7 ml of aqueous phase was mixed with 470 µl 3 M sodium acetate (pH 5.2), 4.7 µl glycogen (ThermoFisher Cat#R0561) and 14.2 ml ice-cold absolute ethanol (Merck Cat#100983) and incubated at −20 °C for 1–2 h. Samples were centrifuged at 21,000 × *g* for 15 min at 4 °C, after which the pellet was washed with 500 µl 70% ethanol. After air drying the pellet for ~20 min, the pellet was resuspended in 500 µl ultrapure RNase/DNase-free water (ThermoFisher Cat#10977-035). The resulting solution was subjected to a final round of purification using the DNeasy Blood&Tissue kit (Qiagen Cat#69506) according to the manufacturer’s protocol, with a final elution volume of 100 µl.

### Metagenomic sequencing of VLP DNA

Next, library preparation was performed using the NEBNext Ultra II FS DNA library prep kit (New England Biolabs Cat#E7805L), complemented with the NEBNext Multiplex Oligos for Illumina (New England Biolabs Cat#E7600S) dual indexes according to the manufacturer’s protocol. Fragmentation with the FS enzyme mix was performed for 5 minutes and the NEB adapters for Illumina were diluted 10 times to prevent dimer formation due to the low input DNA concentrations. After adapter ligation, DNA fragments of 300–500 bp were purified and subsequently amplified with 10 PCR cycles during the PCR enrichment step. After final clean-up, the quality and concentration of the VLP libraries were assessed with the Qubit dsDNA HS kit (ThermoFisher Cat#Q32854) and with the Agilent High Sensitivity D5000 ScreenTape system (Agilent Technologies). Libraries were sequenced using 2 × 150 bp paired-end chemistry on an Illumina NovaSeq 6000 platform with the S4 Reagent Kit v1.5 (300 cycles).

### Read trimming and contig assembly

For both WGS and VLP datasets, post-sequencing data analysis was identical. Analysis of sequencing output started with adapter trimming and quality control of sequencing reads using fastp v0.23.1^79^, using standard settings. Trimmed reads were mapped to the human genome (GRCh37) using bowtie2 v2.4.0^80^, which showed that samples contained 0.13 ± 0.26 % human reads. High-quality reads were then assembled per sample (*i.e*., 196 WGS and 48 VLP assemblies) into contigs using the metaSPAdes v3.14.1 software^81^. For each sample, we selected contigs of more than 5,000 bp for further analysis. In addition, among contigs between 1,500 and 5,000 bp we identified circular contigs by checking for identical terminal ends using a custom R script that employed the Biostrings R package v3.12^82^. Assemblies yielded a total of 9,108,147 circular contigs and contigs over 5,000 bp. Three VLP samples were subsampled differently due to memory issues encountered in assemblies. These were S038 and S192 (subsampled to 40 million read pairs), and S069 (subsampled to 25 million read pairs).

### Phage and bacterial sequence selection

For phage sequences we followed Gregory et al.^83^. We first analyzed contigs using VirSorter v1.0.6^84^, which analyses both distant protein homologies to viral hallmark genes and genome architecture, and selected those in category 1, 2, 4, and 5. In parallel, contigs were analyzed using VirFinder v1.1, which predicts viral sequences with a machine-learning approach, after which we selected those with a score above 0.9 and a p-value below 0.05. We additionally classified contigs as phage if (I) they were both in VirSorter categories 3 or 6 and had VirFinder scores above 0.7 with p-values below 0.05, and (II) annotation with the contig annotation tool (CAT) v5.1.2^37^, which classifies contigs using blastp against the NCBI nr protein database, was as “Viruses” or “unclassified” at the superkingdom level. After removing those with CAT classifications as Eukaryotic viruses, this resulted in a database of 45,568 phage contigs. Bacterial sequences were predicted by selecting all contigs that CAT annotated in the “Bacteria” at the superkingdom level, and removing contigs that were also found in the phage dataset. An exception was made for prophage contigs in VirSorter category 4, 5, and 6, which were left among the bacterial dataset (see “Phage-host linkage prediction”). This resulted in a total of 1,579,361 bacterial contigs. The 1,624,929 bacterial and phage datasets were then concatenated and deduplicated using dedupe from BBTools v38.84 with a minimal identity cutoff of 90% (option minidentity = 90). This identified 759,403 duplicates and resulted in 829,633 non-redundant bacterial sequences and 25,893 non-redundant phage sequences. While the bacterial sequences were used for host prediction (see “Phage-host linkage prediction”), we subsequently predicted open reading frames (ORFs) in phage contigs using Prodigal v2.6.2^85^ (option -p meta). These ORFs were then used to group phage sequences in viral clusters (VCs) using vContact2 v0.9.18^27^. For a full accounting of phage contigs, see Supplementary Data 1 and 3. All phage contigs were analyzed for completion with CheckV v0.7.0–1^29^ (Supplementary Data 5).

To test the robustness of the metagenomic sequencing, we also analyzed quality trimmed reads from the bulk sequencing samples with metaphlan v3.0.13 using standard settings. This analysis identified a total of 632 bacterial species across all samples (mean: 88.7 ± 15.7 species/sample, median: 90). Based on the output, richness had a significance of 0.035, Pielou evenness 0.027, and Shannon diversity 0.0015 (according to Wilcoxon signed rank test).

### Read mapping and community composition

For bacterial community composition, we used sequencing data targeting the V4 region of the 16s rRNA gene that had been performed previously^74,86^. Details on ASV construction from these samples was described previously in Verhaar et al.^86^. As part of this previous analysis, samples with fewer than 5000 read counts had been removed, and samples had been rarified to 14932 counts per sample.

To determine phage community composition, we mapped reads from each sample to the non-redundant contig dataset using bowtie2 v2.4.0^80^. As previously recommended^30^, we removed spurious read mappings at less than 90% identity using coverM filter v0.5.0 (unpublished; https://github.com/wwood/CoverM, option -min-read-percent-identity 90). The number of reads per contig was calculated using samtools idxstats v1.10^87^. As was also recommended^30^, contig coverage was calculated with bedtools genomecov v2.29.2^88^, and read counts to contigs with a coverage of less than 75% were set to zero. Read counts for each sample were finally summed per VC. For analyses of alpha- and beta-diversity, we adjusted read counts for contig length and library size by calculating reads per kilobase per million mapped reads (RPKM). Where samples were directly comparted, RPKM values were made compositional by dividing them by the total RPKM per sample. On average, 2.71 ± 1.3% of WGS reads mapped to viral sequences (median 2.38%), along with 45.3 ± 20.4% (median 41.8%) of VLP reads.

### Ecological measures

In all boxplots, we tested statistical significance using the Wilcoxon rank-sum test as it is implemented in the ggpubr v0.4.0R package (available from: https://cran.r-project.org/web/packages/ggpubr/index.html). Unless stated otherwise, all plots were made using either ggpubr or the ggplot2 v3.3.2R package (available from: https://cran.r-project.org/web/packages/ggplot2/index.html). Alpha diversity measures (observed VCs and Shannon H’ for phages and Chao1 and Shannon H’ for bacteria) were calculated using read count tables with the plot_richness function in the phyloseq R package v1.33.0^89^. For β-diversity, we converted read counts to relative abundances using the transform function from the microbiome v1.11.2R package. We then used the phyloseq package to calculate pairwise Bray-Curtis dissimilarities and construct a principal coordinates analysis (PCoA). Statistical significance of separation in the PCoA analysis was determined with a permutational multivariate analysis of variance (permanova) using the adonis function from the vegan R package^90^. For this analysis, we adjusted for smoking, sex, age, alcohol use, and metformin use. Direct correlation coefficients between richness and diversity were calculated using the stat_cor function in the ggpubr R package. The resulting P-values were adjusted for multiple testing using the Benjamini–Hochberg procedure.

### Phage-host linkage prediction

We predicted VC-bacterium links in three ways: (i) CRISPR protospacers, (ii) prophage similarity, and (iii) characterized phage similarity.

We predicted CRISPR arrays among the bacterial contigs using CRISPRdetect v2.4^91^ (option array_quality_score_cutoff 3) and used these to match bacterial contigs and phage contigs. In addition, we used a dataset of 1,473,418 CRISPR spacers that had previously been predicted^60,92^ in genomes contained in the Pathosystems Resource Integration Center (PATRIC)^93^ database. We matched CRISPR protospacers to viral contigs using BLASTn v2.12.0+^94^ with the short option. Spacer hits with less than 2 mismatches were considered valid. This process resulted in 155,173 spacer hits to PATRIC genomes or to bacterial contigs from this study with definite CAT classifications at the phylum level (Supplementary Data 2).

To identify predicted phage contigs with high sequence similarity to prophages, we analyzed which viral clusters contained on of the 7691 bacterial contigs with VirSorter prophage predictions in category 4 or 5. CAT was subsequently used to determine the taxonomy of bacterial contigs with prophage regions. In total, we linked 2,391 VCs to prophages with this approach.

Finally, VCs were linked to bacterial hosts by vContact2 clustering with characterized phages from the viral RefSeq V85 database^95^ with a known host. To achieve this, we selected all VCs from the vContact2 output that contained both characterized genomes and phage contigs. If all characterized phages infected hosts within the same bacterial family, we took that to mean that the whole VC infects hosts from that family. This approach linked 4457 VCs to hosts.

### Differential abundance analysis

To determine which bacteria and VCs were differentially abundant between MetS and control subjects, we employed the analysis of composition of microbiomes with bias correction (ANCOM-BC)^33^. This method, unlike other similar methods like DeSeq2, takes into account the compositional nature of metagenomics sequencing data^96^. To implement this method, we applied the ANCOM-BC v1.0.2 R package to raw read count tables, as ANCOM-BC employs internal corrections for library size and sampling biases^33^. Significance cutoff was set at an adjusted p-value of 0.05, *p* values were adjusted using the Benjamini–Hochberg method, and all entities (bacteria taxa/VCs) that were present in more than 10% of the samples were included (options p_adj_method = “BH”, zero_cut = 0.9, lib_cut = 0, struc_zero = T, neg_lb = F, tol = 1e-5, max_iter = 100, alpha = 0.05). For this analysis, we adjusted for smoking, sex, age, alcohol use, and metformin use.

### Crassvirales phages

To identify *Crassvirales* phages, we employed a methodology described earlier^42^, for which we first made a BLAST database containing all ORFs from all phage contigs (predicted before viral clustering, see “Viral and bacterial sequence selection”) using BLAST v2.9.0+^94^. We then performed two BLASTp searches in this database, one with the terminase (YP_009052554.1) and one with the polymerase (YP_009052497.1) of crAssphage (NC_024711.1), with a bit score cutoff of 50. All phage contigs that had (i) a hit against both crAssphage terminase and polymerase and a query alignment of ≥350 bp, and (ii) a contig length of ≥70 kbp were considered *Crassvirales* phages. This resulted in 287 *Crassvirales* phage contigs, which were contained in 88 VCs.

### Candidatus *Heliusviridae* analysis

To detect pairwise similarity, whole genome analyses were constructed with Easyfig v2.2.5^97^. The prophage borders in NODE_38_length_205884_cov_102.806990 were determined by determining the read depth along the entire contig from the bam files with read mapping data (“Read mapping and community composition”) using bedtools genomecov v2.29.2^88^ with option -bg. Resultant output was parsed and plotted in R. Other related phages among the cohort were detected by performing a BLASTp search with all phage ORFs of NODE_38_length_205884_cov_102.806990 against all phage ORFs of the cohort with Diamond v2.0.4. This identified nine genes that were present in 249 contigs. The ORFs on these contigs were annotated using PROKKA v1.14.6^98^ and InterProScan v5.48-83.0^99^. To identify isolated phages that share these nine contigs, we performed a BLASTp against the NCBI nr database using the NCBI webserver^100^ on February 26 2021 and collected all genomes with hits against all nine genes (bit score ≥ 50).

The phages sharing all nine genes were clustered by analyzing them with vContact2 v0.9.18^27^, extracting the protein clustering data and calculating the number of shared clusters between each pair of contigs. Contigs were clustered in R based on Euclidean distances with the average agglomeration method.

To build a taxonomic tree, the nine genes were separately aligned using Clustal Omega v1.2.4^101^, positions with more than 90% gaps were removed with trimAl v1.4.rev15^102^ and alignments were concatenated. From the concatenated alignment, an unrooted phylogenetic tree was built using IQ-Tree v2.0.3^103^ using model finder^104^ and performing 1000 iterations of both SH-like approximate likelihood ratio test and the ultrafast bootstrap approximation (UFBoot)^105^. Model finder selected LG + F + R8 as the best-fit substitution model. In addition, ten iterations of the tree were separately constructed, as has been recommended^106^ (IQ-Tree options -bb 1000, -alrt 1000, and—runs 10).

### Validation of *Ca. Heliusviridae* in other cohorts

We used three additional studies to analyze prevalence of the *Ca. Heliusviridae*; one composing of 145 participants used to study the gut virome in type 2 diabetes^11^, a second containing 196 participants and used to study the gut virome in hypertension^44^, and a final one thousands of phages from various sources^39^. Reads belonging to the former two studies were downloaded from the NCBI sequencing read archive (SRA) and assembled as described above, while for the latter assembled contigs were downloaded. After assembly, ORFs were predicted using Prodigal v2.6.2^85^. *Ca. Heliusviridae* members were identified by blastp using Diamond v2.0.4^107^ against ORFs from each study, in which the terminase, portal protein, Clp-protease, and major capsid protein of NODE_38_length_205884_cov_102.806990 were used as queries. This was done instead of all nine signature *Ca. Heliusviridae* genes to better allow for incomplete assemblies. Contigs containing all four genes were selected, and a concatenated alignment was made of the four head genes found in the T2D and hypertension cohorts, plus all *Ca. Heliusviridae* in the tree depicted in Supplementary Fig. 7. These were then used to build a phylogenetic tree. The concatenated alignment and phylogenetic tree were constructed as described above under “Candidatus Heliusviridae analysis”.

We further analyzed the data obtained by and earlier study of gut viromes in MetS among 28 school-aged children^23^. We downloaded reads from the NCBI sequencing read archive (sra). As this this project yielded an average 1.3 ± 0.9 M reads, we cross-assembled all 28 samples in one assembly with metaSPAdes with the same settings as described above (Read trimming and contig assembly). This yielded 45,112 contigs of more than 1,500 bp, with an average length of 3,702 bp. No contigs carrying all nine *Candidatus Heliusviridae* were identified, likely because this would require a contig of at least 20,000 bp. We thus performed a blastp using Diamond v2.0.4106 (bit score ≥ 50) against the terminase protein of NODE_38_length_205884_cov_102.806990, which identified 31 potential *Candidatus Heliusviridae* contigs.

### Statistics and reproducibility

All statistical analyses were performed in R v4.1.1. Details on the statistical tests that were applied are indicated in the figure captions and the results where necessary. The scripts used to perform statistical analyses are available in Supplementary Data 8. No statistical method was used to predetermine sample size. No data were excluded from the analysis. The experiments were not randomized. Participants were allocated into groups based on clinical measurements of metabolic syndrome-related clinical parameters. Therefore, the investigators were not blinded to allocation during experiments and outcome assessment.

### Reporting summary

Further information on research design is available in the Nature Research Reporting Summary linked to this article.

## Supplementary information

Supplementary Information

Description of Additional Supplementary Files

Supplementary Dataset 1

Supplementary Dataset 2

Supplementary Dataset 3

Supplementary Dataset 4

Supplementary Dataset 5

Supplementary Dataset 6

Supplementary Dataset 7

Supplementary Dataset 8

Reporting Summary

## Data Availability

The sequencing data generated in this study have been deposited in the European Genome-Phenome Archive database under accession code EGAS00001006260. The sequencing data are available under restricted access for restrictions imposed by the signed consent of participants, access can be obtained by submitting a proposal to the HELIUS Executive Board as outlined at http://www.heliusstudy.nl/en/researchers/collaboration, by email: heliuscoordinator@amsterdamumc.nl. The HELIUS Executive Board will check proposals for compatibility with the general objectives, ethical approvals and informed consent forms of the HELIUS study. There are no other restrictions to obtaining the data and all data requests will be processed in the same manner. The data generated in this study are provided in the Source Data file. The human genome data used in this study is available at the National centre for biotechnology information (NCBI) under accession GRCh37 [https://www.ncbi.nlm.nih.gov/assembly/GCF_000001405.13/]. The CRISPR spacer dataset derived from the PATRIC database is available from Supplementary Table 1 of ref. ^92^ [https://academic.oup.com/nar/article/48/21/12074/5997439#supplementary-data]. The reads from the validation cohorts are available from NCBI under the NCBI BioProject accession numbers PRJNA646512, PRJEB13870, PRJNA422434, and PRJNA573942. Source data are provided with this paper.

## Source data

Source Data

## Code availability

All code describing the statistical analyses performed in this work can be found in Supplementary Data 8. For direct access to the underlying data and participant metadata, see the Data availability statement above.
