# Supplementary material for: Gut virome profiling identifies a widespread bacteriophage family associated with metabolic syndrome
Source: Nat Commun. 2022 Jun 23;13:3594. doi: 10.1038/s41467-022-31390-5 (PMC9226167; doi:10.1038/s41467-022-31390-5)
Supplement: Supplementary file 1 — Supplementary Information [file 41467_2022_31390_MOESM1_ESM.pdf]

Supplementary Information: Gut virome profiling identifies a widespread bacteriophage family associated with metabolic syndrome

P.A. de Jonge et al.

Supplementary Figures

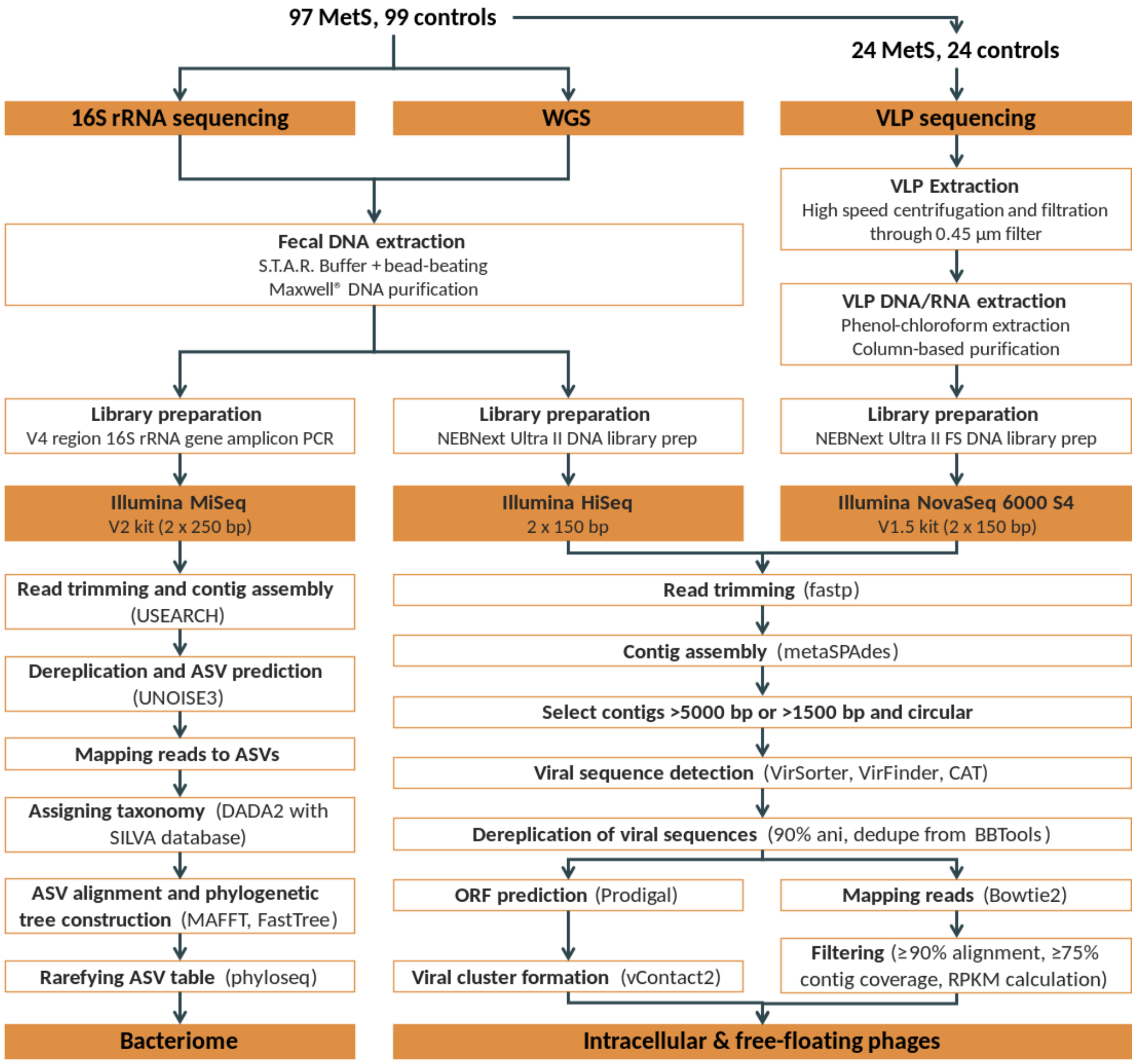

Supplementary Figure 1: Flow chart of the analyses preformed in this study.

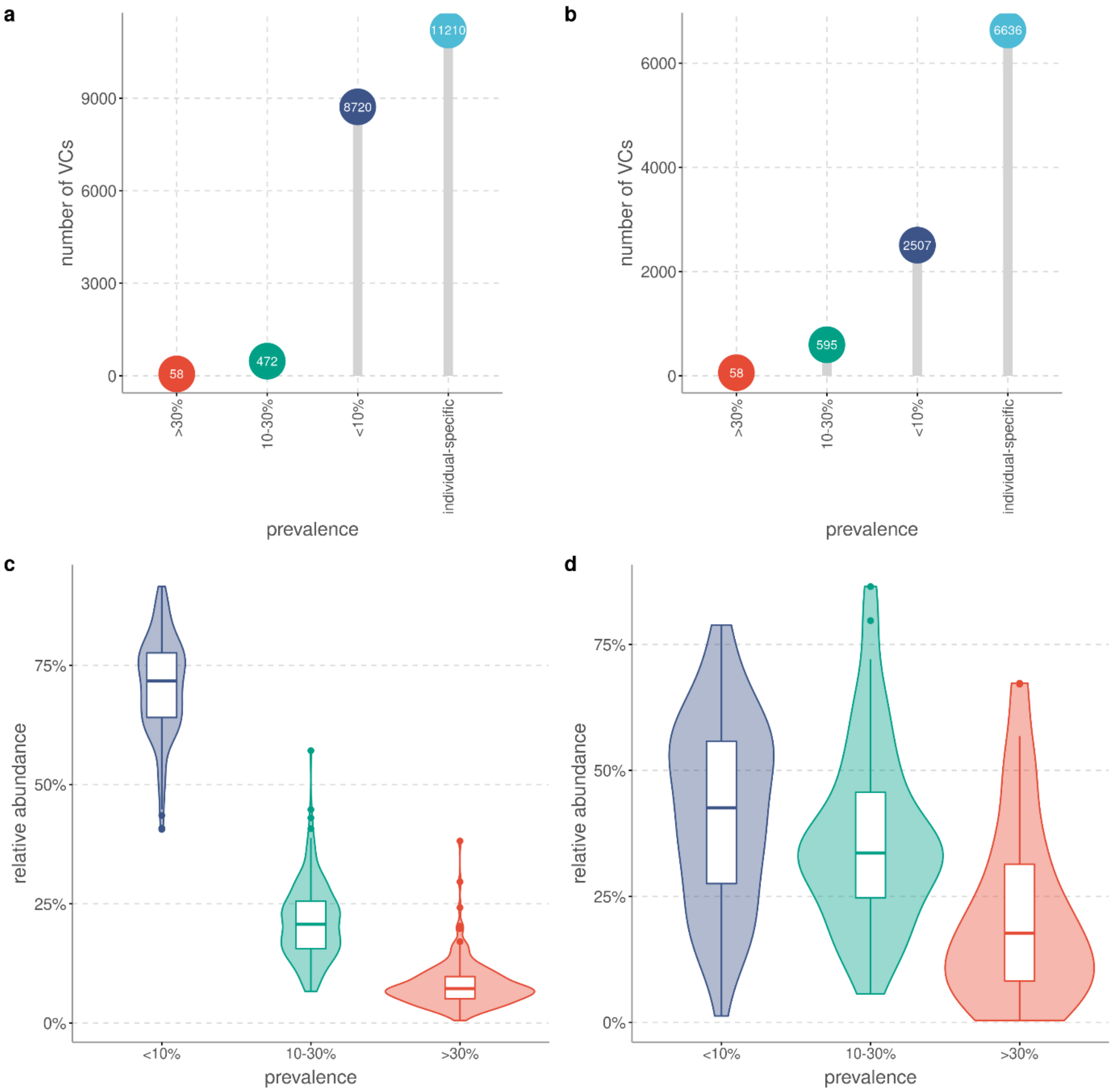

Supplementary Figure 2: Overview of the viromes show high inter-individual variation. **a** Prevalence of VCs among the bulk viromes **b** Prevalence of VCs among the VLP viromes. **c** Total relative abundance of VCs grouped by their prevalence among the participants among bulk viromes. N=196 biologically independent samples. **d** same as c for the VLP viromes. N=48 biologically independent samples. Box plots show the median (middle line), 25th, and 75th percentile (box), with the 25th percentile minus and the 75th percentile plus 1.5 times the interquartile range (whiskers), and outliers (single points).

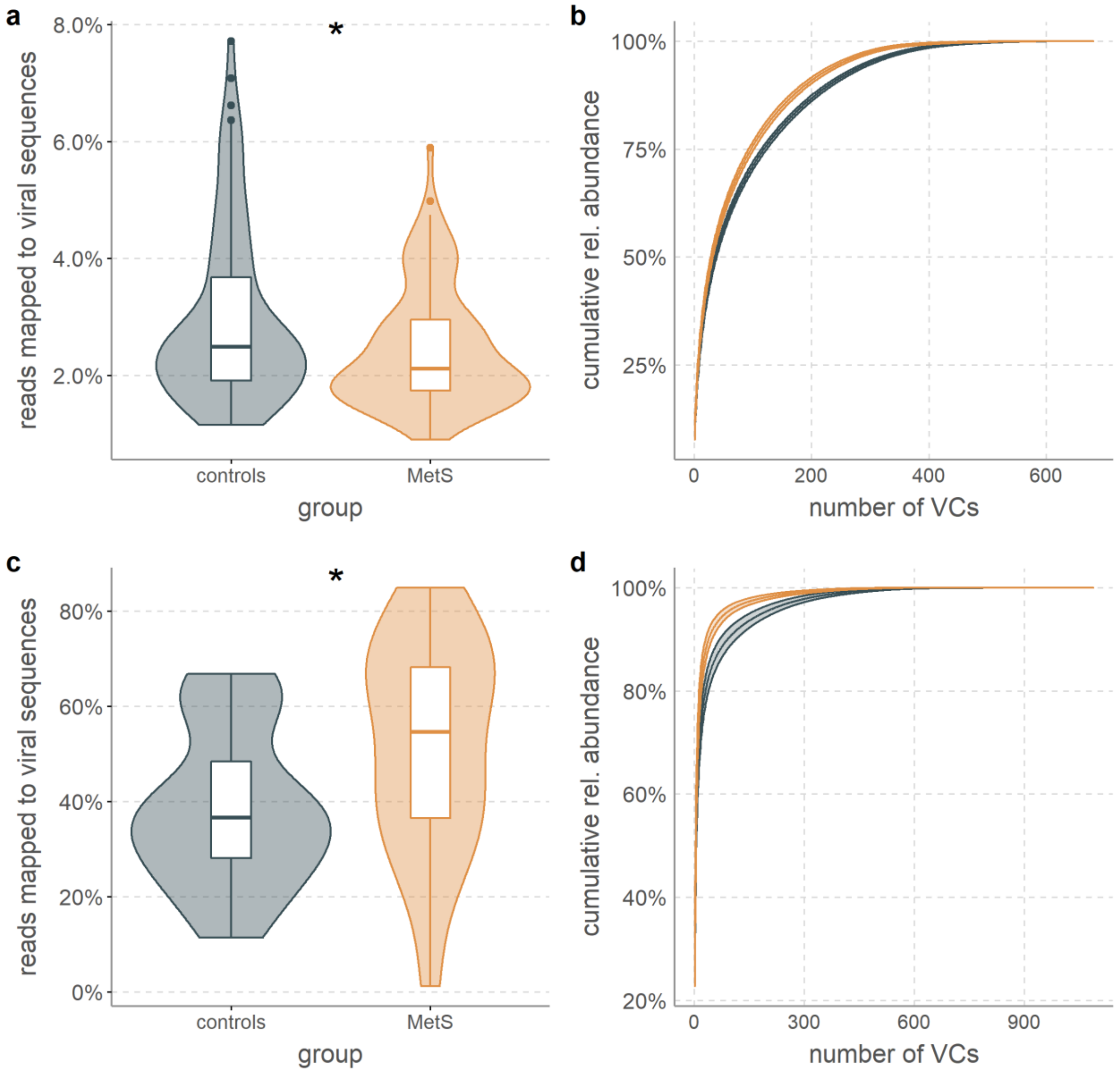

Supplementary Figure 3: Differences in total phage abundance in the two phage populations. a total phage abundance in bulk viromes, as shown by the percentage of reads that map to phage sequences. n=97/n=99 biologically independent samples for MetS and controls, respectively (p = 0.023). **b** cumulative VC ranked-abundance curves of bulk phage samples. MetS is in orange, controls in blue. **c** same as a for VLP viromes. n=24 biologically independent samples for both MetS controls (p = 0.011). **d** same as b for free floating viromes. Stars denote significance according to the two-sided Wilcoxon signed rank test. * ≤ 0.05, ** ≤ 0.01, *** ≤ 0.001, **** ≤ 0.0001. Box plots show the median (middle line), 25th, and 75th percentile (box), with the 25th percentile minus and the 75th percentile plus 1.5 times the interquartile range (whiskers), and outliers (single points).

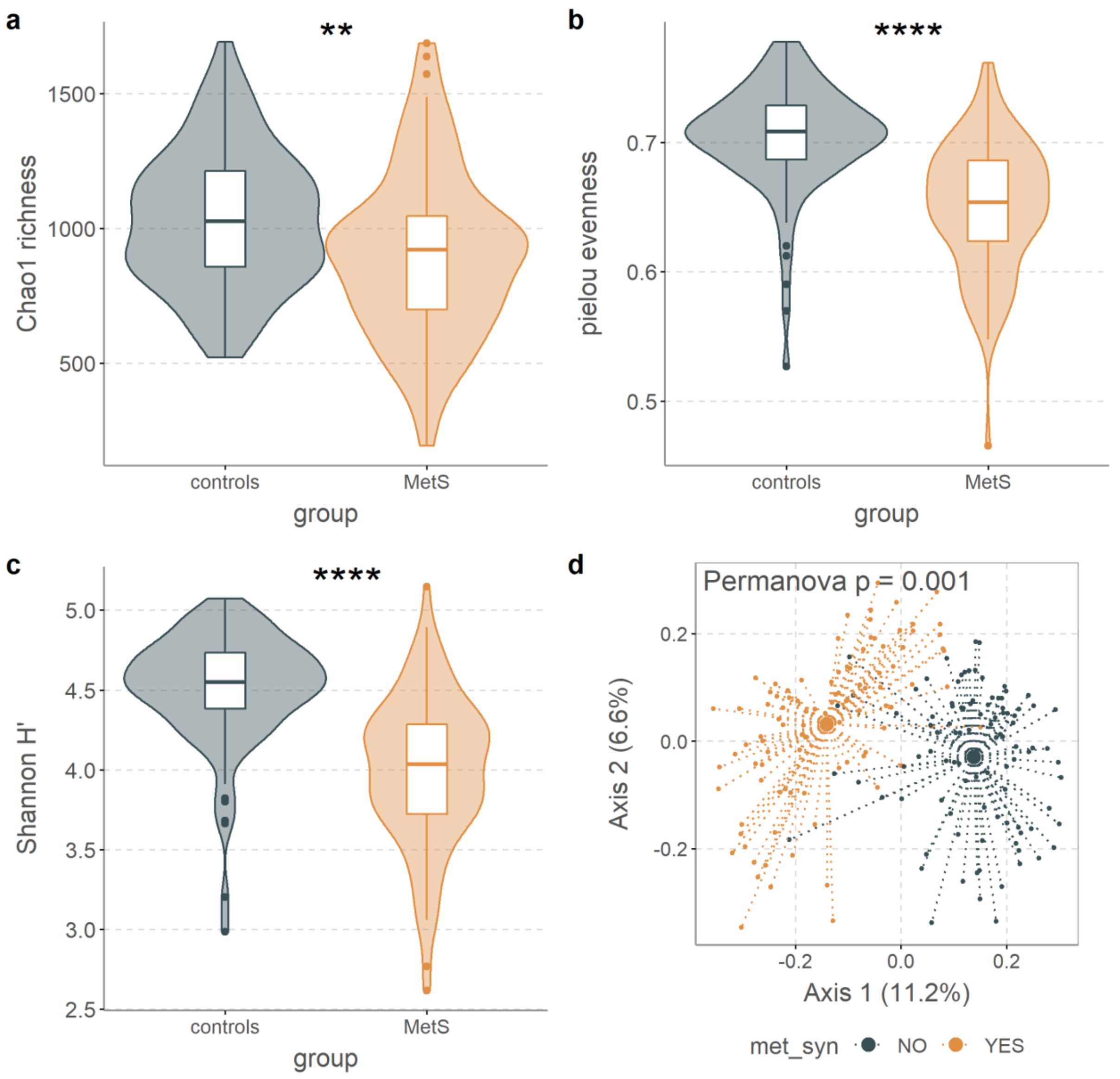

Supplementary Figure 4: Gut bacterium populations are altered in MetS. a MetS-associated decreased bacterial species richness is evidenced by the Chao1 index. n=97/n=99 biologically independent samples for MetS and controls, respectively (p = 9.1 x 10^{-4}). **b** decreased bacterial pielou evenness measurements (p = 1.8 x 10^{-14}). **c** significantly decreased bacterial α-diversity measured by Shannon diversity (p = 1.5 x 10^{-15}). **d** clear separation between bacterial populations of MetS (orange) and control (blue) participant as shown by β-diversity depicted in a principal coordinates analysis (PCoA) of Bray-Curtis dissimilarities. Permanova test was adjusted for smoking, age, sex, alcohol use, and metformin use. Statistical significance in A-C is according to the two-sided Wilcoxon signed rank test, where p-values are denoted as follows: ns not significant, * ≤ 0.05, ** ≤ 0.01, *** ≤ 0.001, **** ≤ 0.0001. Box plots show the median (middle line), 25th, and 75th percentile (box), with the 25th percentile minus and the 75th percentile plus 1.5 times the interquartile range (whiskers), and outliers (single points).

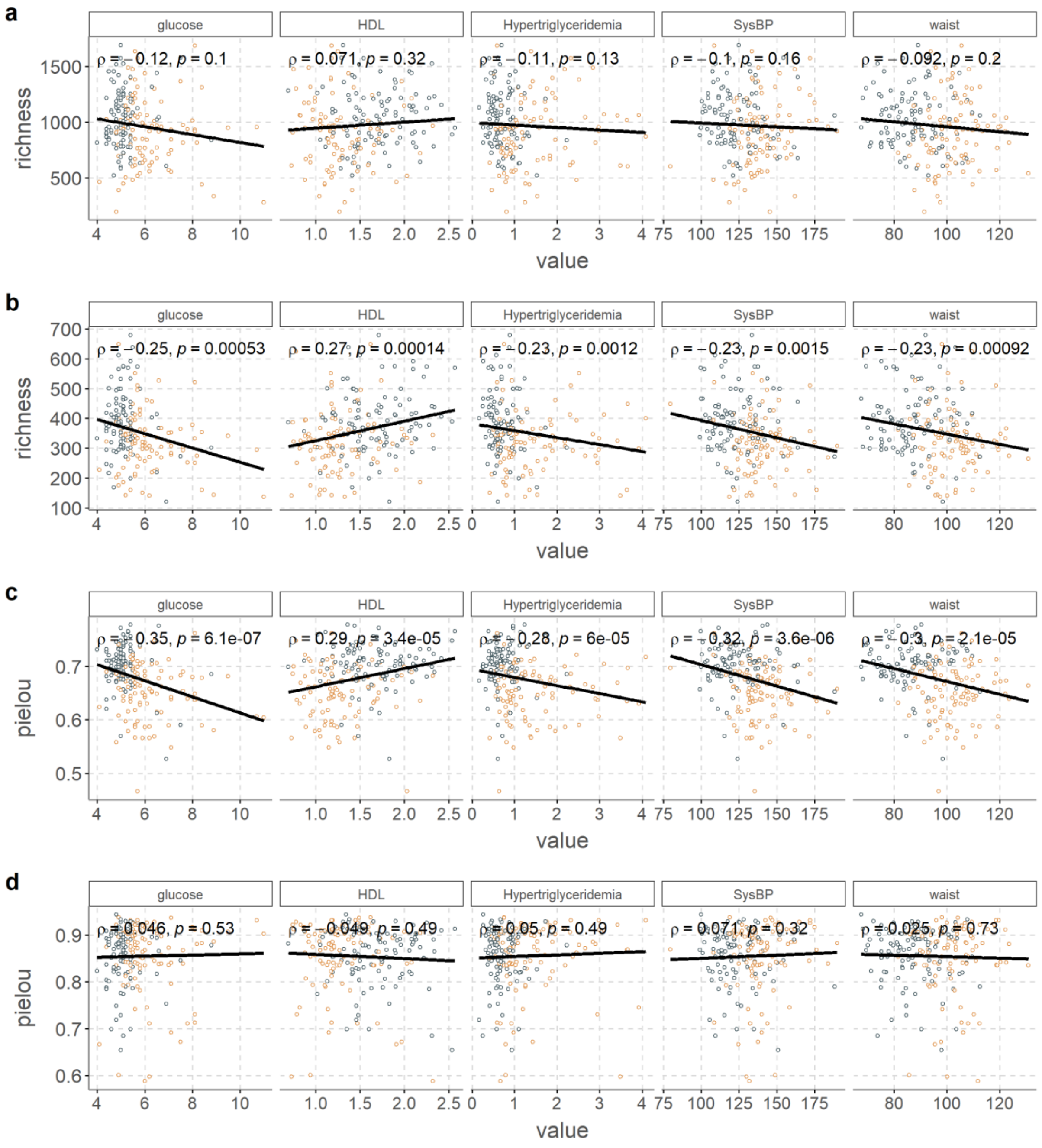

Supplementary Figure 5: Individual correlations between the MetS risk factors and richness of bulk viromes (a) and bacteriomes (b), as well as evenness in viromes (c) and bacteriomes (d). Plotted are the Spearman's rank correlation coefficients. Point colors denote patient group: MetS is in orange, controls in blue.

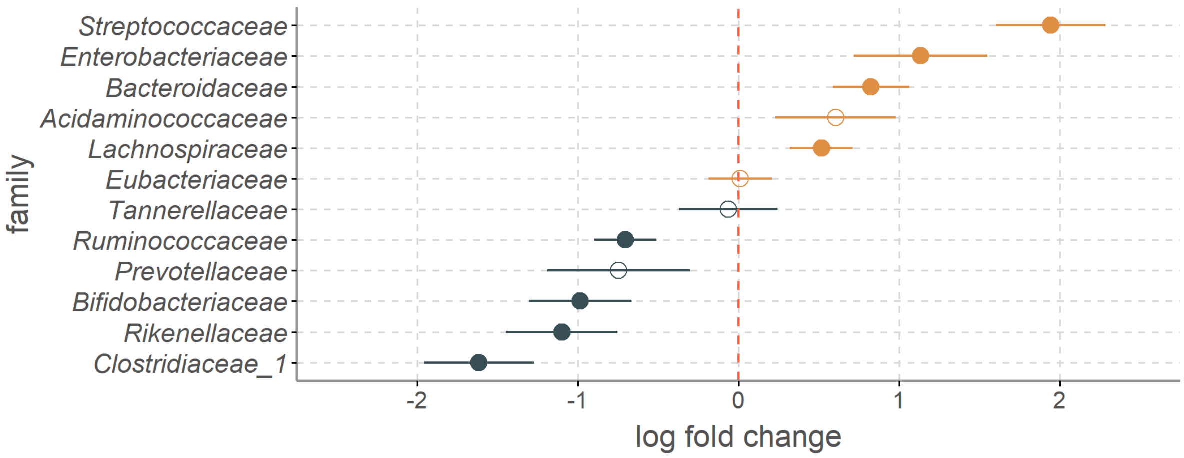

Supplementary Figure 6: ANCOM-BC of bacteria in the top twelve most predated upon bacterial families. Closed circles denote significance, open circles lack of significance. n=97/n=99 biologically independent samples for MetS and controls, respectively. Error bars denote the standard error adjusted by the Benjamini-Hochberg procedure for multiple testing. Color shows in which group the family was most abundant: MetS is in orange, controls in blue.

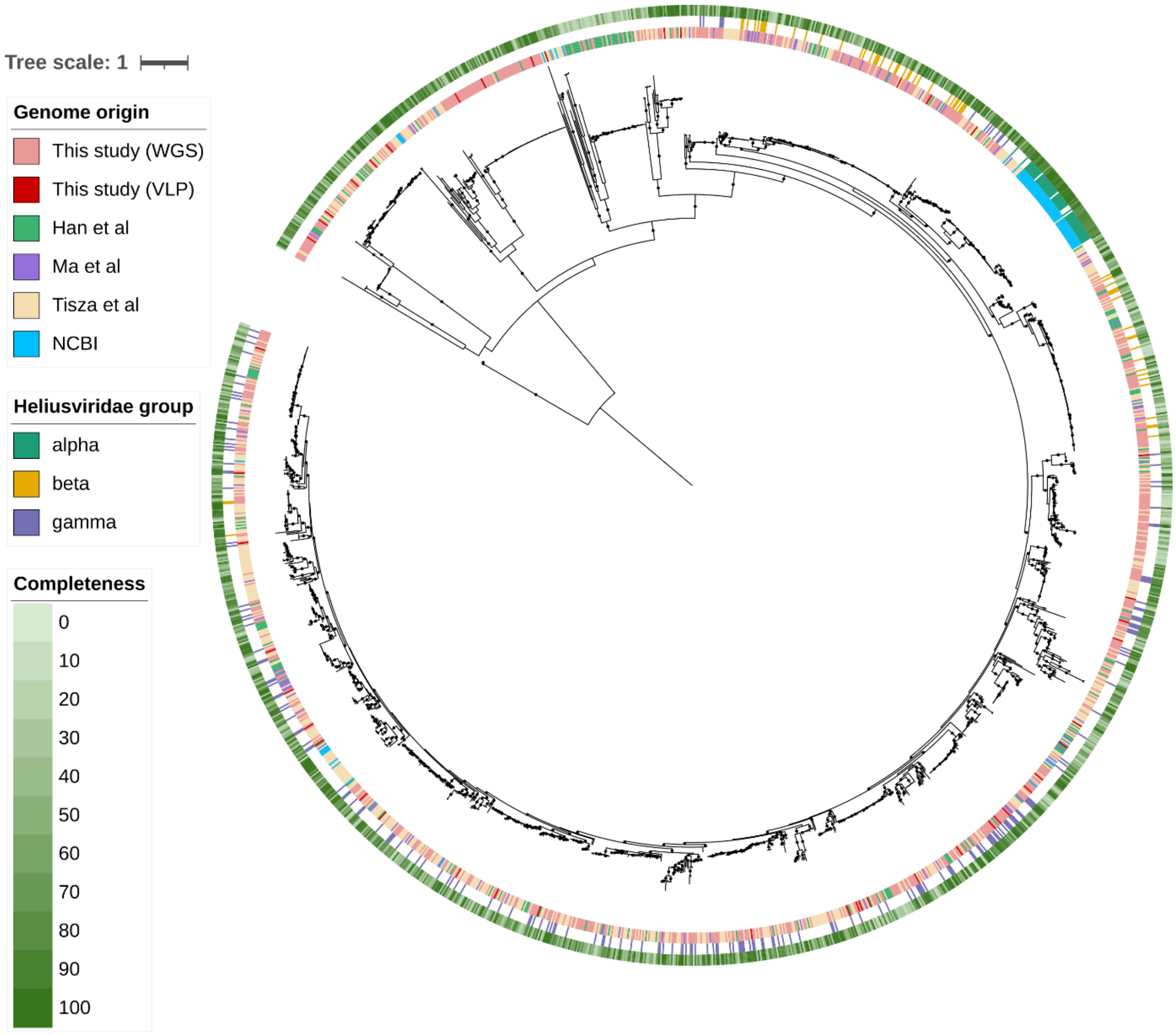

Supplementary Figure 7: A midpoint-rooted approximate maximum likelihood tree made from the concatenated alignments of the four structural *Candidatus Heliusviridae* genes in contigs from this study and multiple cohorts in which the virome was analyzed before. Dots represent bootstrap values of ≥95. The inner ring of colors denotes the study from which the genome was retrieved. The middle ring shows genomes that were assigned to *Ca. Heliusviridae* groups in Figure 5. The outer rich displays genome completeness according to checkV.

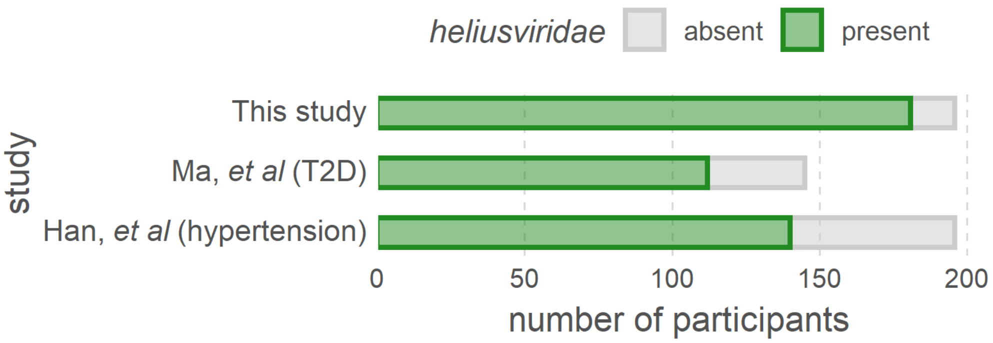

Supplementary Figure 8: Occurrence of *Candidatus Heliusviridae* in this study and two validation cohorts. To circumvent incomplete assemblies, contigs were identified as *Candidatus Heliusviridae* if they 1) contained the terminase, portal protein, major capsid protein, and clp-proteas, and 2) were located in the same clade as *Candidatus Heliusviridae* from this study in the phylogenetic tree depicted in Supplementary Figure 7.

Supplementary Tables

Supplementary Table 1: Characteristics of the Cohort			
	MetS	control	p-value (two-sided Wilcoxon)
Participants	97	99	
Participants with MetS risk factors			
0	0	28	
1	0	34	
2	0	37	
3	35	0	
4	32	0	
5	30	0	
Waist Circumference (cm)	102±9.5	86±10.2	9.2×10^{-07}
Blood Pressure (mmHg)			
Systolic	139.9±16.6	123.4±16.2	1.92×10^{-12}
Diastolic	84.4±10.6	77.6±10.5	1.72×10^{-06}
Blood glucose (mmol/l)	6.2±1.2	5.1±0.6	$< 2 \times 10^{-16}$
HDL (mmol/l)	1.3±0.3	1.7±0.4	1.12×10^{-12}
Triglycerides (mmol/l)	1.5±0.9	0.7±0.3	1.32×10^{-15}
Central obesity	94	58	
High Blood Pressure	91	33	
High Blood Glucose	69	9	
Low HDL	69	5	

High Triglycerides	60	3	

72
