# Supplementary material for: Gut virome profiling identifies a widespread bacteriophage family associated with metabolic syndrome
Source: Nat Commun. 2022 Jun 23;13:3594. doi: 10.1038/s41467-022-31390-5 (PMC9226167; doi:10.1038/s41467-022-31390-5)
Supplement: Supplementary file 2 — Description of Additional Supplementary Files [file 41467_2022_31390_MOESM2_ESM.docx]

**Description of Additional Supplementary Files**

**File Name:** Supplementary Data 1

**Description:** Relative abundance of all VCs across the WGS samples.

**File Name:** Supplementary Data 2

**Description:** VC-host links with taxonomies of the host contigs.

**File Name:** Supplementary Data 3

**Description:** Assignments of viral contigs to VCs.

**File Name:** Supplementary Data 4

**Description:** Annotation of NODE_38_length_205884_cov_102.806990

**File Name:** Supplementary Data 5

**Description:** CheckV results of all viral contigs.

**File Name:** Supplementary Data 6

**Description:** Newick file of the phylogenetic tree in Figure 5b.

**File Name:** Supplementary Data 7

**Description:** Alignment from which the tree in Figure 5b was constructed.

**File Name:** Supplementary Data 8

**Description:** R code of all statistical analysis performed in the study.
