# Supplementary material for: Gut virome profiling identifies a widespread bacteriophage family associated with metabolic syndrome
Source: Nat Commun. 2022 Jun 23;13:3594. doi: 10.1038/s41467-022-31390-5 (PMC9226167; doi:10.1038/s41467-022-31390-5)
Supplement: Supplementary file 10 — Supplementary Dataset 8 [file 41467_2022_31390_MOESM10_ESM.pdf]

Discovery of a highly widespread bacteriophage family and its associations to metabolic syndrome gut microbiomes

P.A. de Jonge, K. Wortelboer, T.P.M. Scheithauer, B-J.H. van den Born, A.H. Zwinderman, F.L. Nobrega, B.E. Dutilh, M. Nieuwdorp, H. Herrema

Last compiled on 05 mei, 2022

preparatory work:

Loading necessary packages

```
source("scripts/R_startup.R")
```

Load the metadata and make a phyloseq sample-data table

```
metadata <- rio::import("datafiles/metadata.txt", header = T) %>%
  mutate(met_syn = factor(met_syn, levels = c("NO", "YES")))

sampledata <- sample_data(data.frame(row.names = metadata$samplename,
                                     met_syn = factor(metadata$met_syn),
                                     ethnicity = factor(metadata$ethnicity),
                                     age = metadata$leeftijd,
                                     sex = factor(metadata$geslacht),
                                     metformin = factor(metadata$metformin),
                                     alcohol = factor(metadata$alcohol),
                                     smoking = factor(metadata$roken)))

options(dplyr.summarise.inform = F)
```

Create color palettes

```
MetS.colors <- get_palette(palette = "jama", k = 2)
names(MetS.colors) <- c("NO", "YES")

BacVir.colors <- get_palette(palette = "aaas", k = 4)[c(3,4)]
names(BacVir.colors) <- c("bacteria", "phages")

category.colors <- get_palette("npg", k = 4)
names(category.colors) <- c(">30", "individual-specific", "10-30", "<10")
```

Make a data frame mapping viral contigs to VCs

```
rio::import("datafiles/VCs.csv") %>%
  filter(startsWith(Genome, "NODE")) %>%
  transmute(contigName = Genome,
            VC_Subcluster = ifelse(startsWith(`VC Status`, "Overlap"), `VC Status`,
                                   ifelse(`VC Subcluster` == "", contigName,
                                          `VC Subcluster`)),
            VC_Status = ifelse(startsWith(VC_Subcluster, "NODE"),
                               "unclustered",
                               "clustered"),
            contigName = gsub("_S.+$", "", contigName)) %>%
  group_by(contigName) %>%
  mutate(contigs = n(),
         contigName = ifelse(contigs > 1,
                             paste(contigName, row_number(), sep = "_"),
                             contigName),
         VC_Subcluster = ifelse(startsWith(VC_Subcluster, "NODE"),
                                contigName,
                                VC_Subcluster)) %>%
  ungroup() %>%
  select(-contigs) -> VCs
```

Read table with the raw read counts per contig per sample and table with the total number of reads per sample. Raw read count table has been filtered for contigs with >75% coverage with reads mapped at >90% identity

```
raw_reads <- tibble(rio::import("datafiles/raw_read_counts.csv"))

total_reads <- tibble(rio::import("datafiles/total_reads.csv"))
```

data preparation:

A function that calculates an RPKM table and creates three phyloseq objects: one with RPKM values, one compositional, and one with raw read counts.

```
ps.objects <- function(dataset, analysis){

  raw_reads %>%
    filter(datatype == analysis) %>%
    inner_join(VCs[,-3], by = "contigName") %>%
    group_by(samplename) %>%
    mutate(M = sum(mapped)/1e6) %>%
    ungroup() %>%
    group_by(samplename, VC_Subcluster) %>%
    summarise(RPKM = sum(mapped)/(sum(contigLen/1000)*M)) %>%
    ungroup() %>%
    distinct() -> RPKM_table

  RPKM_table %>%
    select(samplename) %>%
    distinct() %>%
    left_join(metadata[,c(10,2:9)], by = "samplename") %>%
    column_to_rownames("samplename") %>%
```

```
    sample_data() -> sample_data

  ################################################################################

  RPKM_table %>%
    pivot_wider(names_from = samplename, values_from = RPKM, values_fill = 0) %>%
    column_to_rownames("VC_Subcluster") %>%
    otu_table(taxa_are_rows = T) -> otu_table.relative

  relative.ps <- phyloseq(otu_table.relative, sample_data)
  relative.ps <- transform(relative.ps, "compositional")

  assign(paste("relative.", dataset, ".ps", sep = ""), relative.ps, envir = globalenv())

  ################################################################################

  RPKM_table %>%
    pivot_wider(names_from = samplename, values_from = RPKM, values_fill = 0) %>%
    column_to_rownames("VC_Subcluster") %>%
    otu_table(taxa_are_rows = T) -> otu_table.RPKM

  RPKM.ps <- phyloseq(otu_table.RPKM, sample_data)

  assign(paste("RPKM.", dataset, ".ps", sep = ""), RPKM.ps, envir = globalenv())

  ################################################################################

  raw_reads %>%
    filter(datatype == analysis) %>%
    inner_join(VCs[,-3], by = "contigName")  %>%
    group_by(VC_Subcluster, samplename) %>%
    summarise(mapped = sum(mapped)) %>%
    ungroup() %>%
    pivot_wider(names_from = samplename, values_from = mapped, values_fill = 0) %>%
    column_to_rownames("VC_Subcluster") %>%
    otu_table(taxa_are_rows = T) -> otu_table.counts

  counts.ps <- phyloseq(otu_table.counts, sample_data)

  assign(paste("counts.", dataset, ".ps", sep = ""), counts.ps, envir = globalenv())

}
```

create phyloseq object for bulk viral data

```
ps.objects("bulk", "WGS")

relative.bulk.ps
```

```
## phyloseq-class experiment-level object
## otu_table()   OTU Table:         [ 20460 taxa and 196 samples ]
## sample_data() Sample Data:       [ 196 samples by 8 sample variables ]
```

```
RPKM.bulk.ps
```

```
## phyloseq-class experiment-level object
## otu_table()   OTU Table:         [ 20460 taxa and 196 samples ]
## sample_data() Sample Data:       [ 196 samples by 8 sample variables ]
```

```
counts.bulk.ps
```

```
## phyloseq-class experiment-level object
## otu_table()   OTU Table:         [ 20460 taxa and 196 samples ]
## sample_data() Sample Data:       [ 196 samples by 8 sample variables ]
```

create phyloseq object for VLP viral data

```
ps.objects("VLP", "VLP")

relative.VLP.ps
```

```
## phyloseq-class experiment-level object
## otu_table()   OTU Table:         [ 9796 taxa and 48 samples ]
## sample_data() Sample Data:       [ 48 samples by 8 sample variables ]
```

```
RPKM.VLP.ps
```

```
## phyloseq-class experiment-level object
## otu_table()   OTU Table:         [ 9796 taxa and 48 samples ]
## sample_data() Sample Data:       [ 48 samples by 8 sample variables ]
```

```
counts.VLP.ps
```

```
## phyloseq-class experiment-level object
## otu_table()   OTU Table:         [ 9796 taxa and 48 samples ]
## sample_data() Sample Data:       [ 48 samples by 8 sample variables ]
```

loading of bacterial phyloseq objects

```
raw.16s.ps <- readRDS("datafiles/ps.helius_metsyn_subset.RDS")

sample_data.16s <- metadata[,-10] %>%
  column_to_rownames("heliusnr")

raw.16s.ps <- phyloseq(otu_table(raw.16s.ps),
                       tax_table(raw.16s.ps),
                       phy_tree(raw.16s.ps),
                       sample_data(sample_data.16s))

raw.16s.ps <- prune_taxa(taxa_sums(raw.16s.ps) > 0, raw.16s.ps)
relative.16s.ps <- transform(raw.16s.ps,"compositional")
```

Figure 1:

General function for all box plots below, with a Wilcox signed rank test

```
MetS_violin_box <- function(data, y, ylab,  comparisons = NULL){
  ggviolin(data = data,
           x = "met_syn",
           y = y,
           color = "met_syn",
           fill = "met_syn",
           palette = MetS.colors,
           alpha = 0.4,
           add = "boxplot",
           add.params = list(fill = "white"),
           ggtheme = PAdJ_theme(),
           ylab = ylab,
           xlab = "group",
           trim = T) +
  stat_compare_means(vjust = 0.75,
                     label = "p.signif",
                     label.x.npc = "center",
                     comparisons = comparisons,
                     method = "wilcox.test") +
  theme(legend.position = "",
        aspect.ratio = 1,
        panel.grid.major.x = element_blank()) +
  scale_x_discrete(labels = c("controls", "MetS"))
}
```

Figure 1A: bulk richness

```
richness(x = RPKM.bulk.ps, "observed") %>%
  rownames_to_column("samplename") %>%
  left_join(metadata, by = "samplename") %>%
  MetS_violin_box(y = "observed", ylab = "observed VCs (n)")
```

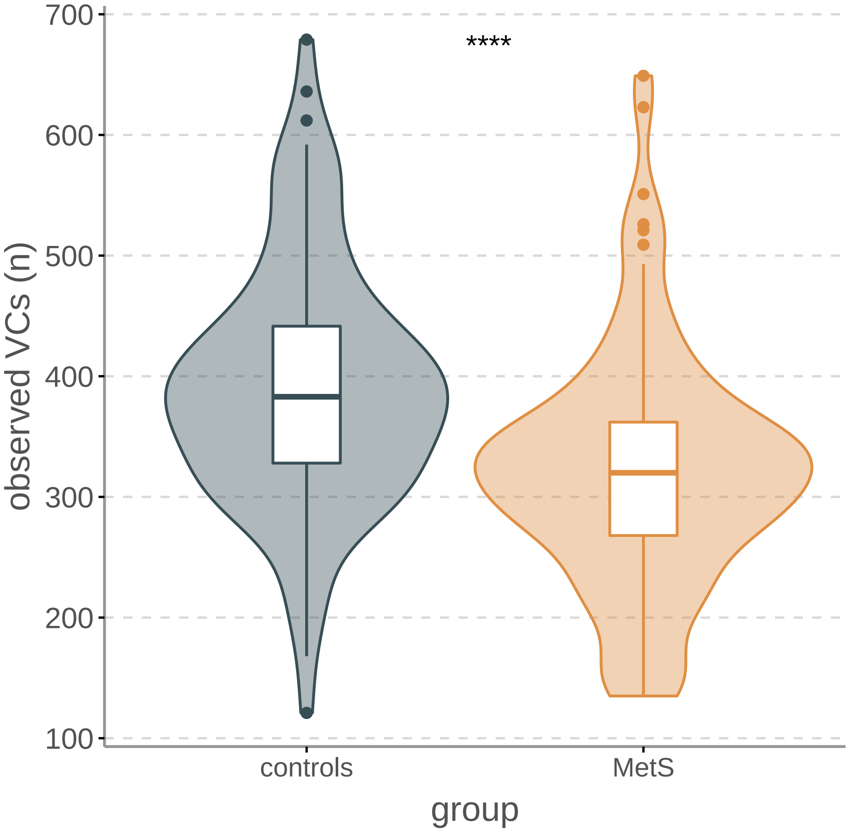

Figure 1B: bulk evenness

```
evenness(x = RPKM.bulk.ps, "pielou") %>%
  rownames_to_column("samplename") %>%
  left_join(metadata, by = "samplename") %>%
  MetS_violin_box(y = "pielou", ylab = "pielou evenness")
```

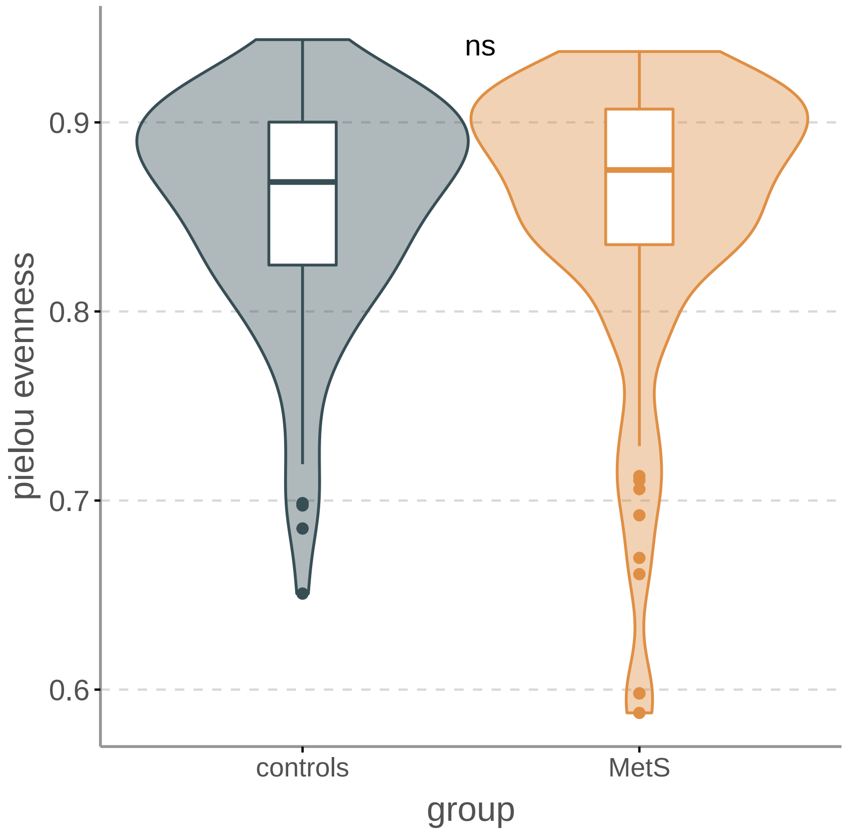

Figure 1C: bulk alpha diversity

```
diversity(x = RPKM.bulk.ps, "shannon") %>%
  rownames_to_column("samplename") %>%
  left_join(metadata, by = "samplename") %>%
  MetS_violin_box(y = "shannon", ylab = "Shannon H'")
```

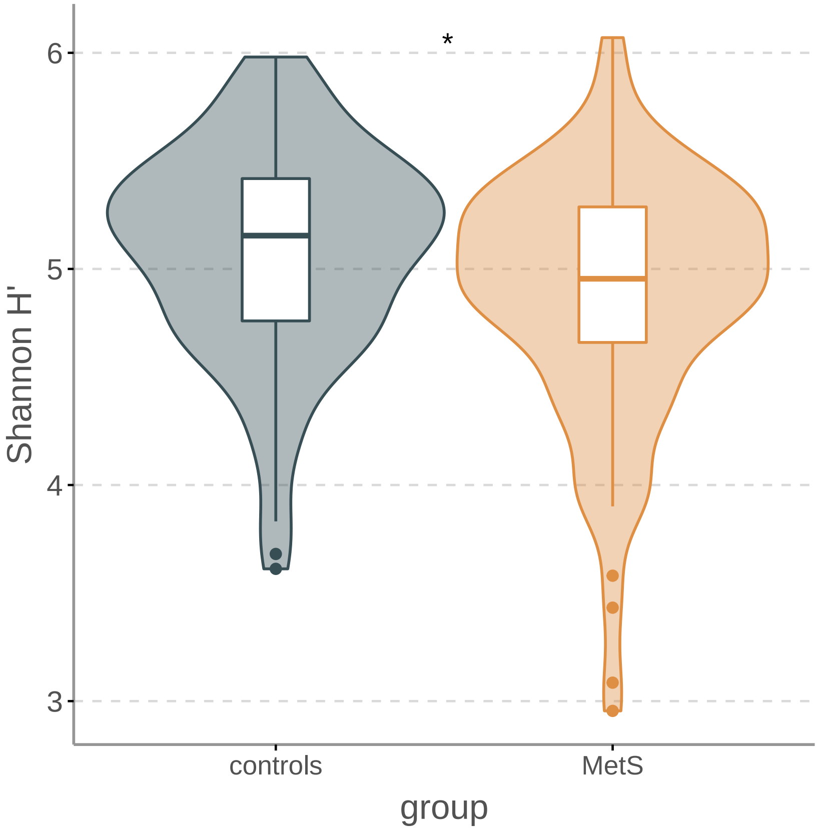

bulk beta diversity — ***PERMANOVA***

```
BC <- phyloseq::distance(relative.bulk.ps, method = "bray")
set.seed(130499)
adonis2(BC ~ roken + geslacht + leeftijd + alcohol + metformin + met_syn,
       data = data.frame(relative.bulk.ps@sam_data))
```

```
## Permutation test for adonis under reduced model
## Terms added sequentially (first to last)
## Permutation: free
## Number of permutations: 999
##
## adonis2(formula = BC ~ roken + geslacht + leeftijd + alcohol + metformin + met_syn, data = data.frame
##             Df SumOfSqs      R2      F Pr(>F)
## roken        1    0.493 0.00554 1.0956  0.046 *
## geslacht     1    0.546 0.00614 1.2141  0.004 **
## leeftijd     1    0.557 0.00627 1.2400  0.001 ***
## alcohol      1    0.524 0.00589 1.1648  0.006 **
## metformin    1    0.675 0.00759 1.5016  0.001 ***
## met_syn      1    1.175 0.01322 2.6145  0.001 ***
## Residual   189   84.963 0.95536
## Total      195   88.933 1.00000
## ---
## Signif. codes:  0 '***' 0.001 '**' 0.01 '*' 0.05 '.' 0.1 ' ' 1
```

```
set.seed(130499)
adonis_out <- adonis2(BC ~ roken + geslacht + leeftijd + alcohol + metformin + met_syn,
       data = data.frame(relative.bulk.ps@sam_data))
```

Figure 1D: bulk beta diversity — *PCoA*

```
PCoA.bulk <- ordinate(relative.bulk.ps, method = "PCoA", distance = "bray")

eigen <- PCoA.bulk$values[c(1,2),2]

as.data.frame(PCoA.bulk$vectors[,c(1,2)]) %>%
  rownames_to_column("samplename") %>%
  left_join(metadata, by = "samplename") %>%
  ggscatter(x = "Axis.1",
            y = "Axis.2",
            color = "met_syn",
            palette = MetS.colors,
            star.plot = T,
            star.plot.lty = "dotted",
            mean.point = T,
            mean.point.size = 3,
            ggtheme = PAdJ_theme(),
            size = .75) +
  theme(aspect.ratio = 1,
        panel.border = element_rect(color = "#969696"),
        axis.text.y = element_text(size=10)) +
  annotate("text",
           label = paste("Permanova p =", adonis_out$`Pr(>F)`[6]),
           x = -0.12,
           y = 0.3,
           color = "grey30",
           size = 5) +
  scale_x_continuous(name = paste("Axis 1 (", round(eigen[1]*100, 1), "%)", sep = "")) +
  scale_y_continuous(name = paste("Axis 2 (", round(eigen[2]*100, 1), "%)", sep = ""))
```

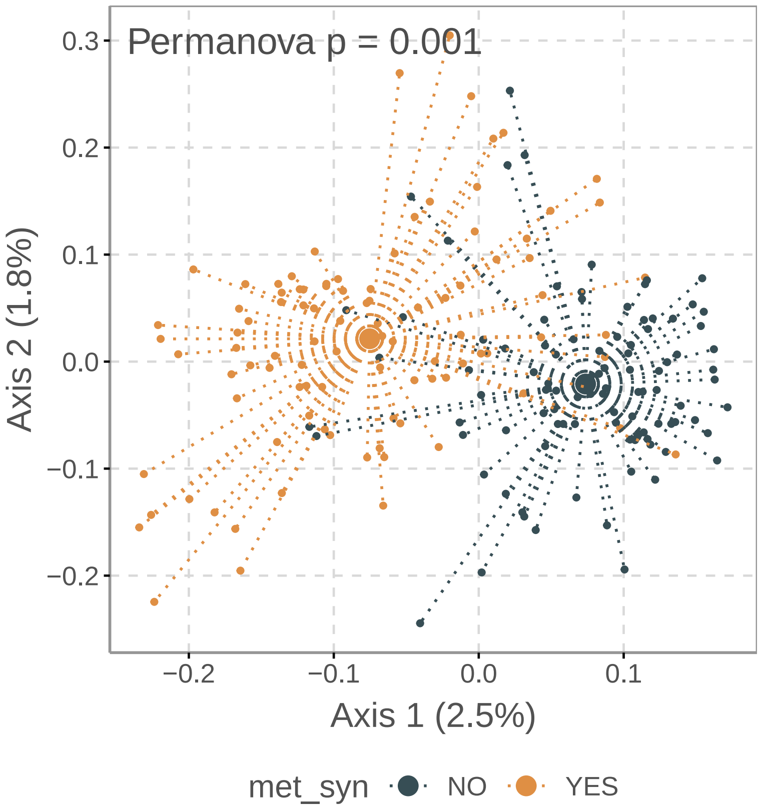

Figure 1E: VLP richness

```
richness(x = RPKM.VLP.ps, "observed") %>%
  rownames_to_column("samplename") %>%
  left_join(metadata, by = "samplename") %>%
  MetS_violin_box(y = "observed", ylab = "observed VCs (n)")
```

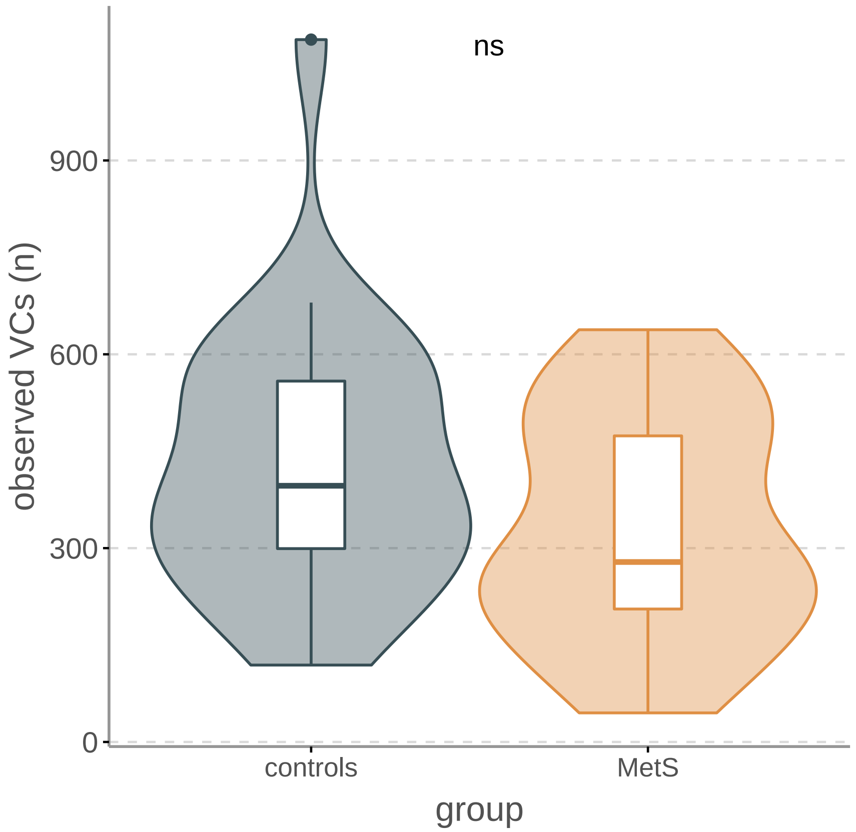

Figure 1F: VLP evenness

```
evenness(x = RPKM.VLP.ps, "pielou") %>%
  rownames_to_column("samplename") %>%
  left_join(metadata, by = "samplename") %>%
  MetS_violin_box(y = "pielou", ylab = "pielou evenness")
```

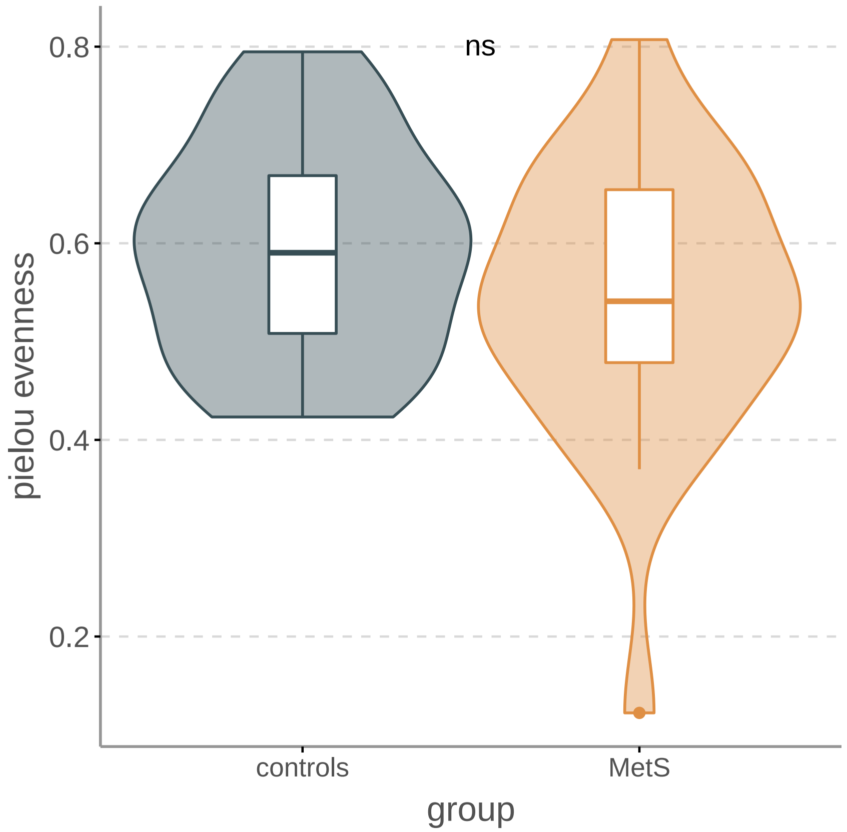

Figure 1G: VLP alpha diversity

```
diversity(x = RPKM.VLP.ps, "shannon") %>%
  rownames_to_column("samplename") %>%
  left_join(metadata, by = "samplename") %>%
  MetS_violin_box(y = "shannon", ylab = "Shannon H'")
```

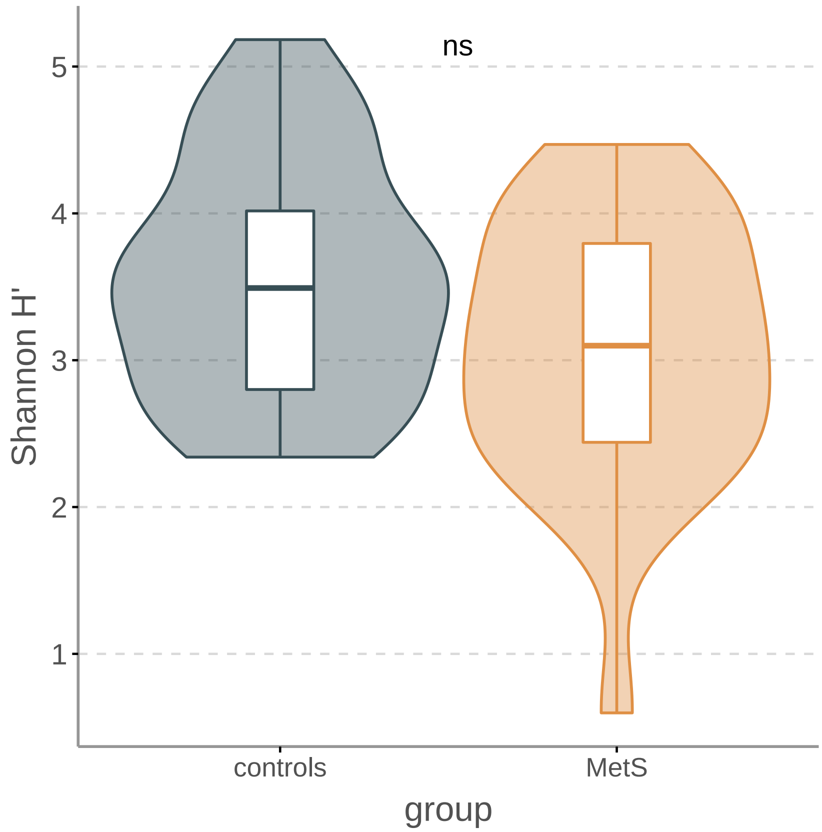

VLP beta diversity — ***PERMANOVA***

```
BC <- phyloseq::distance(relative.VLP.ps, method = "bray")
set.seed(130499)
adonis2(BC ~ met_syn,
       data = data.frame(relative.VLP.ps@sam_data))
```

```
## Permutation test for adonis under reduced model
## Terms added sequentially (first to last)
## Permutation: free
## Number of permutations: 999
##
## adonis2(formula = BC ~ met_syn, data = data.frame(relative.VLP.ps@sam_data))
##          Df SumOfSqs      R2      F Pr(>F)
## met_syn   1   0.5393 0.02404 1.1332  0.039 *
## Residual 46  21.8935 0.97596
## Total    47  22.4329 1.00000
## ---
## Signif. codes:  0 '***' 0.001 '**' 0.01 '*' 0.05 '.' 0.1 ' ' 1
```

```
set.seed(130499)
adonis_out <- adonis2(BC ~ met_syn,
       data = data.frame(relative.VLP.ps@sam_data))
```

Figure 1H: VLP beta diversity — *PCoA*

```
PCoA <- ordinate(relative.VLP.ps, method = "PCoA", distance = "bray")

eigen <- PCoA$values[c(1,2),2]

as.data.frame(PCoA$vectors[,c(1,2)]) %>%
  rownames_to_column("samplename") %>%
  left_join(metadata, by = "samplename") %>%
  ggscatter(x = "Axis.1",
            y = "Axis.2",
            color = "met_syn",
            palette = MetS.colors,
            star.plot = T,
            star.plot.lty = "dotted",
            mean.point = T,
            mean.point.size = 3,
            ggtheme = PAdJ_theme(),
            size = .75) +
  theme(aspect.ratio = 1,
        panel.border = element_rect(color = "#969696"),
        axis.text.y = element_text(size=10)) +
  annotate("text",
           label = paste("Permanova p =", adonis_out$`Pr(>F)`[1]),
           x = 0.05,
           y = 0.4,
           color = "grey30",
           size = 5) +
  scale_x_continuous(name = paste("Axis 1 (", round(eigen[1]*100, 1), "%)", sep = "")) +
  scale_y_continuous(name = paste("Axis 2 (", round(eigen[2]*100, 1), "%)", sep = ""))
```

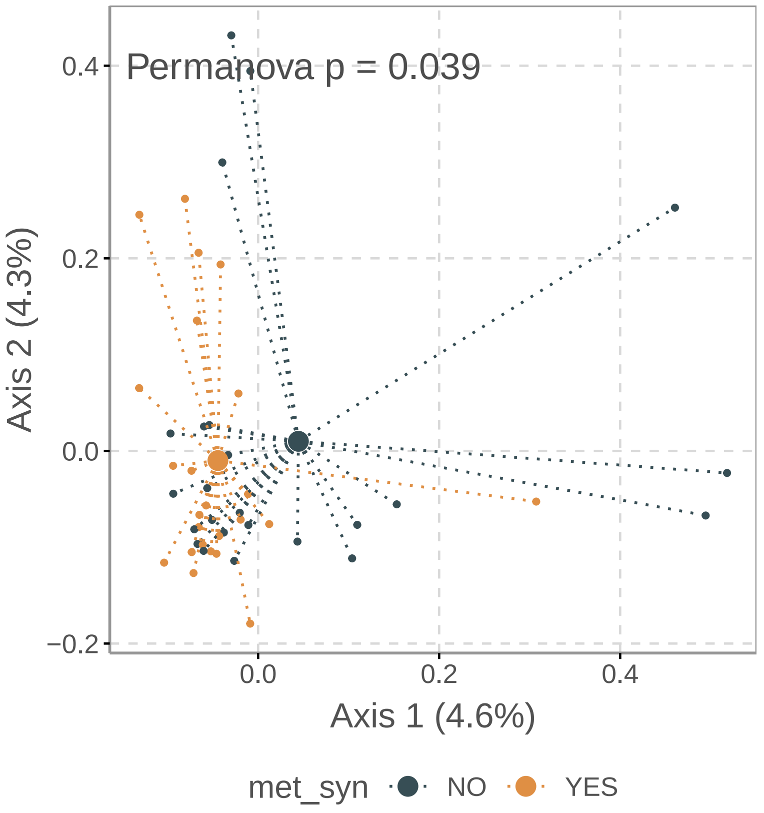

Figure 2: Correlations between bulk viromes and bacteria/clinical MetS parameters

Load clinical data

```
clinical_data <- rio::import("datafiles/metadata_wide.csv")
```

Calculate the correlations between richness and evenness in phages and bacteria and clinical parameters.

```
richness_evenness <- function(input, prefix, datatype){

  if(datatype == "Viral"){
    richness(x = input, "observed") %>%
      rownames_to_column("samplename") %>%
      dplyr::rename(richness = observed) %>%
      left_join(metadata[,c(10,8)], by = "samplename") -> output_richness

      evenness(x = input, "pielou") %>%
      rownames_to_column("samplename") %>%
      left_join(metadata[,c(10,8)], by = "samplename") -> output_evenness
  } else{
    richness(x = input, "Chao1") %>%
      rownames_to_column("samplename") %>%
```

```
      mutate(samplename = paste("S", samplename, sep = "")) %>%
      dplyr::rename(richness = chao1) %>%
      left_join(metadata[,c(10,8)], by = "samplename") -> output_richness

    evenness(x = input, "pielou") %>%
      rownames_to_column("samplename") %>%
      mutate(samplename = paste("S", samplename, sep = "")) %>%
      left_join(metadata[,c(10,8)], by = "samplename") -> output_evenness
  }

  left_join(clinical_data, output_richness, by = "samplename") %>%
      left_join(output_evenness, by = c("samplename", "met_syn")) -> data

  with(data,
       lapply(list(richness, pielou),
              function(x) lapply(list(glucose, waist, Hypertriglyceridemia, HDL, SysBP),
                                 x = x,
                                 cor.test,
                                 method = "spearman", exact = F))) -> cor_list

  do.call(rbind, lapply(list(1,2,3,4,5),
                        function(i) broom::tidy(cor_list[[1]][[i]]))) %>%
    rbind(do.call(rbind, lapply(list(1,2,3,4,5),
                                function(i) broom::tidy(cor_list[[2]][[i]])))) %>%
    select(-method, -alternative, -statistic) %>%
    mutate(clinical = rep(c("glucose", "obesity", "triglycerides", "HDL", "BP"), 2),
           ecological = c(rep("richness", 5), rep("evenness",5))) -> correlations

  assign(paste(prefix, datatype, "richness", sep = "_"),
         output_richness, envir = globalenv())

  assign(paste(prefix, datatype, "evenness", sep = "_"),
         output_evenness, envir = globalenv())

  assign(paste(prefix, datatype, "correlations", sep = "_"),
         correlations, envir = globalenv())

}
```

General function for the scatter plots, with a Spearman's rack correlation coeffiecient

```
MetS_scatter <- function(data, x, y, xlab, ylab) {
  ggscatter(data = data,
            x = x,
            y = y,
            add = "reg.line",
            add.params = list(color = "black",
                              size = 1),
            size = 0.75,
            color = "met_syn",
            shape = 21,
            alpha = 0.9,
            xlab = xlab,
```

```
             ylab = ylab,
             cor.coef = T,
             cor.coeff.args = list(method = "spearman",
                                   cor.coef.name = "rho",
                                   size = 3.5),
             ggtheme = PAdJ_theme(),
             palette = MetS.colors) +
  theme(aspect.ratio = 1,
        legend.position = "")
}
```

Run correlation function for the viral and bacterial datasets

```
richness_evenness(input = RPKM.bulk.ps, prefix = "WGS", datatype = "Viral")
richness_evenness(input = raw.16s.ps, prefix = "WGS", datatype = "Bacterial")
```

Figure 2A: correlation between bacterial/phage richness

```
WGS_Bacterial_richness %>%
  transmute(samplename = samplename,
            bacteria = richness,
            met_syn = met_syn) %>%
  inner_join(WGS_Viral_richness, by = c("samplename", "met_syn")) %>%
  MetS_scatter(x = "richness", y = "bacteria",  xlab = "intracellular phage richness",
               ylab = "bacterial richness")
```

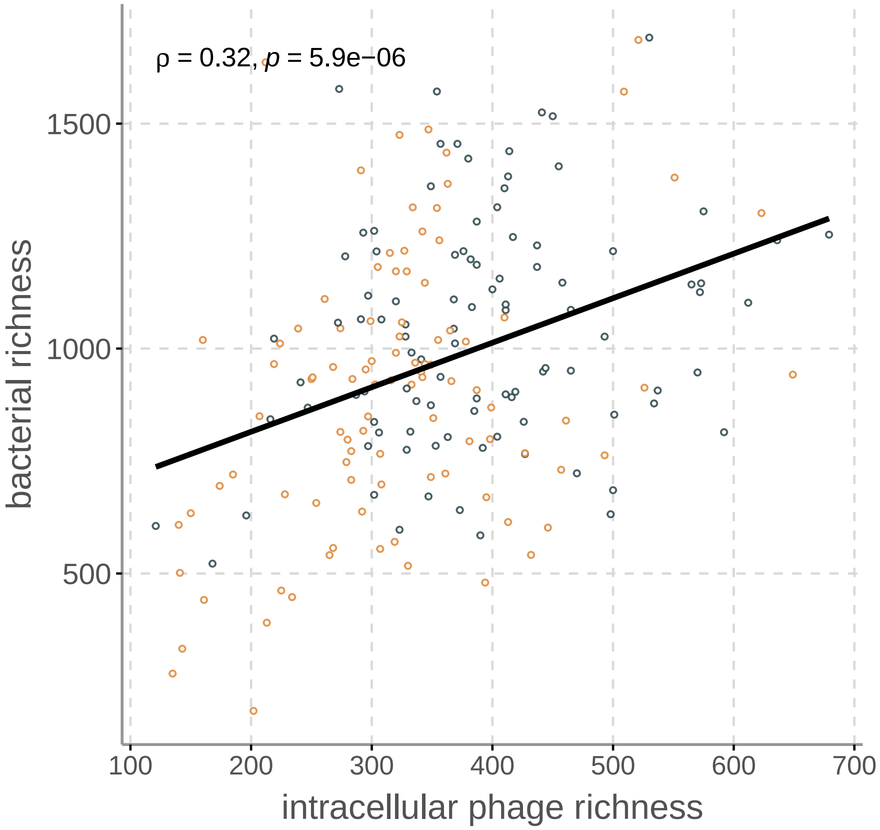

Figure 2B: correlation of evenness

```
WGS_Bacterial_evenness %>%
  transmute(samplename = samplename,
            bacteria = pielou,
            met_syn = met_syn) %>%
  inner_join(WGS_Viral_evenness, by = c("samplename", "met_syn")) %>%
  MetS_scatter(x = "pielou", y = "bacteria",  xlab = "intracellular phage evenness",
               ylab = "bacterial evenness")
```

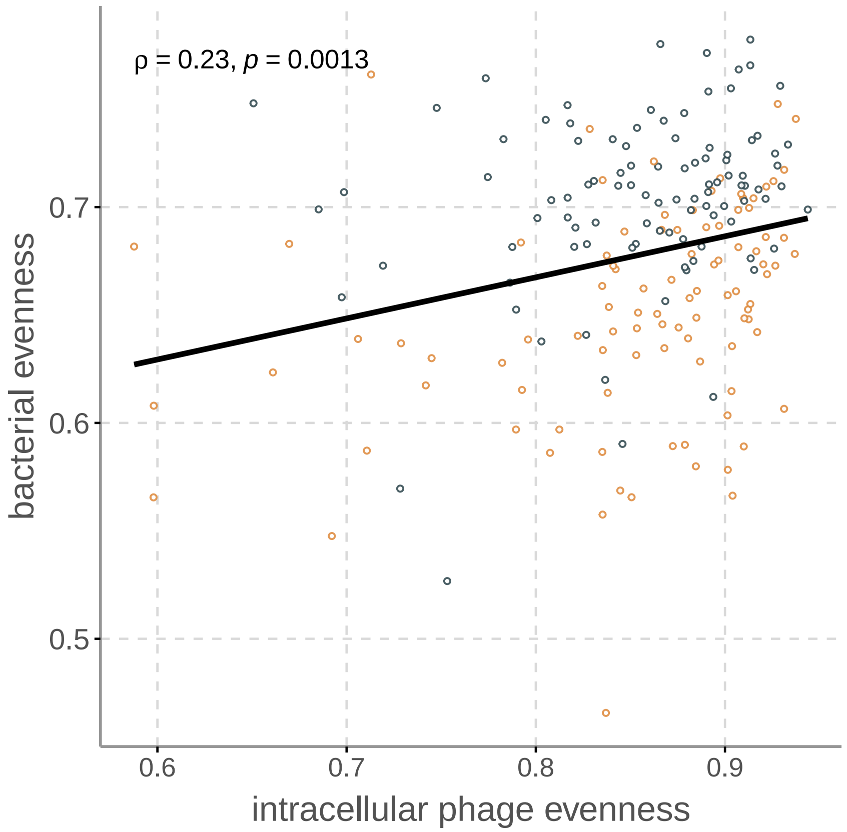

Figure 2C: correlation of richness vs clinical parameters

```
WGS_Viral_correlations %>%
  rbind(WGS_Bacterial_correlations) %>%
  mutate(population = c(rep("phages", 10), rep("bacteria", 10)),
         q = p.adjust(p.value, "BH")) %>%
  filter(ecological == "richness") %>%
  ggdotchart(x = "population",
             y = "estimate",
             color = "population",
             palette = BacVir.colors,
             ggtheme = PAdJ_theme(),
             dot.size = 3,
             ylab = expression("spearman"~rho),
             rotate = T) +
  theme(legend.position = "",
        panel.grid.major.x = element_blank(),
        strip.background = element_rect(fill = "white")) +
  geom_hline(yintercept = 0,
             color = "tomato1",
             linetype = "dashed",
             size = 0.5) +
  gghighlight::gghighlight(q <= 0.05,
                           label_key = clinical,
```

```
                              unhighlighted_params = list(colour = NULL, shape = 21))
```

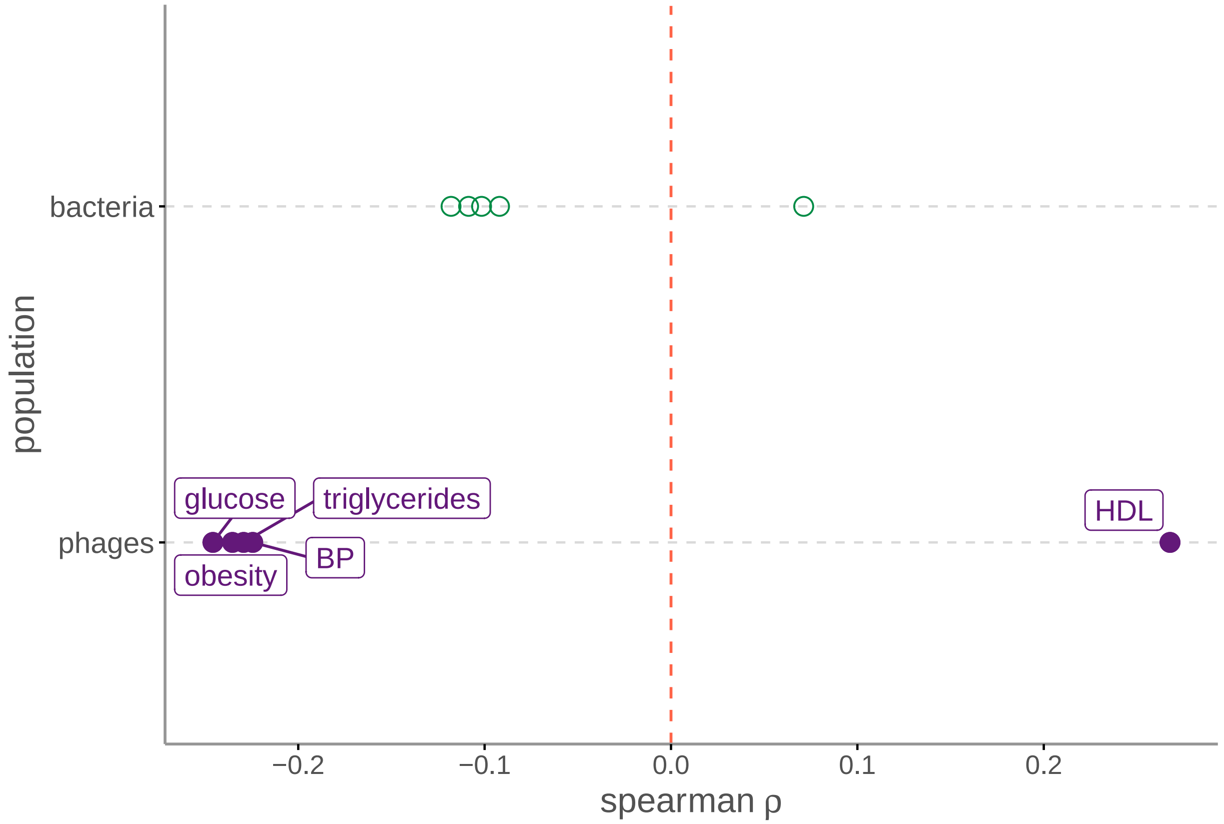

Figure 2D: correlation of evenness vs clinical parameters

```
WGS_Viral_correlations %>%
  rbind(WGS_Bacterial_correlations) %>%
  mutate(population = c(rep("phages", 10), rep("bacteria", 10)),
         q = p.adjust(p.value, "BH")) %>%
  filter(ecological == "evenness") %>%
  ggdotchart(x = "population",
             y = "estimate",
             color = "population",
             palette = BacVir.colors,
             ggtheme = PAdJ_theme(),
             dot.size = 3,
             ylab = expression("spearman"~rho),
             rotate = T) +
  theme(legend.position = "",
        panel.grid.major.x = element_blank(),
        strip.background = element_rect(fill = "white")) +
  geom_hline(yintercept = 0,
             color = "tomato1",
             linetype = "dashed",
```

```
              size = 0.5) +
  gghighlight::gghighlight(q <= 0.05,
                           label_key = clinical,
                           unhighlighted_params = list(colour = NULL, shape = 21))
```

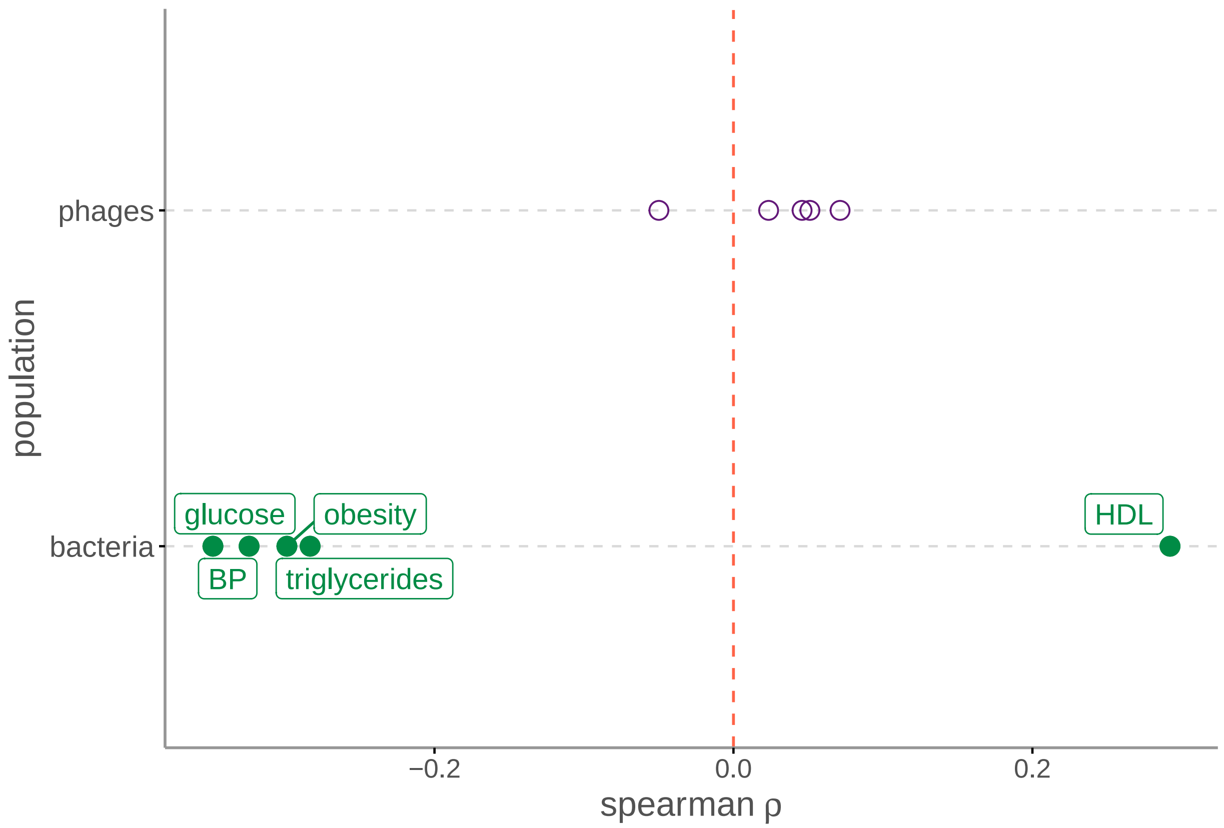

Figure 3: differential abundance per host taxa

load host linkage files and proved base stats

```
VC_share_known <- rio::import("host_linkages/VC_known_host.csv") %>%
  mutate(source = "shared") %>%
  select(contigName, VC_Subcluster, phylum, family, genus, species, source)

VC_spacers <- rio::import("host_linkages/VC_spacer_hits.csv")[,-3] %>%
  mutate(source = "CRISPR") %>%
  select(Viral_contig, VC_Subcluster, phylum,family, genus, species, source) %>%
  dplyr::rename(contigName = Viral_contig)

VC_spacers_PATRIC <- rio::import("host_linkages/VC_spacer_hits_PATRIC.csv") %>%
  mutate(source = "CRISPR") %>%
  select(Viral_contig, VC_Subcluster, phylum,family, genus, species, source) %>%
  dplyr::rename(contigName = Viral_contig)
```

```
VC_prophage <- rio::import("host_linkages/VC_prophage.csv") %>%
  mutate(source = "prophage") %>%
  select(contigName, VC_Subcluster, phylum,family, genus, species, source)

all_host_hits <- rbind(VC_share_known, VC_spacers, VC_spacers_PATRIC, VC_prophage) %>%
  filter(!phylum %in% c("no support", "not classified")) %>%
  distinct() %>%
  tibble()

length(unique(all_host_hits$VC_Subcluster))
```

```
## [1] 7463
```

```
length(unique(all_host_hits$VC_Subcluster))/length(unique(VCs$VC_Subcluster))
```

```
## [1] 0.3408541
```

```
all_host_hits %>%
  group_by(phylum) %>%
  summarise(VC_count = n_distinct(VC_Subcluster)) %>%
  ungroup() %>%
  arrange(-VC_count)
```

```
## # A tibble: 12 x 2
##    phylum                     VC_count
##    <chr>                         <int>
##  1 Firmicutes                     5301
##  2 Bacteroidetes                  1284
##  3 Actinobacteria                  485
##  4 Proteobacteria                  421
##  5 Verrucomicrobia                  66
##  6 Euryarchaeota                    25
##  7 Lentisphaerae                    17
##  8 Tenericutes                      13
##  9 Synergistetes                    10
## 10 Candidatus Melainabacteria        9
## 11 Elusimicrobia                     2
## 12 Chlorobi                          1
```

```
all_host_hits %>%
  group_by(VC_Subcluster) %>%
  summarise(phylum_count = n_distinct(phylum)) %>%
  ungroup() %>%
  filter(phylum_count > 1) %>%
  summarise(n())
```

```
## # A tibble: 1 x 1
##   `n()`
##   <int>
## 1   164
```

create phyloseq with the most common host linkages and perform ANCOM-BC analysis

```
relative.bulk.psmelt <- psmelt(relative.bulk.ps)

all_host_hits %>%
  filter(VC_Subcluster %in% relative.bulk.psmelt$OTU,
         !family %in% c("", "no support", "not classified")) %>%
  group_by(family) %>%
  summarise(VC_count = n_distinct(VC_Subcluster)) %>%
  ungroup() %>%
  arrange(-VC_count) %>%
  top_n(n = 12, VC_count) -> most_common_hosts

raw_reads %>%
  filter(datatype == "WGS") %>%
  inner_join(VCs[,-3], by = "contigName") %>%
  inner_join(distinct(all_host_hits[all_host_hits$family %in%
                                      most_common_hosts$family,-c(1,7)]),
             by = "VC_Subcluster") %>%
  group_by(family, samplename) %>%
  summarise(mapped = sum(mapped)) %>%
  ungroup() %>%
  pivot_wider(names_from = samplename, values_from = mapped, values_fill = 0) %>%
  column_to_rownames("family") %>%
  otu_table(taxa_are_rows = T) -> most_common_families

common.bulk.ps <- phyloseq(most_common_families, relative.bulk.ps@sam_data)

out_common <- ancombc(common.bulk.ps, group = "met_syn", p_adj_method = "BH",
                      zero_cut = 0.9, lib_cut = 0, struc_zero = T, neg_lb = F,
                      tol = 1e-5, max_iter = 100, conserve = T, alpha = 0.05,
                      global = T, formula = "roken + leeftijd + geslacht + alcohol +
                      metformin + met_syn")

common.bulk.ANCOM <- tibble(family = row.names(as.data.frame(out_common$res$beta)),
                            logFoldChange = as.data.frame(out_common$res$beta)$met_synYES,
                            se = as.data.frame(out_common$res$se)$met_synYES,
                            q_val = as.data.frame(out_common$res$q_val)$met_synYES,
                            met_syn = ifelse(logFoldChange > 0, "YES", "NO"))
```

Figure 3A: differential abundance by bacterial host family

```
common.bulk.ANCOM %>%
  ggdotchart(x = "family",
             y = "logFoldChange",
             color = "met_syn",
             rotate = T,
             ggtheme = PAdJ_theme(),
             sorting = "descending",
             size = 1,
             dot.size = 3,
             xlab = "host taxonomy",
```

```
             ylab = "log fold change") +
  geom_linerange(aes(ymin = logFoldChange-se,
                     ymax = logFoldChange+se,
                     color = met_syn),
                 position=position_dodge(.9)) +
  gghighlight::gghighlight(q_val <= 0.05, use_direct_label = F,
                           unhighlighted_params = list(colour = NULL, shape = 21)) +
  theme(axis.text.y = element_text(face = "italic")) +
  geom_hline(yintercept = 0,
             color = "tomato1",
             linetype = "dashed",
             size = 0.5) +
  scale_color_manual(values = MetS.colors, name = "differentially abundant (q < 0.05)", labels = c("con
```

```
## Warning: Using `across()` in `filter()` is deprecated, use `if_any()` or
## `if_all()`.

## Warning: Using `across()` in `filter()` is deprecated, use `if_any()` or
## `if_all()`.
```

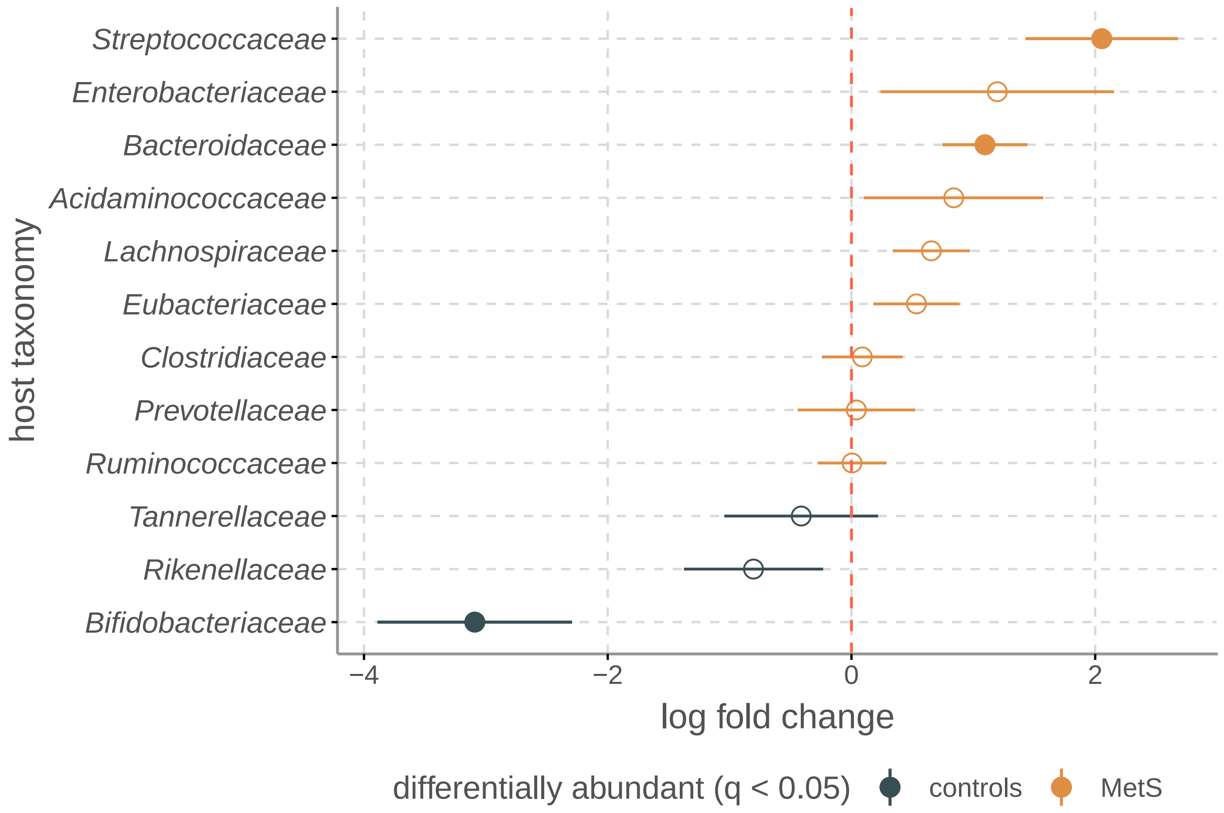

ANCOM-BC of the most common host species targeted by phages from the bulk fractions

```
raw_reads %>%
  filter(datatype == "WGS") %>%
  inner_join(VCs[,-3], by = "contigName") %>%
```

```
  inner_join(distinct(all_host_hits[all_host_hits$family %in%
                                    most_common_hosts$family,-c(1,7)]),
           by = "VC_Subcluster") %>%
  filter(!species %in% c("", "no support", "not classified")) %>%
  mutate(species = ifelse(startsWith(gsub("^\\w+ ", "", species), "sp."),
                        word(species, 1, 3),
                        ifelse(startsWith(species, "uncultured"),
                               word(species, 1, 3),
                               ifelse(startsWith(species,
                                                 "Clostridium cf. saccharolyticum"),
                                      "Clostridium cf. saccharolyticum",
                                      ifelse(startsWith(species,
                                                        "Prevotella sp. BCRC 81118"),
                                             "Prevotella sp. BCRC 81118",
                                                    word(species, 1, 2))))),
         species = gsub("\\[|\\]", "", species),
         species = gsub("\\*", "", species)) %>%
  filter(!is.na(species)) -> edited_species

edited_species %>%
  group_by(species, samplename) %>%
  summarise(mapped = sum(mapped)) %>%
  ungroup() %>%
  group_by(species) %>%
  mutate(prevalence = ifelse(n_distinct(samplename)>(196*0.1), TRUE, FALSE)) %>%
  ungroup() %>%
  filter(prevalence == T) %>%
  pivot_wider(names_from = samplename, values_from = mapped, values_fill = 0) %>%
  column_to_rownames("species") %>%
  otu_table(taxa_are_rows = T) -> most_common_species

common_sp.bulk.ps <- phyloseq(most_common_species, relative.bulk.ps@sam_data)

out_common_sp <- ancombc(common_sp.bulk.ps, group = "met_syn", p_adj_method = "BH",
                         zero_cut = 0.9, lib_cut = 0, struc_zero = T, neg_lb = F,
                         tol = 1e-5, max_iter = 100, conserve = T, alpha = 0.05,
                         global = T, formula = "roken + leeftijd + geslacht + alcohol +
                         metformin + met_syn")

common_sp.bulk.ANCOM <- tibble(species = row.names(as.data.frame(out_common_sp$res$beta)),
                               logFoldChange = as.data.frame(out_common_sp$res$beta)$met_synYES,
                               se = as.data.frame(out_common_sp$res$se)$met_synYES,
                               q_val = as.data.frame(out_common_sp$res$q_val)$met_synYES,
                               met_syn = ifelse(logFoldChange > 0, "YES", "NO"))
```

Figure 3B: differential abundance by bacterial species in bulk datasets

```
common_sp.bulk.ANCOM %>%
  filter(q_val < 0.05) %>%
  inner_join(distinct(edited_species[,c(8,10)]) , by = "species") %>%
  mutate(family = factor(family, levels = c("Bifidobacteriaceae", "Eubacteriaceae",
```

```
                                          "Rikenellaceae", "Lachnospiraceae",
                                          "Ruminococcaceae", "Clostridiaceae",
                                         "Tannerellaceae", "Streptococcaceae",
                                         "Bacteroidaceae"))) %>%
ggdotchart(x = "species",
           y = "logFoldChange",
           color = "met_syn",
           rotate = T,
           ggtheme = PAdJ_theme(),
           sorting = "descending",
           size = 1,
           dot.size = 3,
           xlab = "host taxonomy",
           ylab = "log fold change",
           group = "family") +
geom_linerange(aes(ymin = logFoldChange-se,
                   ymax = logFoldChange+se,
                   color = met_syn),
               position=position_dodge(.9))  +
gghighlight::gghighlight(q_val <= 0.05, use_direct_label = F,
                         unhighlighted_params = list(colour = NULL, shape = 21)) +
theme(axis.text.y = element_text(face = "italic", size = 13),
      legend.position = "") +
geom_hline(yintercept = 0,
           color = "tomato1",
           linetype = "dashed",
           size = 0.5) +
scale_color_manual(values = MetS.colors,
                   name = "differentially abundant (q < 0.05)",
                   labels = c("controls", "MetS"))
```

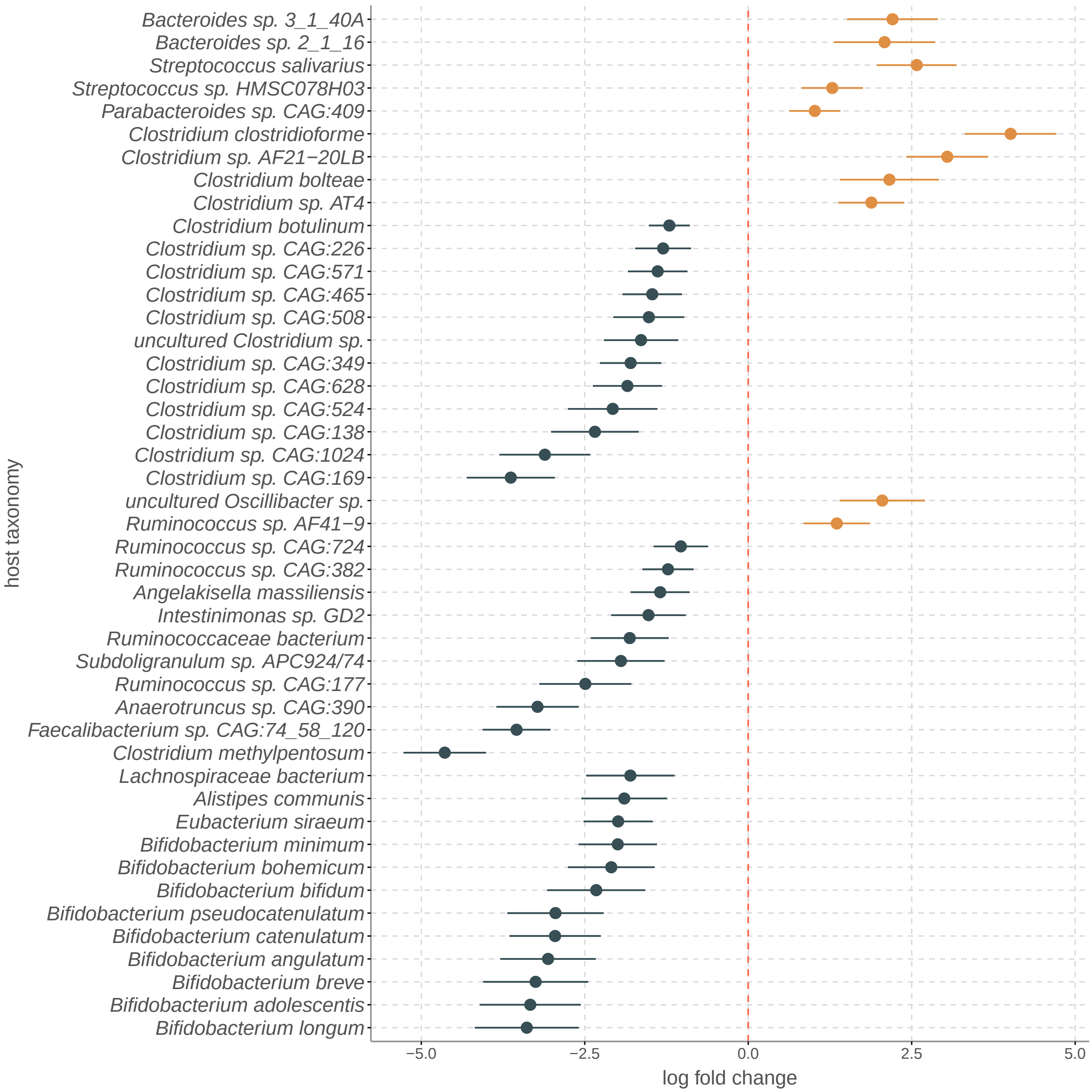

ANCOM-BC of the most common host species targeted by phages from the VLP fractions

```
raw_reads %>%
  filter(datatype == "VLP") %>%
  inner_join(VCs[,-3], by = "contigName") %>%
  inner_join(distinct(all_host_hits[all_host_hits$family %in%
                                    most_common_hosts$family,-c(1,7)]),
             by = "VC_Subcluster") %>%
  filter(!species %in% c("", "no support", "not classified")) %>%
  mutate(species = ifelse(startsWith(gsub("^\\w+ ", "", species), "sp."),
                          word(species, 1, 3),
                          ifelse(startsWith(species, "uncultured"),
                                 word(species, 1, 3),
                                 ifelse(startsWith(species,
```

```
                                                 "Clostridium cf. saccharolyticum"),
                                        "Clostridium cf. saccharolyticum",
                                        ifelse(startsWith(species,
                                                        "Prevotella sp. BCRC 81118"),
                                               "Prevotella sp. BCRC 81118",
                                                      word(species, 1, 2))))),
         species = gsub("\\[|\\]", "", species),
         species = gsub("\\*", "", species)) %>%
  filter(!is.na(species)) -> edited_species.VLP

edited_species.VLP %>%
  group_by(species, samplename) %>%
  summarise(mapped = sum(mapped)) %>%
  ungroup() %>%
  group_by(species) %>%
  mutate(prevalence = ifelse(n_distinct(samplename)>(48*0.1), TRUE, FALSE)) %>%
  ungroup() %>%
  filter(prevalence == T) %>%
  pivot_wider(names_from = samplename, values_from = mapped, values_fill = 0) %>%
  column_to_rownames("species") %>%
  otu_table(taxa_are_rows = T) -> most_common_species.VLP

common_sp.VLP.ps <- phyloseq(most_common_species.VLP, relative.bulk.ps@sam_data)

out_common_sp.VLP <- ancombc(common_sp.VLP.ps, group = "met_syn", p_adj_method = "BH",
                             zero_cut = 0.9, lib_cut = 0, struc_zero = T, neg_lb = F,
                             tol = 1e-5, max_iter = 100, conserve = T, alpha = 0.05,
                             global = T,
                             formula = "roken + leeftijd + geslacht + alcohol + metformin + met_syn")

common_sp.VLP.ANCOM <- tibble(species = row.names(as.data.frame(out_common_sp.VLP$res$beta)),
                              logFoldChange = as.data.frame(out_common_sp.VLP$res$beta)$met_synYES,
                              se = as.data.frame(out_common_sp.VLP$res$se)$met_synYES,
                              q_val = as.data.frame(out_common_sp.VLP$res$q_val)$met_synYES,
                              met_syn = ifelse(logFoldChange > 0, "YES", "NO"))
```

Figure 3C: differential abundance by bacterial species in VLP datasets

```
common_sp.VLP.ANCOM %>%
  filter(q_val < 0.05) %>%
  inner_join(distinct(edited_species.VLP[,c(8,10)]) , by = "species") %>%
  mutate(family = factor(family, levels = c("Rikenellaceae", "Clostridiaceae", "Prevotellaceae",
                                            "Bacteroidaceae", "Lachnospiraceae", "Ruminococcaceae"))) %>
  ggdotchart(x = "species",
             y = "logFoldChange",
             color = "met_syn",
             rotate = T,
             ggtheme = PAdJ_theme(),
             sorting = "descending",
             size = 1,
             dot.size = 3,
```

```
            xlab = "host taxonomy",
            ylab = "log fold change",
            group = "family") +
  geom_linerange(aes(ymin = logFoldChange-se,
                     ymax = logFoldChange+se,
                     color = met_syn),
                 position=position_dodge(.9))  +
  gghighlight::gghighlight(q_val <= 0.05, use_direct_label = F,
                           unhighlighted_params = list(colour = NULL, shape = 21)) +
  theme(axis.text.y = element_text(face = "italic", size = 13),
        legend.position = "") +
  geom_hline(yintercept = 0,
             color = "tomato1",
             linetype = "dashed",
             size = 0.5) +
  scale_color_manual(values = MetS.colors,
                     name = "differentially abundant (q < 0.05)",
                     labels = c("controls", "MetS"))
```

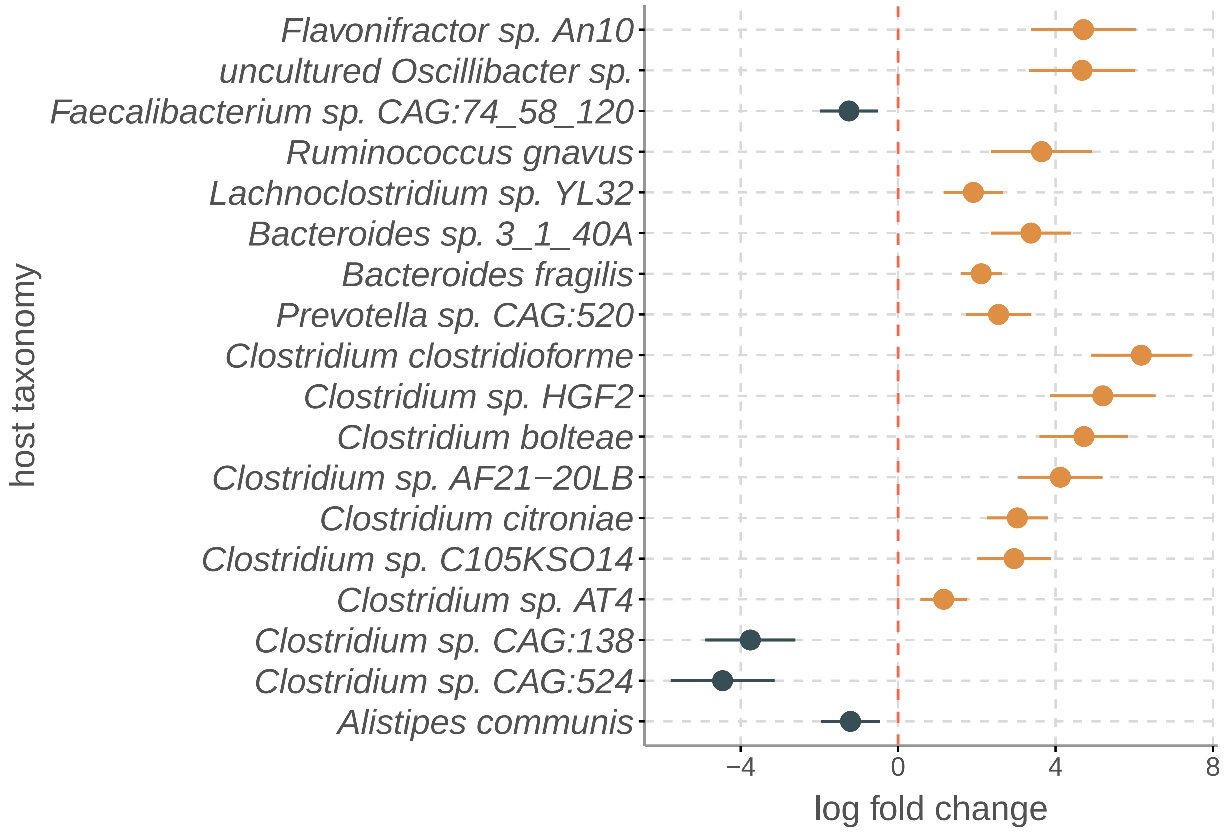

Figure 4: differential abundance of VCs

ANCOM-BC on VCs

```
VCs.ANCOM <- ancombc(counts.bulk.ps, group = "met_syn", p_adj_method = "BH", zero_cut = 0.8,
                     lib_cut = 0, struc_zero = T, neg_lb = F, tol = 1e-5, max_iter = 100,
                     conserve = T, alpha = 0.001, global = T,
                     formula = "roken + leeftijd + geslacht + alcohol + metformin + met_syn")

VCs.ANCOM.res <- tibble(VC_Subcluster = row.names(as.data.frame(VCs.ANCOM$res$beta)),
                        logFoldChange = as.data.frame(VCs.ANCOM$res$beta)$met_synYES,
                        se = as.data.frame(VCs.ANCOM$res$se)$met_synYES,
                        q_val = as.data.frame(VCs.ANCOM$res$q_val)$met_synYES,
                        met_syn = ifelse(logFoldChange > 0, "YES", "NO")) %>%
  filter(q_val < 0.05)
```

Figure 4a: ANCOM-BC analysis of VCs

```
VCs.ANCOM.res %>%
  mutate(VC_Subcluster = gsub("_cov.+$", "...", VC_Subcluster)) %>%
   ggdotchart(x = "VC_Subcluster",
              y = "logFoldChange",
              color = "met_syn",
              rotate = T,
              ggtheme = PAdJ_theme(),
              sorting = "descending",
              size = 1,
              dot.size = 3,
              xlab = "VC",
              ylab = "log fold change",
              palette = MetS.colors) +
    geom_linerange(aes(ymin = logFoldChange-se,
                       ymax = logFoldChange+se,
                       color = met_syn),
                   position=position_dodge(.9))
```

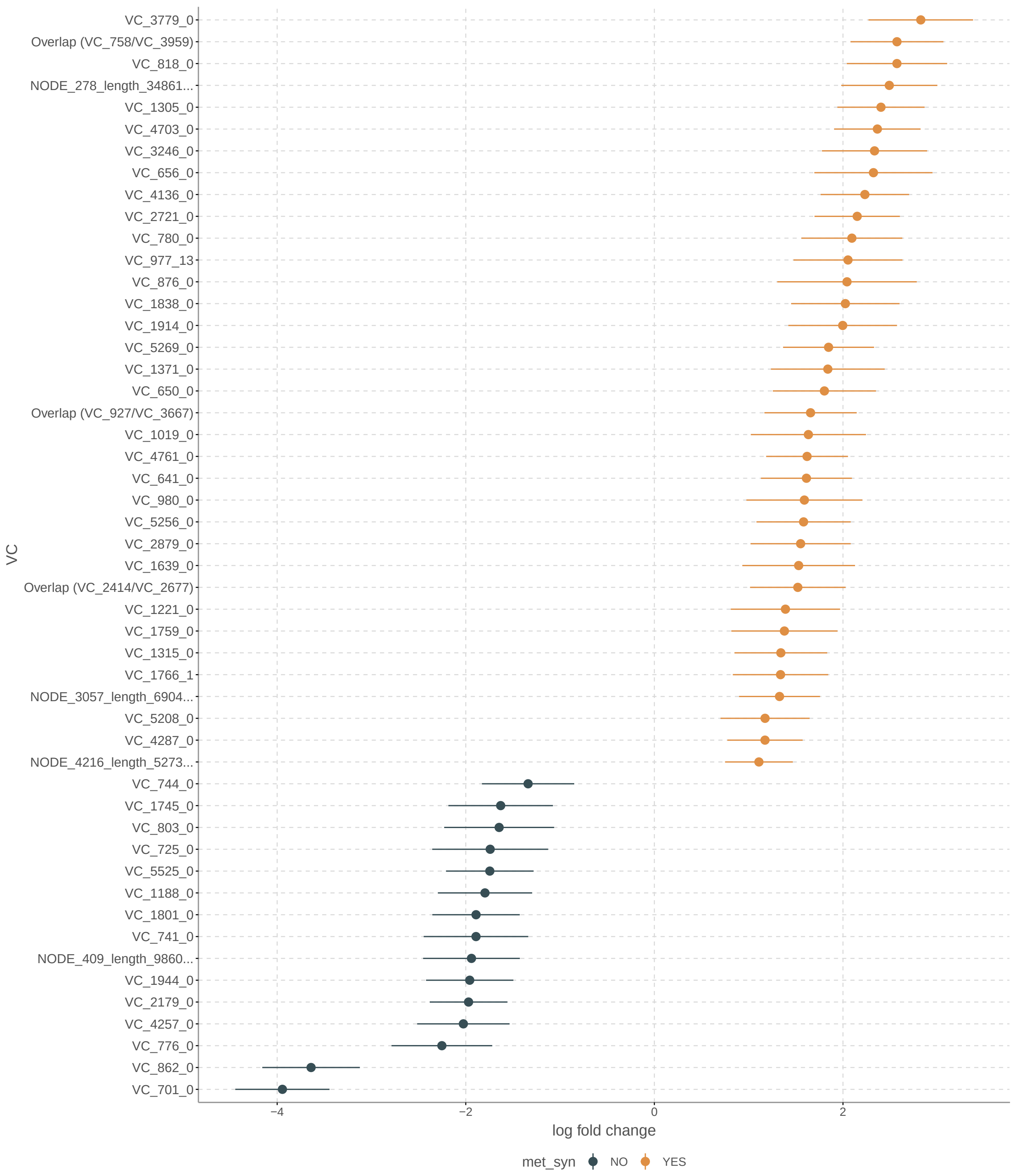

Figure 4b (1): relative abundance of NODE_38 in 2 samples

```
rio::import("datafiles/NODE_38_2samples.csv") %>%
  pivot_longer(-1, "sample") %>%
  ggplot(aes(x = position,
```

```
              y = value,
              color = sample)) +
  geom_line() +
  scale_y_continuous(name = "coverage") +
  scale_x_continuous(name = "position (bp)",
                     limits = c(37734,131241),
                     expand = c(0,0)) +
  scale_color_manual(values = get_palette("uchicago", 2)) +
  PAdJ_theme() +
  theme(axis.line.x = element_blank(),
        axis.line.y = element_blank(),
        legend.position = "")
```

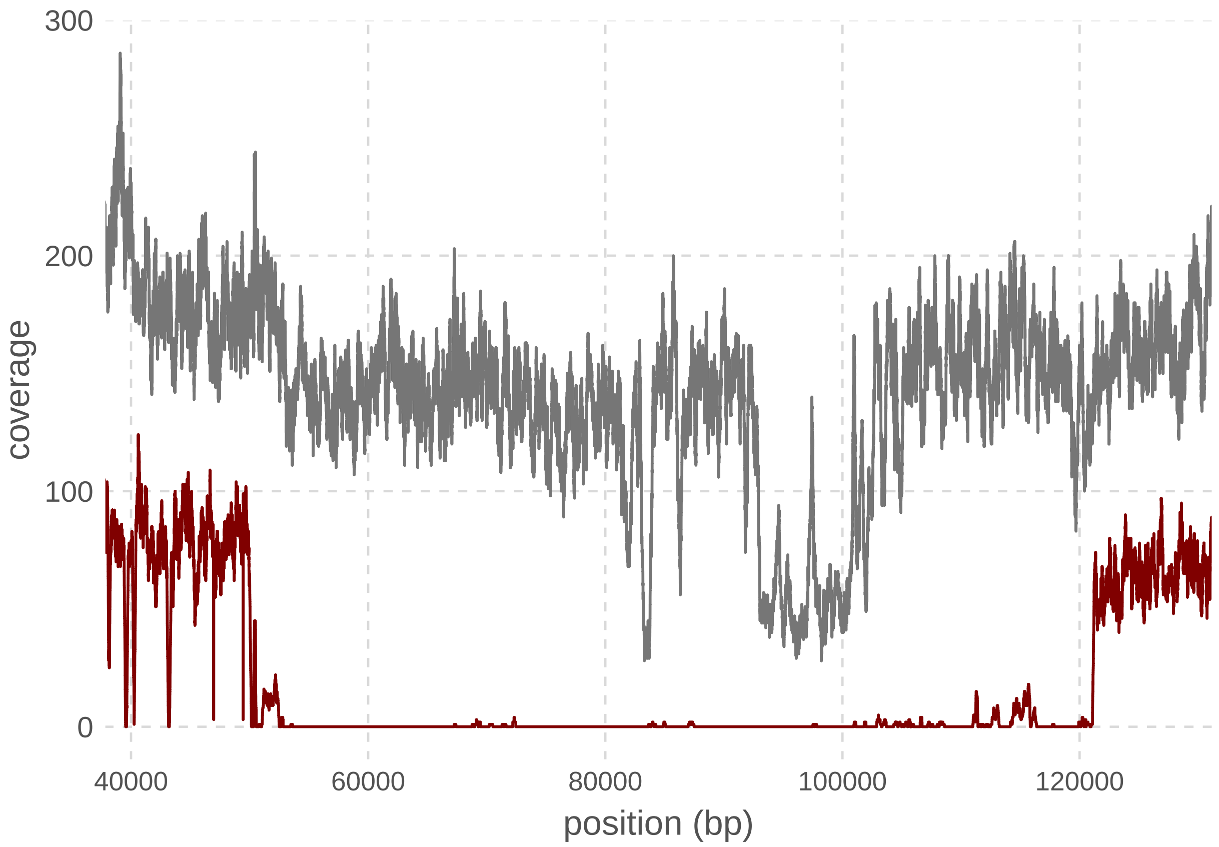

Figure 4b (2): relative abundance of NODE_4 in 3 samples

```
rio::import("datafiles/NODE_4_3samples.csv") %>%
  filter(position < 400000,
         position > 320000) %>%
  ggline(x = "position",
         y = "reads",
         ggtheme = PAdJ_theme(),
         color = "sample",
         palette = c(get_palette("uchicago", 4)[2],
```

```
                     get_palette("uchicago", 4)[1],
                     get_palette("uchicago", 4)[3]),
        plot_type = "l",
        numeric.x.axis = T) +
  scale_x_reverse()
```

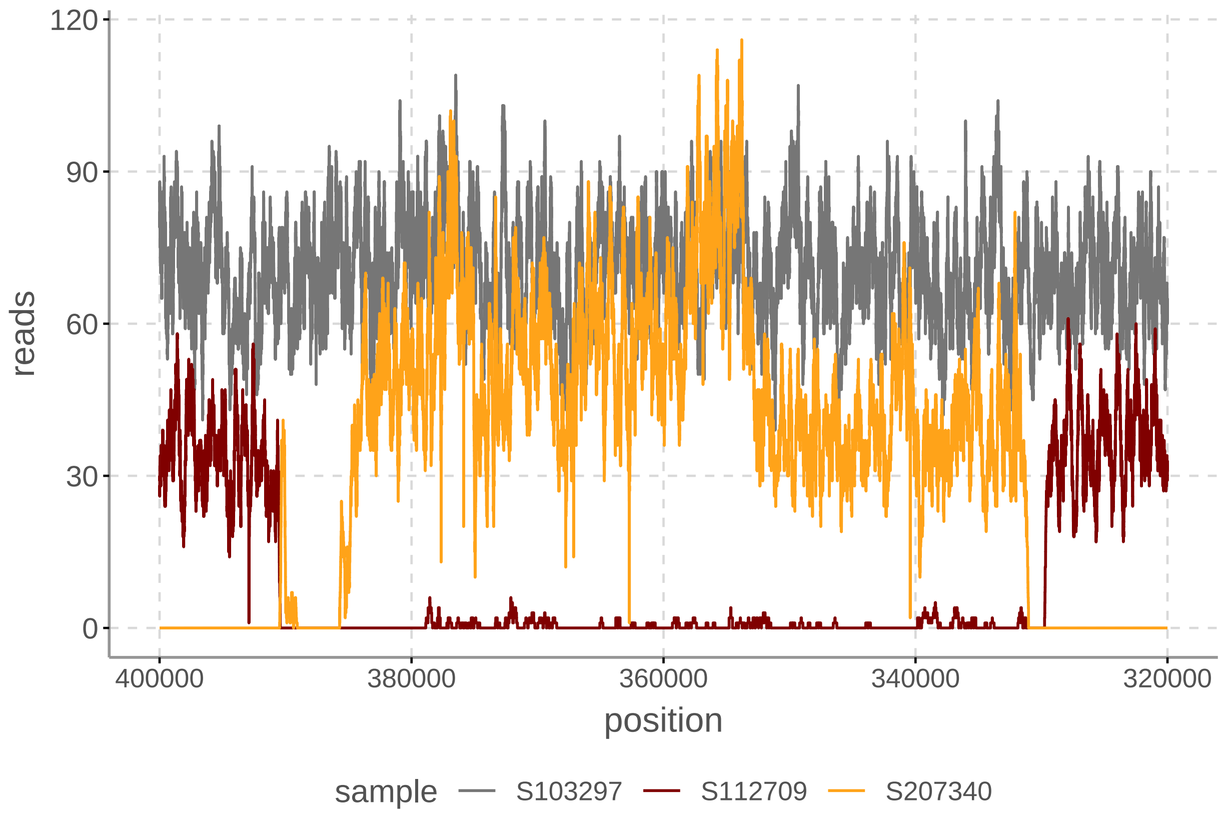

Figure 5: Ca. Heliusviridae

protein cluster data of Ca. Heliusviridae phages

```
heliusviridae <- rio::import("heliusviridae_files/heliusviridae.csv")

PCs <- read.csv("heliusviridae_files/heliusviridae_PCs.csv")

PCs %>%
  rowwise() %>%
  mutate(cluster = gsub("^$", protein_id, cluster)) %>%
  group_by(contig_id) %>%
  summarise(PCs = n_distinct(cluster)) %>%
  ungroup() -> PCs_per_contig

PCs %>%
  select(one_of("protein_id", "contig_id")) %>%
```

```
  mutate(ORF_nr = as.numeric(gsub(".*_", "", protein_id))) %>%
  group_by(contig_id) %>%
  summarise(ORFs = max(ORF_nr)) -> ORFs_per_contig

PCs %>%
  rowwise() %>%
  mutate(cluster = gsub("^$", protein_id, cluster)) %>%
  select(one_of("contig_id", "cluster")) %>%
  distinct() -> x

data_matrix <- as.data.frame(crossprod(table(x[,c(2,1)]))) %>%
  rownames_to_column("contig_1") %>%
  pivot_longer(-1,
               names_to = "contig_2",
               values_to = "shared_pc") %>%
  left_join(PCs_per_contig, by = c("contig_1"="contig_id")) %>%
  dplyr::rename(PCs_1 = PCs) %>%
  left_join(PCs_per_contig, by = c("contig_2"="contig_id")) %>%
  dplyr::rename(PCs_2 = PCs) %>%
  mutate(shared_percent = ifelse(PCs_1 > PCs_2,
                                 shared_pc/PCs_2*100, shared_pc/PCs_1*100)) %>%
  select(one_of("contig_1", "contig_2", "shared_percent")) %>%
  pivot_wider(names_from = contig_2,
              values_from = shared_percent) %>%
  column_to_rownames("contig_1")
```

Figure 5a: heatmap of Ca. Heliusviridae by % shared protein clusters

```
NB_res2 <-  NbClust::NbClust(data_matrix,
                             diss=NULL,
                             distance = "euclidean",
                             method = "average",
                             index = "ch",
                             max.nc = 8,
                             min.nc = 3)

as.data.frame(NB_res2$Best.partition) %>%
  transmute(group = ifelse(NB_res2$Best.partition == 1, "alpha",
                           ifelse(NB_res2$Best.partition == 2, "gamma",
                                  "beta")))%>%
  rownames_to_column("contigName") %>%
  mutate(sign = ifelse(gsub("deJonge.+NODE", "NODE", contigName) %in%
                         gsub("_S.+$", "",
                              VCs[VCs$VC_Subcluster == "VC_818_0",]$contigName),
                       "VC_818_0",
                       ifelse(gsub("deJonge.+NODE", "NODE", contigName) %in%
                                gsub("_S.+$", "",
                                     VCs[VCs$VC_Subcluster == "VC_1801_0",]$contigName),
                              "VC_1801_0",
                              ifelse(gsub("deJonge.+NODE", "NODE", contigName) %in%
                                       gsub("_S.+$", "",
```

```
                                            VCs[VCs$VC_Subcluster == "VC_803_0",]$contigName),
                                     "VC_803_0",
                                     ifelse(gsub("deJonge.+NODE", "NODE", contigName) %in%
                                              gsub("_S.+$", "",
                                                   VCs[VCs$VC_Subcluster == "VC_1639_0",]$contigName),
                                            "VC_1639_0", "none"))))) %>%
  column_to_rownames("contigName") -> heatmap_colors

colors <- list(group = c(alpha = "#1b9e77",
                        beta = "#e6ab02",
                        gamma = "#7570b3"),
              sign = c(VC_818_0 = "#9251ed",
                       VC_1801_0 = "#ed5e51",
                       VC_803_0 = "#258f2a",
                       VC_1639_0 = "#569db0",
                       none = "white"))

pheatmap::pheatmap(as.matrix(data_matrix),
                   color = viridis(n = 100,
                                   option = "inferno"),
                   show_rownames = F,
                   show_colnames = F,
                   clustering_distance_rows = "euclidean",
                   clustering_distance_cols = "euclidean",
                   clustering_method = "average",
                   cutree_rows = 3,
                   cutree_cols = 3,
                   annotation_col = heatmap_colors,
                   annotation_colors = colors,
                   annotation_legend = F)
```

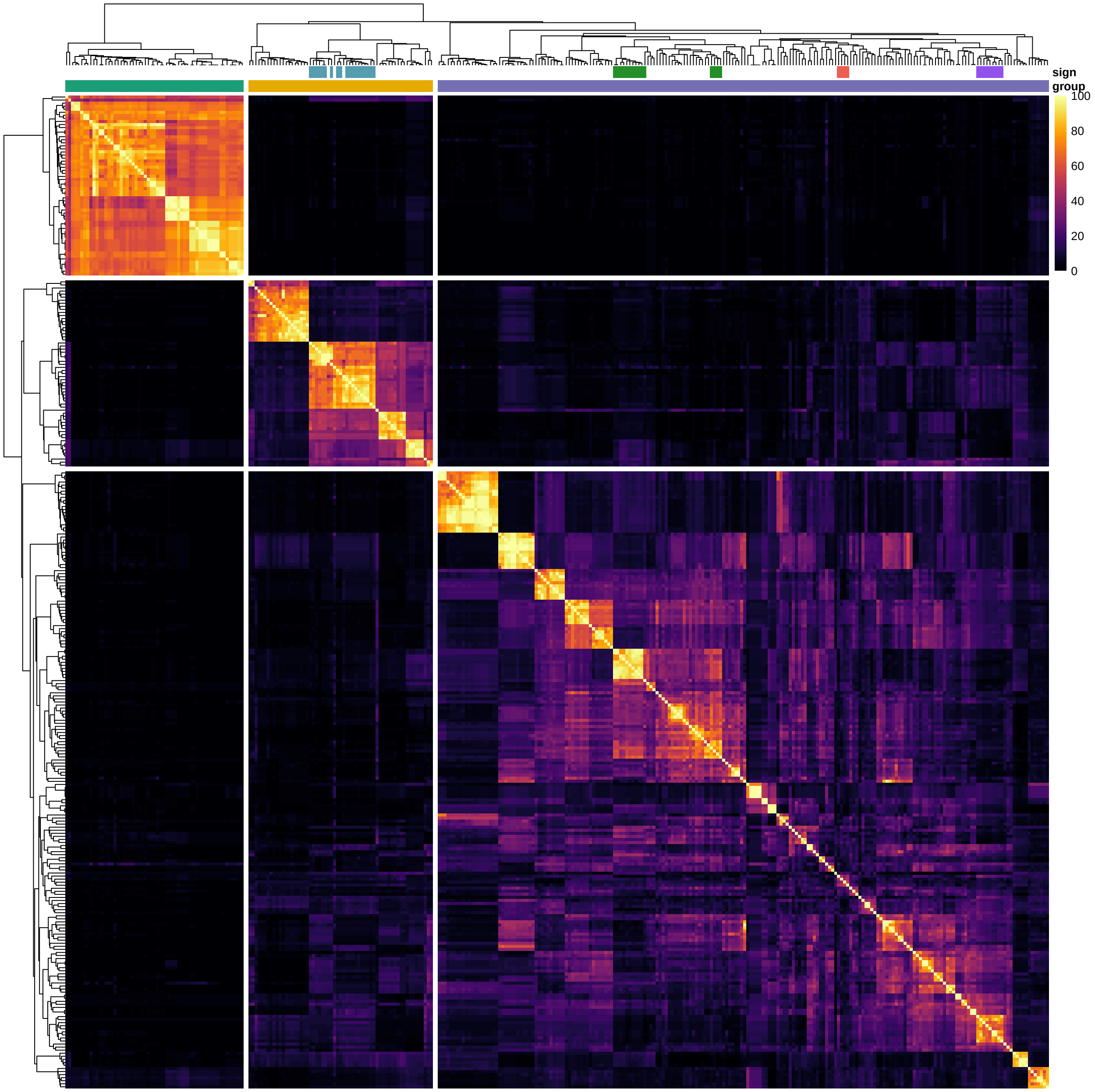

Figure 5c: prevalence of Ca. Heliusviridae in among samples

```
heatmap_colors %>%
  rownames_to_column("contigName") %>%
  mutate(contigName = gsub("deJonge_", "", contigName)) %>%
  filter(startsWith(contigName, "NODE")) %>%
  tibble() -> heliusviridae

relative.VLP.psmelt <- psmelt(relative.VLP.ps)

do.call(rbind, lapply(list(relative.bulk.psmelt, relative.VLP.psmelt), function(x){
  heliusviridae %>%
```

```r
    inner_join(VCs %>% mutate(contigName = gsub("_S.+$", "", contigName)),
               by = "contigName") %>%
    select(VC_Subcluster, group) %>%
    distinct() %>%
    inner_join(x[,c(1:3, 10)], by = c("VC_Subcluster"="OTU")) %>%
    group_by(Sample, met_syn) %>%
    summarise(category = ifelse(sum(Abundance) == 0,
                                "none",
                                ifelse(n_distinct(group[Abundance > 0]) == 2,
                                       "both",
                                       ifelse(unique(group[Abundance > 0]) == "beta",
                                              "beta", "gamma")))) %>%
    ungroup() %>%
    group_by(category, met_syn) %>%
    summarise(participants = n_distinct(Sample)) %>%
    ungroup() %>%
    mutate(category = factor(category,
                             levels = c("none", "both", "gamma", "beta")),
           dataset = ifelse(sum(participants) == 196,
                            "bulk viromes", "VLP viromes"))
})) -> heliusviridae_prevalence

ggbarplot(data = heliusviridae_prevalence,
          x = "met_syn",
          y = "participants",
          color = "category",
          fill = "category",
          alpha = 0.5,
          ggtheme = PAdJ_theme(),
          rotate = T,
          facet.by = "dataset",
          ncol = 1,
          scales = "free_x") +
  scale_color_manual(values = c("grey", "#cc6666", "#7570b3", "#e6ab02")) +
  scale_fill_manual(values = c("grey", "#cc6666", "#7570b3", "#e6ab02")) +
  theme(panel.grid.major.y = element_blank(),
        strip.background = element_rect(color = "white", fill = "white"),
        strip.text = element_text(size = 14, face = "bold")) +
  scale_x_discrete("group",
                   labels = c("controls", "MetS"))
```

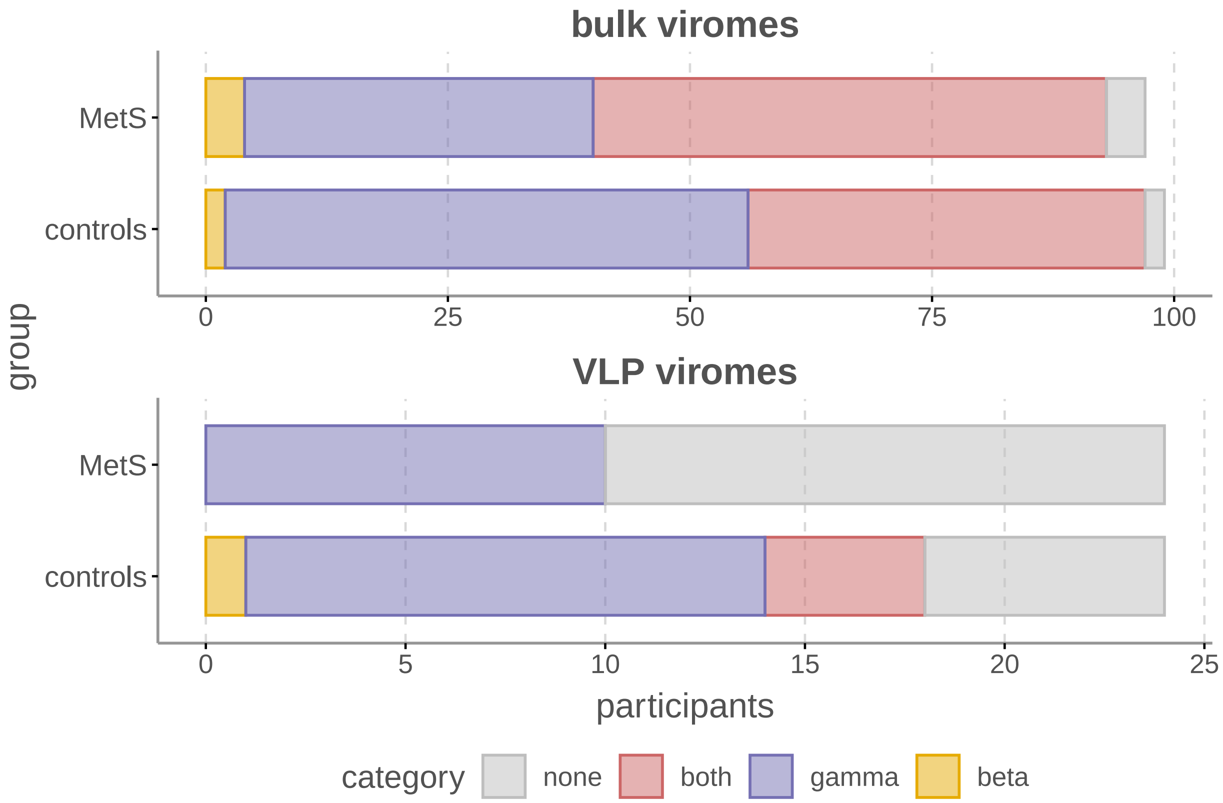

Figure 5c: relative abundance of heliusviridae

```
heliusviridae %>%
  inner_join(VCs %>% mutate(contigName = gsub("_S.+$", "", contigName)),
             by = "contigName") %>%
  rowwise() %>%
  inner_join(relative.bulk.psmelt[,c(1:3, 10)], by = c("VC_Subcluster"="OTU")) %>%
  group_by(Sample, met_syn) %>%
  summarise(rel_abundance = sum(Abundance)+0.005) %>%
  ungroup() %>%
  mutate(rel_abundance = rel_abundance,
        met_syn = factor(met_syn, levels = c("NO", "YES")),
        group = "all") -> Helius_all_abundance

heliusviridae %>%
  inner_join(VCs %>% mutate(contigName = gsub("_S.+$", "", contigName)),
             by = "contigName") %>%
  rowwise() %>%
  inner_join(relative.bulk.psmelt[,c(1:3, 10)], by = c("VC_Subcluster"="OTU")) %>%
  group_by(Sample, met_syn, group) %>%
  summarise(rel_abundance = sum(Abundance)) %>%
  ungroup() %>%
  mutate(group = factor(group,
                        levels = c("all", "beta", "gamma")),
```

```
       rel_abundance = rel_abundance+0.005,
       met_syn = factor(met_syn, levels = c("NO", "YES"))) %>%
  rbind(Helius_all_abundance) %>%
  ggviolin(x = "met_syn",
           y = "rel_abundance",
           color = "met_syn",
           fill = "met_syn",
           alpha = 0.4,
           rotate = T,
           palette = MetS.colors,
           ggtheme = PAdJ_theme(),
           add = c("boxplot"),
           add.params = list(fill = "white",
                             width = 0.3),
           trim = T) +
  theme(legend.position = "",
        strip.background = element_rect(fill = "white", color = "white"),
        strip.text = element_text(size = 14, face = "bold")) +
  facet_wrap(~group,
             ncol = 1) +
  stat_compare_means(label = "p.signif",
                     hide.ns = T,
                     vjust = -0.5,
                     size = 8) +
  scale_y_log10(name = "rel. abundance",
                     labels = scales::label_percent(),
                     limits = c(0.005,1)) +
  scale_x_discrete(name = "group",
                   labels = c("controls", "MetS"))
```

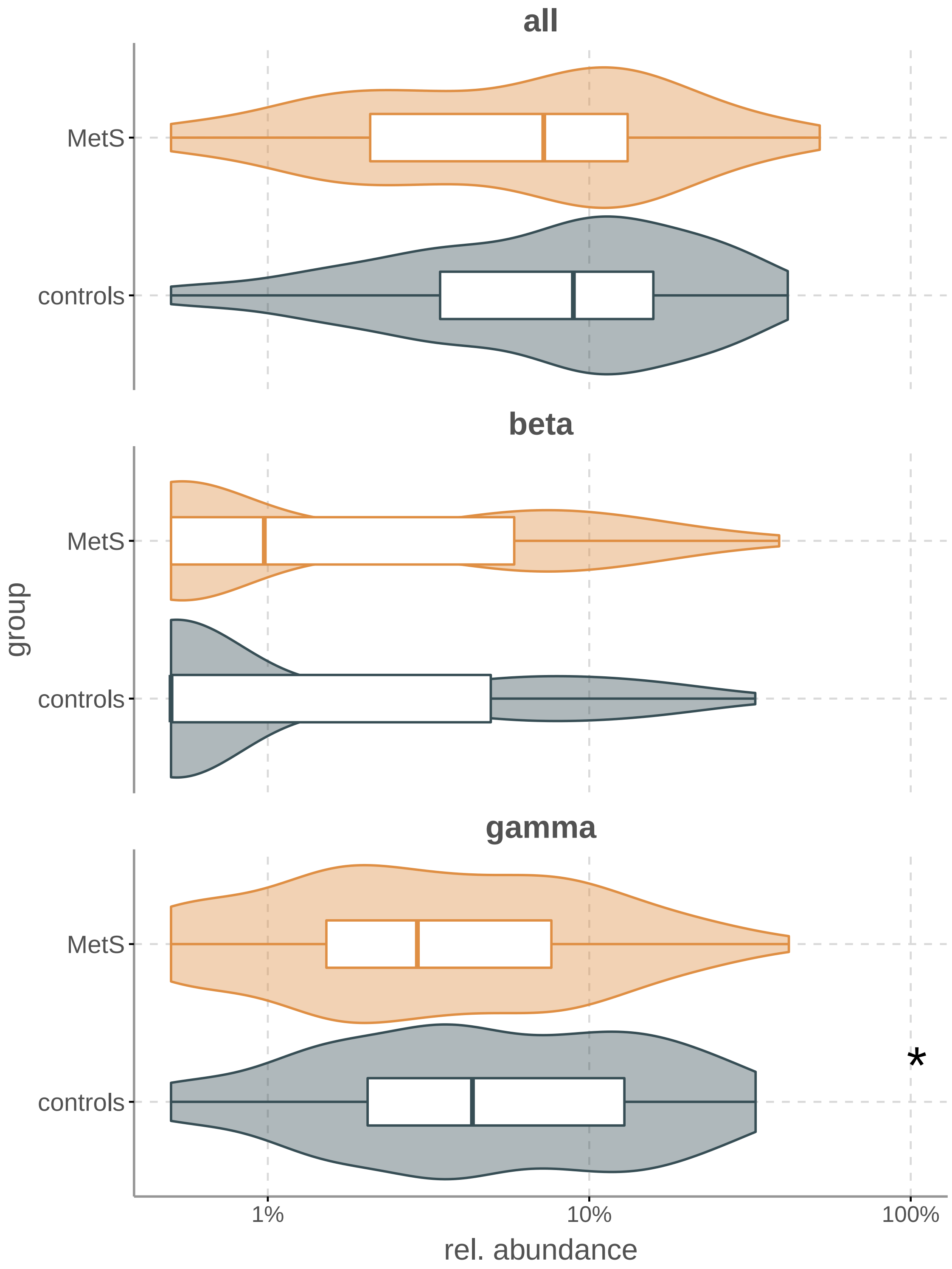
all
MetS
controls
beta
MetS
controls
group
gamma
MetS
controls
*
1%
10%
100%
rel. abundance

Figure 6: VC_818_0 read depth of host genes

Figure 6b: mean host coverage of host-derived regions in NODE_38

```
rio::import("datafiles/NODE_38_coverage.csv") %>%
  pivot_longer(2:197, names_to = "samplename", values_to = "read_depth") %>%
  left_join(metadata[,c(8,10)], by = "samplename") %>%
  filter(!position %in% c(50314:121079)) %>%
  group_by(met_syn, samplename) %>%
  summarise(mean_depth_bac = mean(read_depth),
            error_depth_bac = sd(read_depth),
            hor_coverage = n_distinct(position[read_depth>0])/137219) %>%
  ungroup() %>%
  filter(hor_coverage >= 0.75) -> bacterium

MetS_violin_box(bacterium,
                "mean_depth_bac",
                "mean host gene coverage") +
  scale_y_log10(limits = c(1, 250),
                breaks = c(1, 10, 100),
                labels = c(1, 10, 100))
```

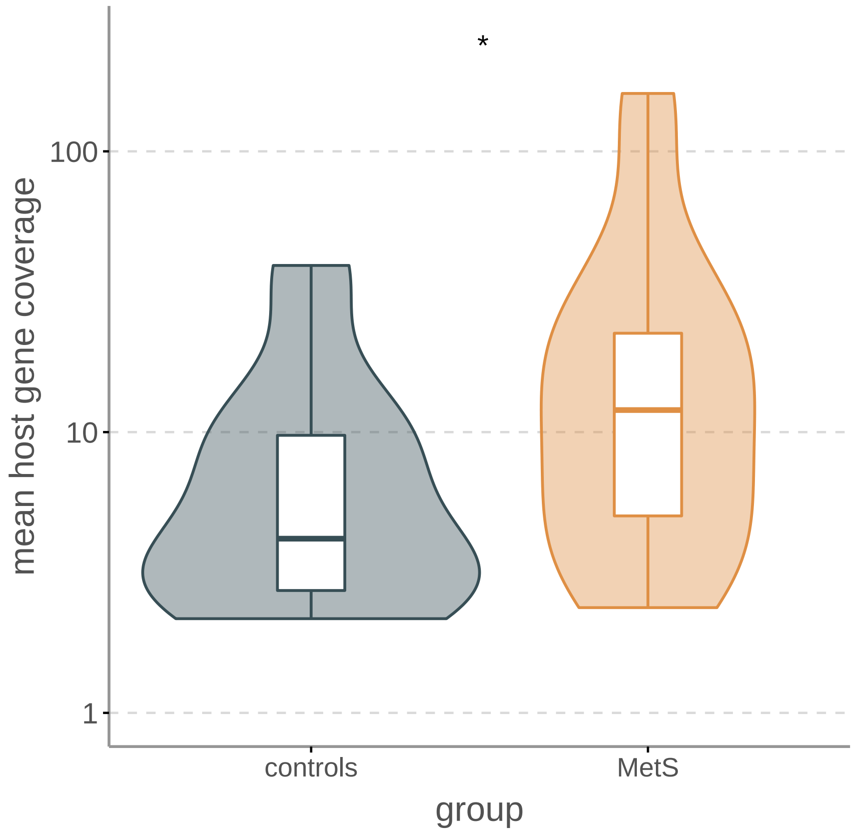

Figure 6c: mean host coverage of host-derived regions in NODE_192

```
rio::import("datafiles/NODE_192_coverage.csv") %>%
  pivot_longer(2:197, names_to = "samplename", values_to = "read_depth") %>%
  left_join(metadata[,c(8,10)], by = "samplename") %>%
  filter(!position > c(50476)) %>%
  group_by(met_syn, samplename) %>%
  summarise(mean_depth_bac = mean(read_depth),
            error_depth_bac = sd(read_depth),
            hor_coverage = n_distinct(position[read_depth>0])/(108507-50476)) %>%
  ungroup() %>%
  filter(hor_coverage >= 0.75) -> bacterium_NODE_192

MetS_violin_box(bacterium_NODE_192,
                "mean_depth_bac",
                "mean host gene coverage") +
  scale_y_log10(limits = c(1, 250),
                breaks = c(1, 10, 100),
                labels = c(1, 10, 100))
```

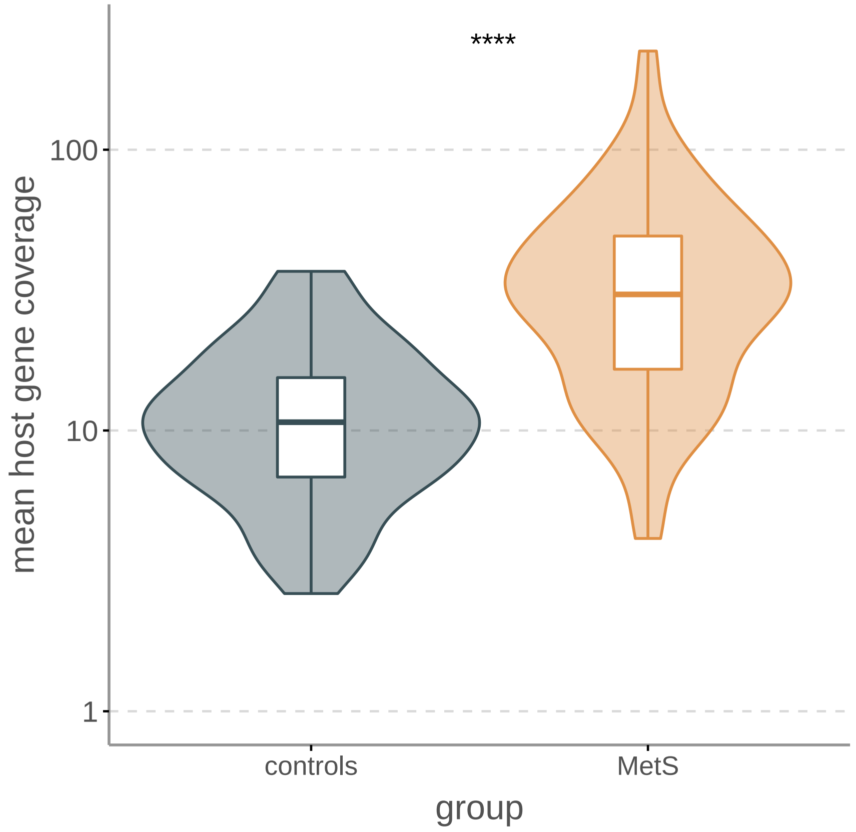

Supplementary Figure 2: prevalence and abundance of VCs

Supplementary Figure 2a: Prevalence of VCs in WGS samples

```
relative.bulk.psmelt %>%
  group_by(OTU) %>%
  filter(Abundance > 0) %>%
  summarise(present = n()) %>%
  ungroup() %>%
  mutate(percent_present = present/196*100,
         category = ifelse(present == 1, "individual-specific",
                          ifelse(percent_present <= 10, "<10",
                                 ifelse(percent_present <= 30, "10-30",
                                        ">30")))) -> cluster_presence.bulk

cluster_presence.bulk %>%
  group_by(category) %>%
  summarise(n_VC = n()) %>%
  ungroup() %>%
  ggdotchart(x = "category",
             y = "n_VC",
             color = "category",
             ggtheme = PAdJ_theme(),
             xlab = "prevalence",
             ylab = "number of VCs",
             sorting = "asc",
             dot.size = 11,
             add = "segment",
             add.params = list(size = 3),
             label = "n_VC",
             font.label = list(color = "white", size = 9,
                               vjust = 0.5),
             palette = category.colors)  +
  theme(legend.position = "",
        aspect.ratio = 1) +
  scale_x_discrete(labels = c(">30%", "10-30%", "<10%", "individual-specific"))
```

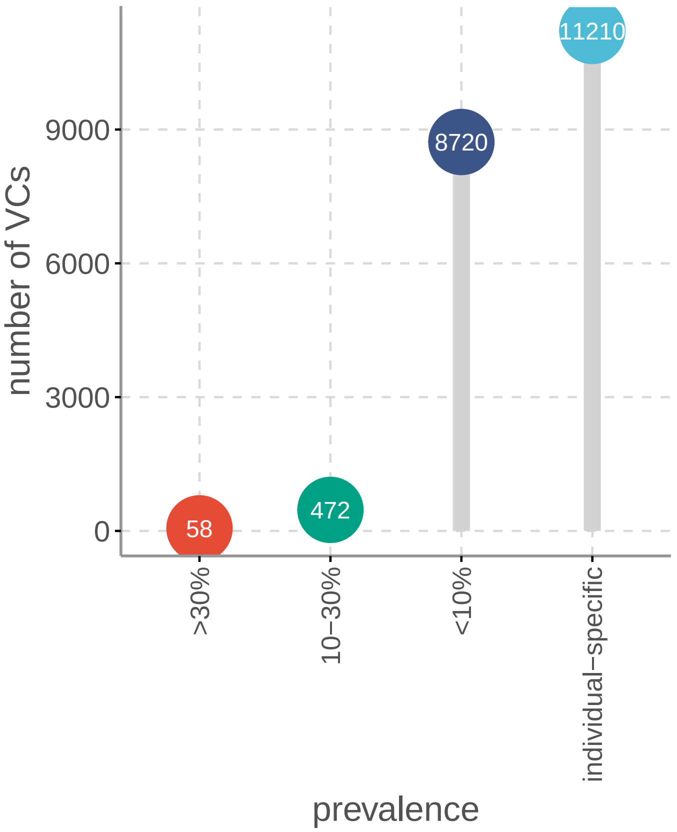

Supplementary Figure 2b: Prevalence of VCs in VLP samples

```
relative.VLP.psmelt %>%
  group_by(OTU) %>%
  filter(Abundance > 0) %>%
  summarise(present = n()) %>%
  ungroup() %>%
  mutate(percent_present = present/48*100,
         category = ifelse(present == 1, "individual-specific",
                      ifelse(percent_present <= 10, "<10",
                             ifelse(percent_present <= 30, "10-30",
                                    ">30")))) -> cluster_presence.VLP

cluster_presence.VLP %>%
  group_by(category) %>%
  summarise(n_VC = n()) %>%
  ungroup() %>%
  ggdotchart(x = "category",
             y = "n_VC",
             color = "category",
             ggtheme = PAdJ_theme(),
             xlab = "prevalence",
             ylab = "number of VCs",
             sorting = "asc",
```

```
              dot.size = 11,
              add = "segment",
              add.params = list(size = 3),
              label = "n_VC",
              font.label = list(color = "white", size = 9,
                                vjust = 0.5),
              palette = category.colors)  +
  theme(legend.position = "",
        aspect.ratio = 1) +
  scale_x_discrete(labels = c(">30%", "10-30%", "<10%", "individual-specific"))
```

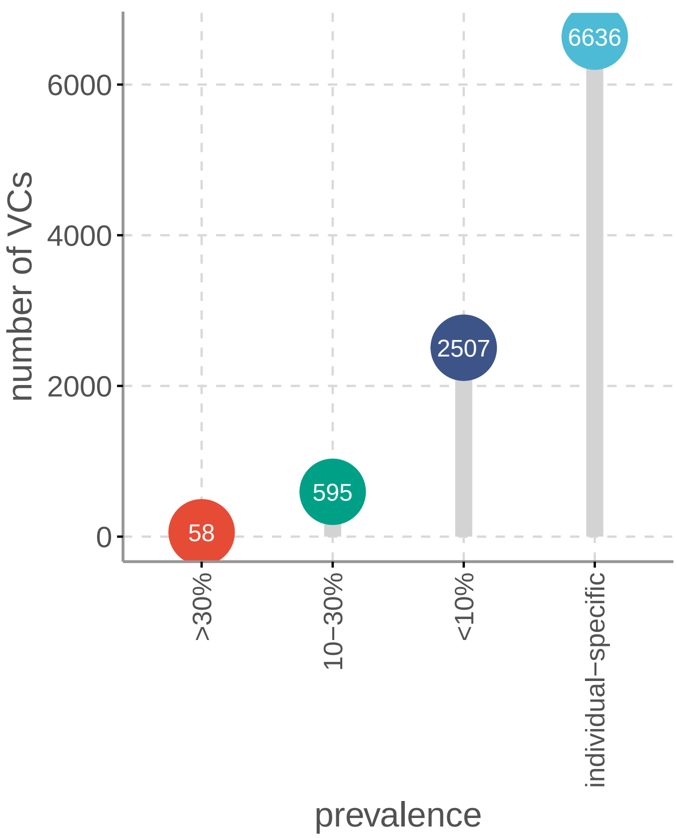

Supplementary Figure 2c: Relative abundance of VCs by their prevalence in WGS samples

```
relative.bulk.psmelt %>%
  left_join(cluster_presence.bulk[,c(1,4)], by = "OTU") %>%
  mutate(category = gsub("individual-specific", "<10", category)) %>%
  group_by(Sample, category) %>%
  summarise(Abundance = sum(Abundance)) %>%
  ungroup() %>%
  mutate(category = factor(category, levels = c("<10", "10-30", ">30"))) %>%
  ggviolin(x = "category",
           y = "Abundance",
```

```
            color = "category",
            fill = "category",
            palette = category.colors,
            alpha = 0.4,
            add = "boxplot",
            add.params = list(fill = "white"),
            ggtheme = PAdJ_theme(),
            ylab = "relative abundance",
            xlab = "prevalence",
            trim = T,
            panel.labs = list( met_syn = c("controls", "MetS")),
            panel.labs.background = list(fill = "white")) +
  theme(legend.position = "",
        aspect.ratio = 1,
        panel.grid.major.x = element_blank()) +
  scale_x_discrete(labels = c("<10%", "10-30%", ">30%")) +
  scale_y_continuous(labels = scales::label_percent())
```

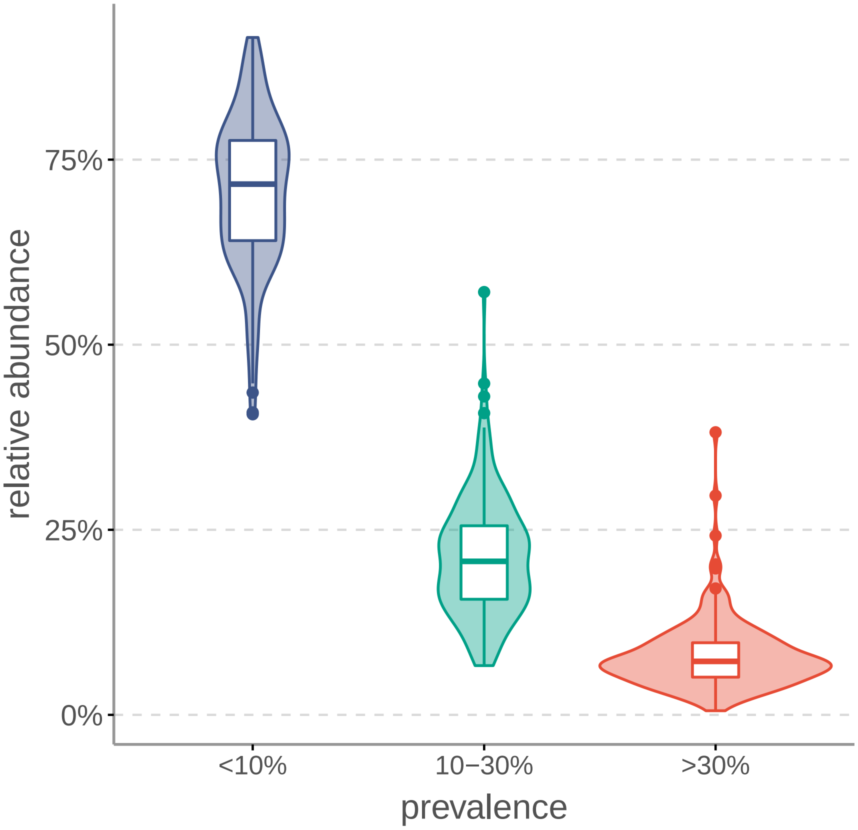

Supplementary Figure 2d: Relative abundance of VCs by their prevalence in VLP samples

```
relative.VLP.psmelt %>%
  left_join(cluster_presence.VLP[,c(1,4)], by = "OTU") %>%
```

```r
mutate(category = gsub("individual-specific", "<10", category)) %>%
group_by(Sample, category) %>%
summarise(Abundance = sum(Abundance)) %>%
ungroup() %>%
mutate(category = factor(category, levels = c("<10", "10-30", ">30"))) %>%
ggviolin(x = "category",
         y = "Abundance",
         color = "category",
         fill = "category",
         palette = category.colors,
         alpha = 0.4,
         add = "boxplot",
         add.params = list(fill = "white"),
         ggtheme = PAdJ_theme(),
         ylab = "relative abundance",
         xlab = "prevalence",
         trim = T,
         panel.labs = list( met_syn = c("controls", "MetS")),
         panel.labs.background = list(fill = "white")) +
theme(legend.position = "",
      aspect.ratio = 1,
      panel.grid.major.x = element_blank()) +
scale_x_discrete(labels = c("<10%", "10-30%", ">30%")) +
scale_y_continuous(labels = scales::label_percent())
```

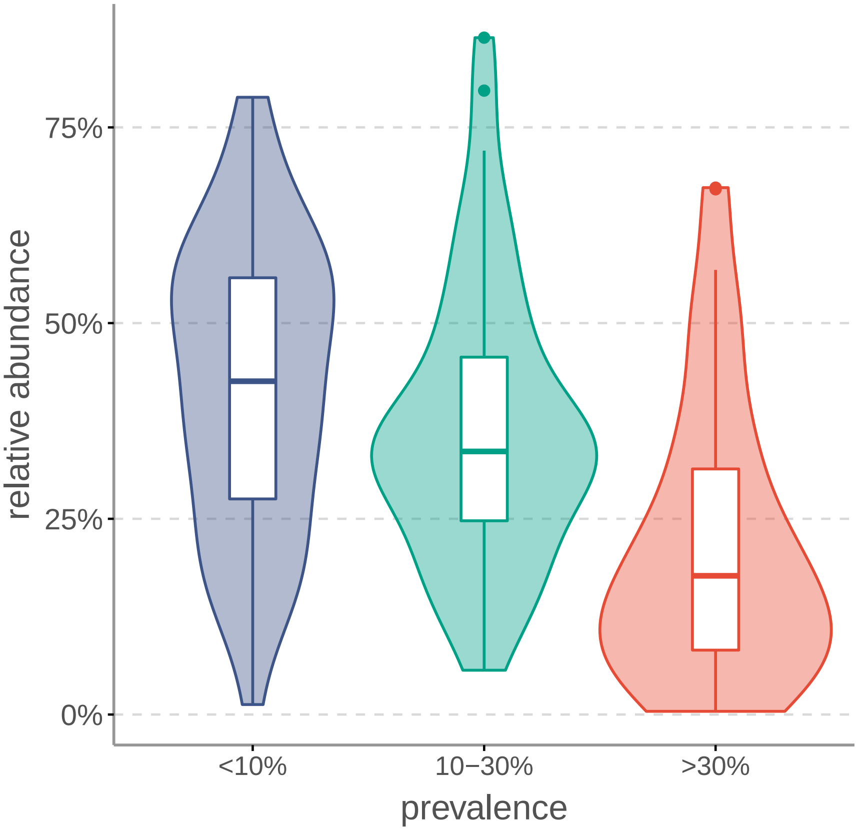

Supplementary Figure 3: viral abundance

Supplementary figure 3a: total viral abundance in bulk samples

```
raw_reads %>%
  filter(datatype == "WGS") %>%
  group_by(samplename) %>%
  summarise(mapped = sum(mapped)) %>%
  ungroup() %>%
  inner_join(total_reads[total_reads$datatype == "WGS",],
             by = c("samplename"="sample")) %>%
  inner_join(metadata[,c(10, 8)], by ="samplename") %>%
  mutate(viral_fraction = mapped/reads) %>%
  MetS_violin_box(y = "viral_fraction", ylab = "reads mapped to viral sequences") +
  scale_y_continuous(labels = scales::label_percent())
```

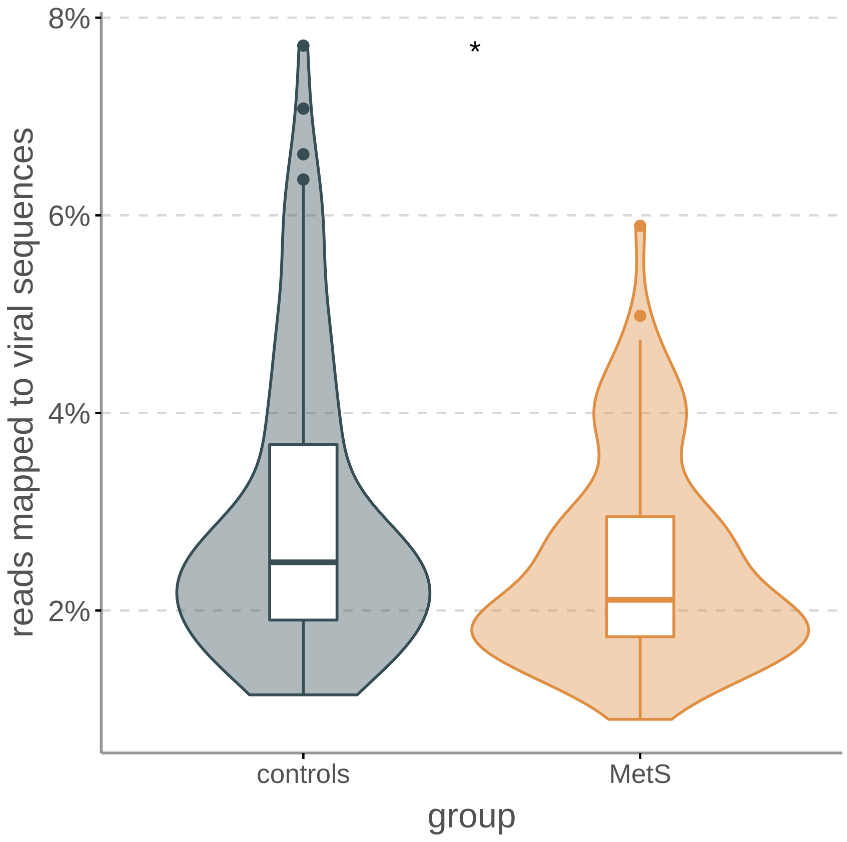

Supplementary figure 3b: cumulative abundance in bulk samples

```
raw_reads %>%
  filter(datatype == "WGS") %>%
  inner_join(VCs[,-3], by = "contigName") %>%
  group_by(samplename) %>%
  mutate(M = sum(mapped)/1e6) %>%
  ungroup() %>%
  group_by(samplename, VC_Subcluster) %>%
```

```
  summarise(RPKM = sum(mapped)/(sum(contigLen/1000)*M)) %>%
  ungroup() %>%
  distinct() %>%
  group_by(samplename) %>%
  summarise(TPM = RPKM/sum(RPKM)) %>%
  ungroup() -> TPM_table.bulk


TPM_table.bulk %>%
  group_by(samplename) %>%
  arrange(-TPM) %>%
  summarise(TPM_cumsum = cumsum(TPM),
            rank = row_number()) %>%
  ungroup() %>%
  pivot_wider(names_from = rank, values_from = TPM_cumsum, values_fill = 1) %>%
  pivot_longer(-1, names_to = "rank", values_to = "TPM_cumsum") %>%
  left_join(metadata, by = "samplename") %>%
  ggplot(aes(x = as.numeric(rank),
             y = TPM_cumsum,
             color = met_syn,
             fill = met_syn)) +
  stat_summary(fun = mean, geom = "line") +
  stat_summary(fun.data = "mean_se", geom = "ribbon",
               alpha = 0.25) +
  PAdJ_theme() +
  theme(aspect.ratio = 1) +
  scale_color_manual(values = MetS.colors) +
  scale_fill_manual(values = MetS.colors) +
  scale_x_continuous(name = "number of VCs") +
  scale_y_continuous(name = "cumulative rel. abundance",
                     labels = scales::label_percent())
```

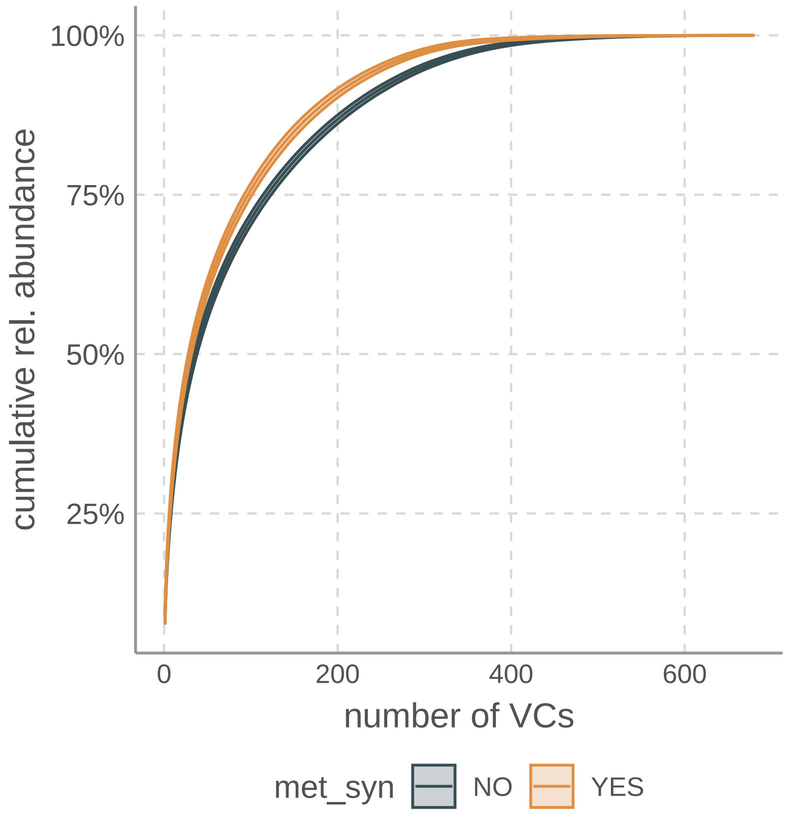

Supplementary figure 3c: total viral abundance in VLP samples

```
raw_reads %>%
  filter(datatype == "VLP") %>%
  group_by(samplename) %>%
  summarise(mapped = sum(mapped)) %>%
  ungroup() %>%
  inner_join(total_reads[total_reads$datatype == "VLP",],
             by = c("samplename"="sample")) %>%
  inner_join(metadata[,c(10, 8)], by ="samplename") %>%
  mutate(viral_fraction = mapped/reads) %>%
  MetS_violin_box(y = "viral_fraction", ylab = "reads mapped to viral sequences") +
  scale_y_continuous(labels = scales::label_percent())
```

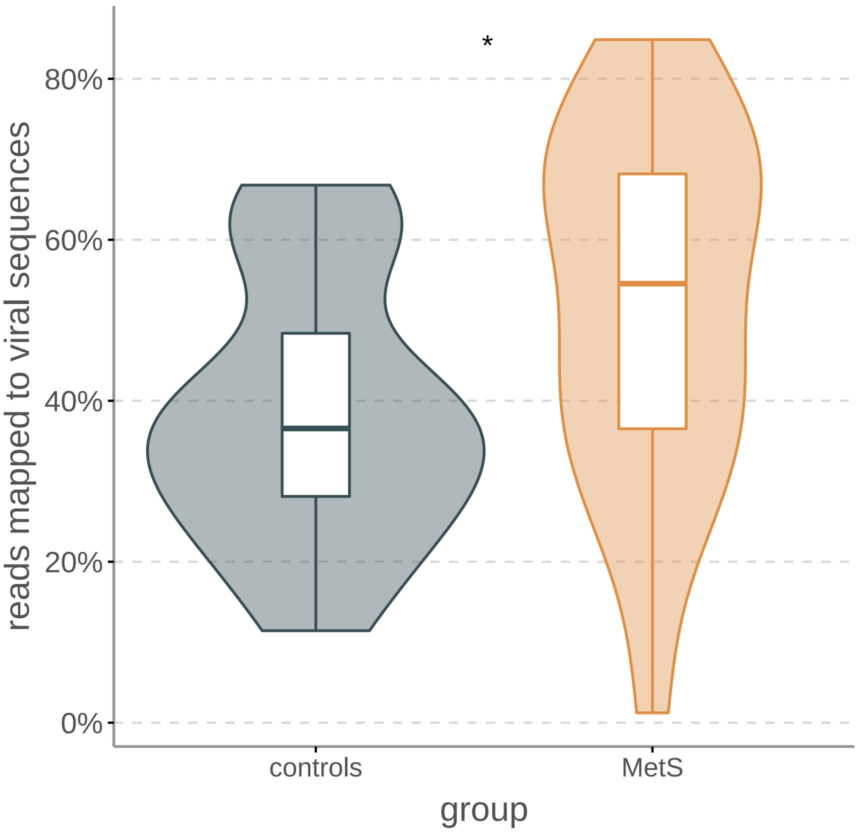

Supplementary figure 3d: cumulative abundance in VLP samples

```
raw_reads %>%
  filter(datatype == "VLP") %>%
  inner_join(VCs[,-3], by = "contigName") %>%
  group_by(samplename) %>%
  mutate(M = sum(mapped)/1e6) %>%
  ungroup() %>%
  group_by(samplename, VC_Subcluster) %>%
  summarise(RPKM = sum(mapped)/(sum(contigLen/1000)*M)) %>%
  ungroup() %>%
  distinct() %>%
  group_by(samplename) %>%
  summarise(TPM = RPKM/sum(RPKM)) %>%
  ungroup() -> TPM_table.VLP

TPM_table.VLP %>%
  group_by(samplename) %>%
  arrange(-TPM) %>%
  summarise(TPM_cumsum = cumsum(TPM),
            rank = row_number()) %>%
  ungroup() %>%
  pivot_wider(names_from = rank, values_from = TPM_cumsum, values_fill = 1) %>%
  pivot_longer(-1, names_to = "rank", values_to = "TPM_cumsum") %>%
```

```
left_join(metadata, by = "samplename") %>%
ggplot(aes(x = as.numeric(rank),
           y = TPM_cumsum,
           color = met_syn,
           fill = met_syn)) +
stat_summary(fun = mean, geom = "line") +
stat_summary(fun.data = "mean_se", geom = "ribbon",
             alpha = 0.25) +
PAdJ_theme() +
theme(aspect.ratio = 1) +
scale_color_manual(values = MetS.colors) +
scale_fill_manual(values = MetS.colors) +
scale_x_continuous(name = "number of VCs") +
scale_y_continuous(name = "cumulative rel. abundance",
                   labels = scales::label_percent())
```

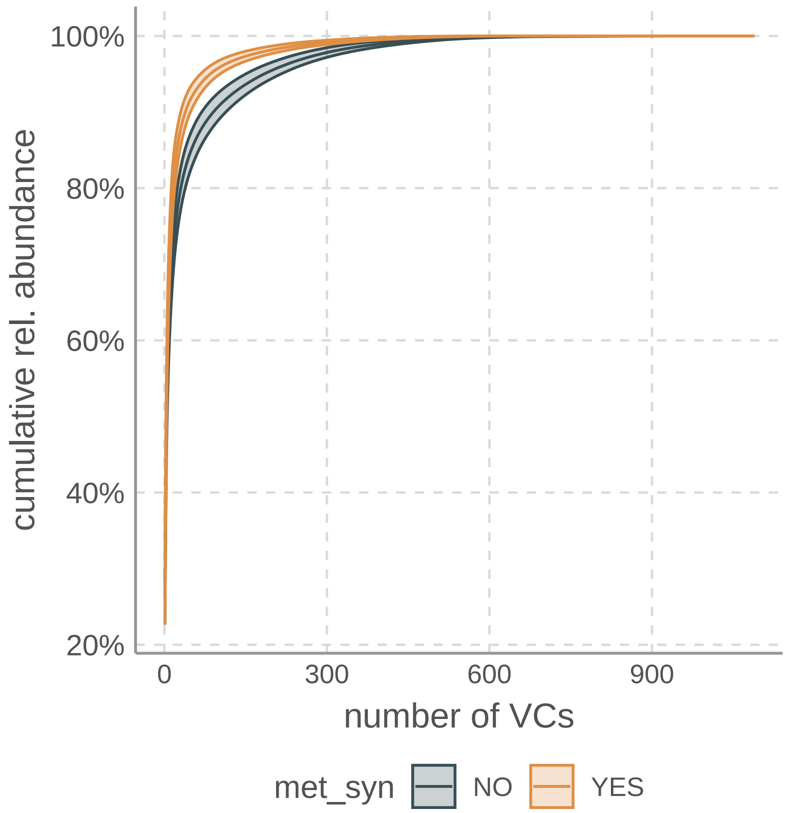

Supplementary Figure 4: bacterial 16s rRNA amplicon sequencing analysis

Supplementary Figure 4a: Bacterial richness

```
richness(x = raw.16s.ps, "Chao1") %>%
  rownames_to_column("samplename") %>%
  mutate(samplename = paste("S", samplename, sep = "")) %>%
  left_join(metadata, by = "samplename") %>%
  MetS_violin_box(y = "chao1", ylab = "Chao1 richness")
```

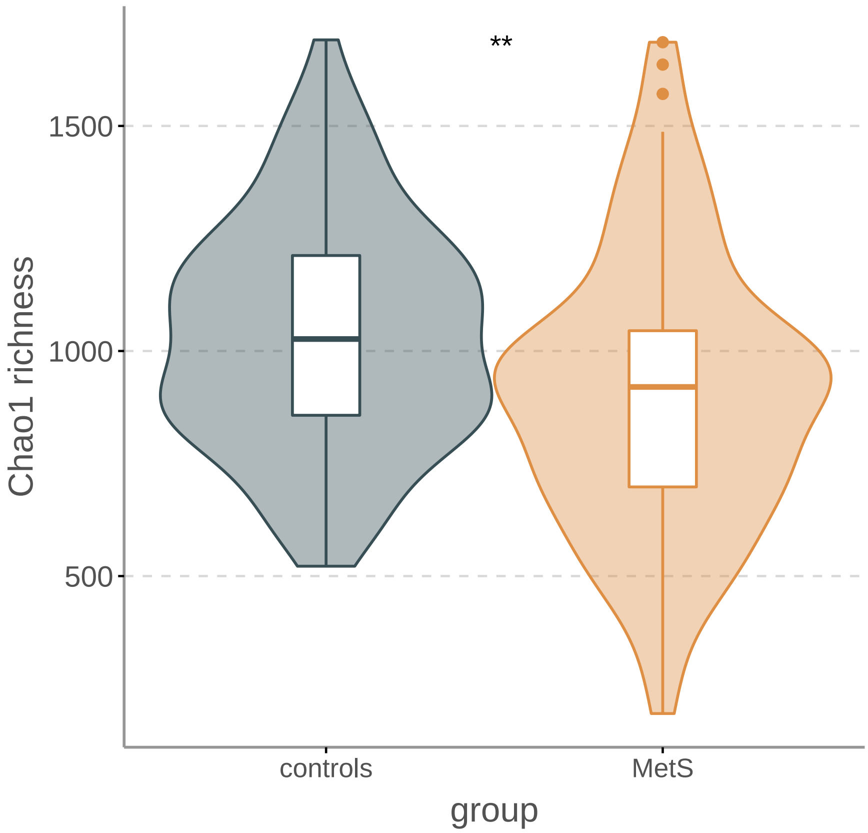

Supplementary Figure 4b: Bacterial evenness

```
evenness(x = raw.16s.ps, "pielou") %>%
  rownames_to_column("samplename") %>%
  mutate(samplename = paste("S", samplename, sep = "")) %>%
  left_join(metadata, by = "samplename") %>%
  MetS_violin_box(y = "pielou", ylab = "pielou evenness")
```

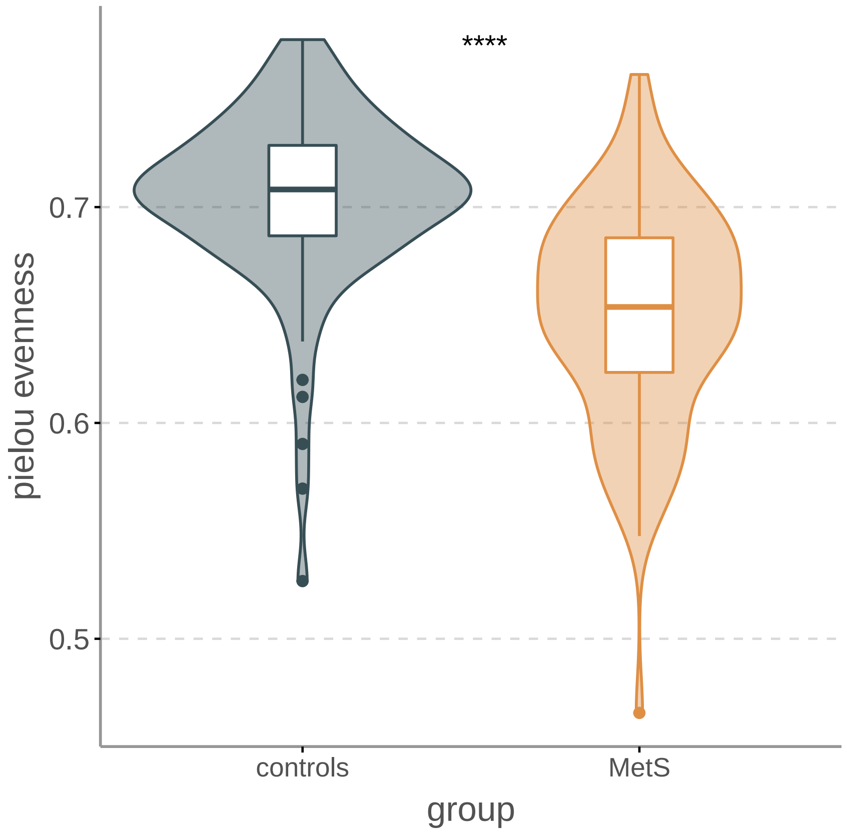

Supplementary Figure 4c: Bacterial alpha diversity

```
diversity(x = raw.16s.ps, "shannon") %>%
  rownames_to_column("samplename") %>%
  mutate(samplename = paste("S", samplename, sep = "")) %>%
  left_join(metadata, by = "samplename") %>%
  MetS_violin_box(y = "shannon", ylab = "Shannon H'")
```

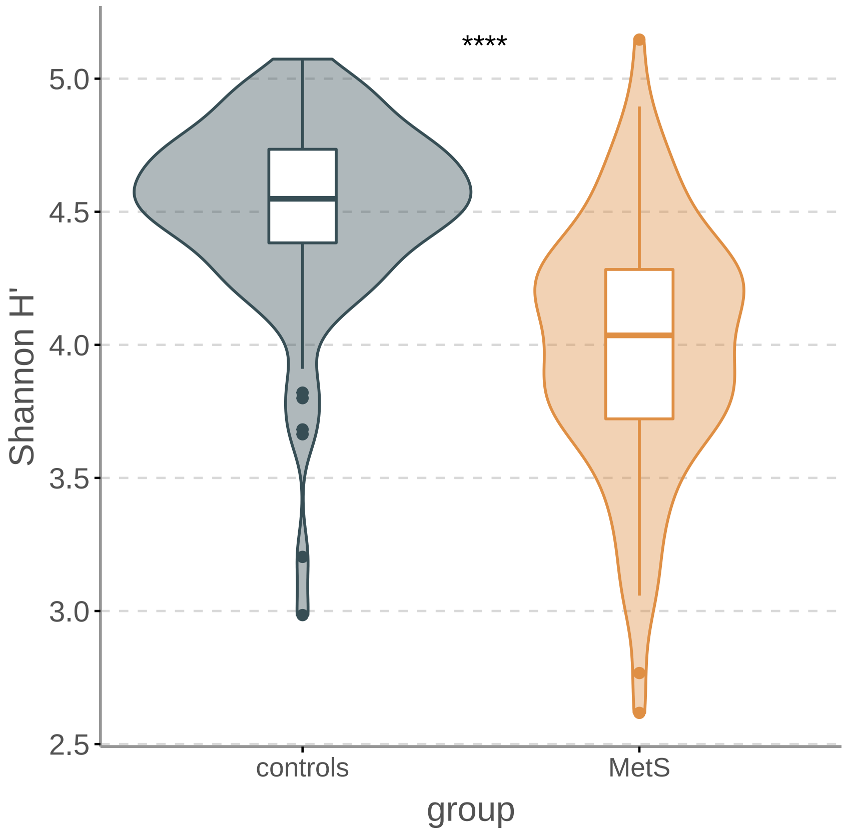

bacterial beta diversity — ***PERMANOVA***

```
BC_bac <- phyloseq::distance(relative.16s.ps, method = "bray")
set.seed(130499)
adonis2(BC_bac ~ roken + geslacht + leeftijd + alcohol + metformin + met_syn,
        data = data.frame(relative.16s.ps@sam_data))
```

```
## Permutation test for adonis under reduced model
## Terms added sequentially (first to last)
## Permutation: free
## Number of permutations: 999
##
## adonis2(formula = BC_bac ~ roken + geslacht + leeftijd + alcohol + metformin + met_syn, data = data.
##            Df SumOfSqs      R2       F Pr(>F)
## roken       1    0.375 0.00681  1.4478  0.043 *
## geslacht    1    0.489 0.00889  1.8914  0.003 **
## leeftijd    1    0.569 0.01034  2.1992  0.001 ***
## alcohol     1    0.438 0.00796  1.6930  0.013 *
## metformin   1    0.825 0.01499  3.1892  0.001 ***
## met_syn     1    3.437 0.06244 13.2817  0.001 ***
## Residual  189   48.902 0.88857
## Total     195   55.035 1.00000
## ---
## Signif. codes:  0 '***' 0.001 '**' 0.01 '*' 0.05 '.' 0.1 ' ' 1
```

```
set.seed(130499)
adonis_out <- adonis2(BC_bac ~ roken + geslacht + leeftijd + alcohol + metformin + met_syn,
       data = data.frame(relative.16s.ps@sam_data))
```

Supplementary Figure 4d: bacterial beta diversity — *PCoA*

```
PCoA.bac <- ordinate(relative.16s.ps, method = "PCoA", distance = "bray")

eigen <- PCoA.bac$values[c(1,2),2]

as.data.frame(PCoA.bac$vectors[,c(1,2)]) %>%
  rownames_to_column("heliusnr") %>%
  left_join(metadata[,-10] %>% mutate(heliusnr = as.character(heliusnr)),
            by = "heliusnr") %>%
  ggscatter(x = "Axis.1",
            y = "Axis.2",
            color = "met_syn",
            palette = MetS.colors,
            star.plot = T,
            star.plot.lty = "dotted",
            mean.point = T,
            mean.point.size = 3,
            ggtheme = PAdJ_theme(),
            size = .75) +
  theme(aspect.ratio = 1,
        panel.border = element_rect(color = "#969696"),
        axis.text.y = element_text(size=10)) +
  annotate("text",
           label = paste("Permanova p =", adonis_out$`Pr(>F)`[6]),
           x = -0.12,
           y = 0.3,
           color = "grey30",
           size = 5) +
  scale_x_continuous(name = paste("Axis 1 (", round(eigen[1]*100, 1), "%)", sep = "")) +
  scale_y_continuous(name = paste("Axis 2 (", round(eigen[2]*100, 1), "%)", sep = ""))
```

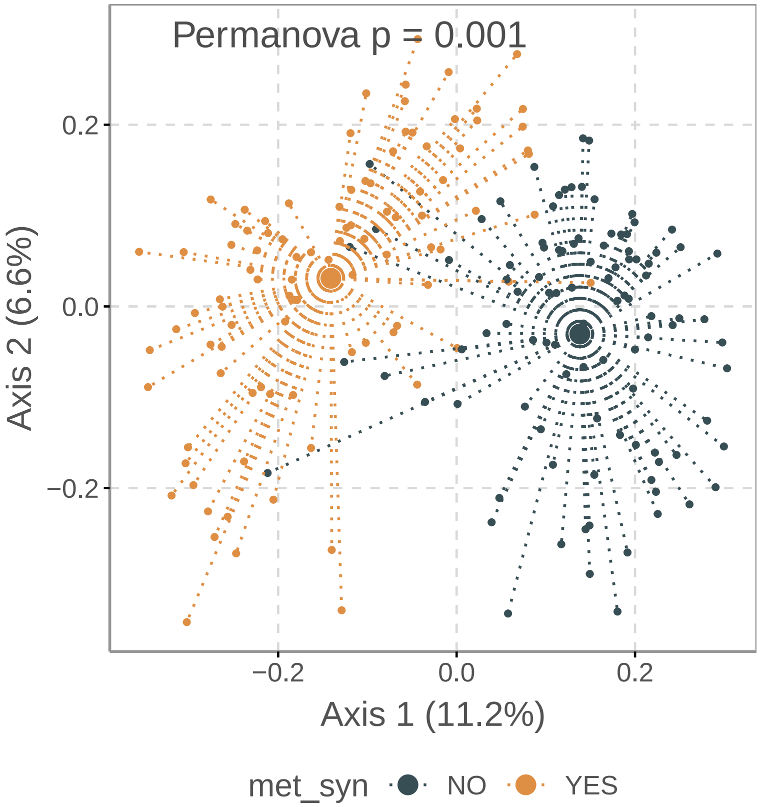

Supplementary Figure 5: individual correlations between richness/evenness and clinical MetS parameters.

```
WGS_Bacterial_richness %>%
  inner_join(clinical_data) %>%
  pivot_longer(4:8,
               names_to = "parameter",
               values_to = "value") %>%
  MetS_scatter(x = "value",
               y = "richness",
               ylab = "richness",
               xlab = "value") +
    facet_wrap(vars(parameter),
               scales = "free_x",
               ncol = 5) +
  theme(strip.background = element_rect(fill = "white"),
        strip.text = element_text(size = 8),
        aspect.ratio = 1)
```

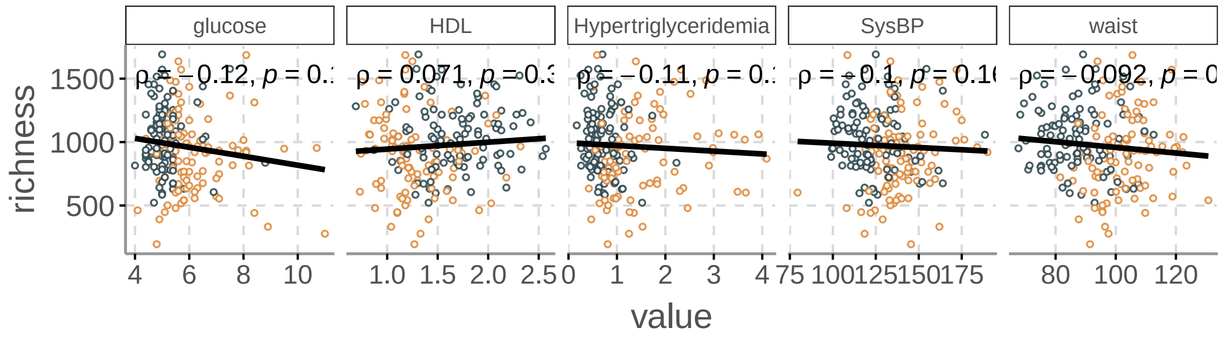


```
WGS_Viral_richness %>%
  inner_join(clinical_data) %>%
  pivot_longer(4:8,
               names_to = "parameter",
               values_to = "value") %>%
  MetS_scatter(x = "value",
               y = "richness",
               ylab = "richness",
               xlab = "value") +
  facet_wrap(vars(parameter),
             scales = "free_x",
             ncol = 5) +
  theme(strip.background = element_rect(fill = "white"),
        strip.text = element_text(size = 8),
        aspect.ratio = 1)
```

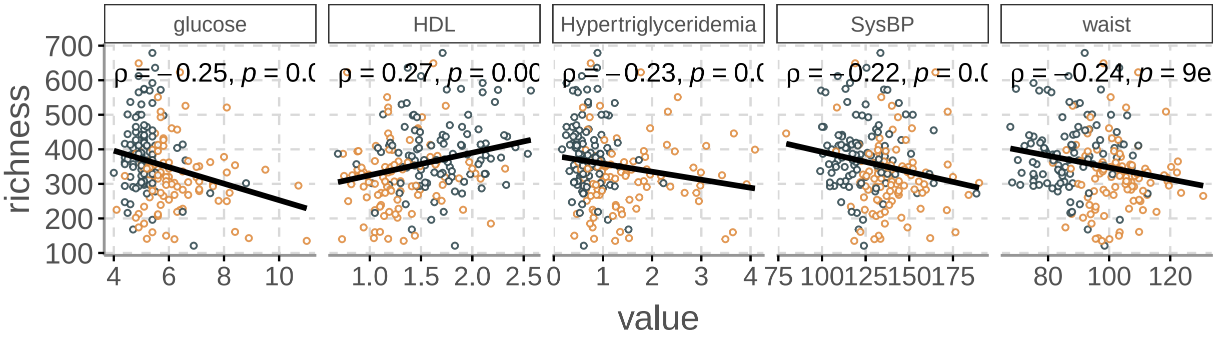


```
WGS_Bacterial_evenness %>%
  inner_join(clinical_data) %>%
  pivot_longer(4:8,
               names_to = "parameter",
               values_to = "value") %>%
  MetS_scatter(x = "value",
               y = "pielou",
               ylab = "pielou",
               xlab = "value") +
  facet_wrap(vars(parameter),
             scales = "free_x",
             ncol = 5) +
  theme(strip.background = element_rect(fill = "white"),
        strip.text = element_text(size = 8),
        aspect.ratio = 1)
```

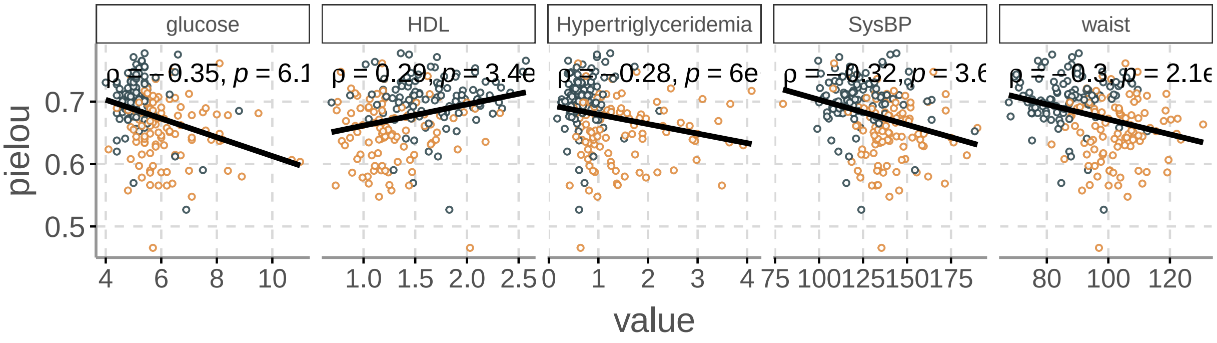


```
WGS_Viral_evenness %>%
  inner_join(clinical_data) %>%
  pivot_longer(4:8,
               names_to = "parameter",
               values_to = "value") %>%
  MetS_scatter(x = "value",
               y = "pielou",
               ylab = "pielou",
               xlab = "value") +
  facet_wrap(vars(parameter),
             scales = "free_x",
             ncol = 5) +
  theme(strip.background = element_rect(fill = "white"),
        strip.text = element_text(size = 8),
        aspect.ratio = 1)
```

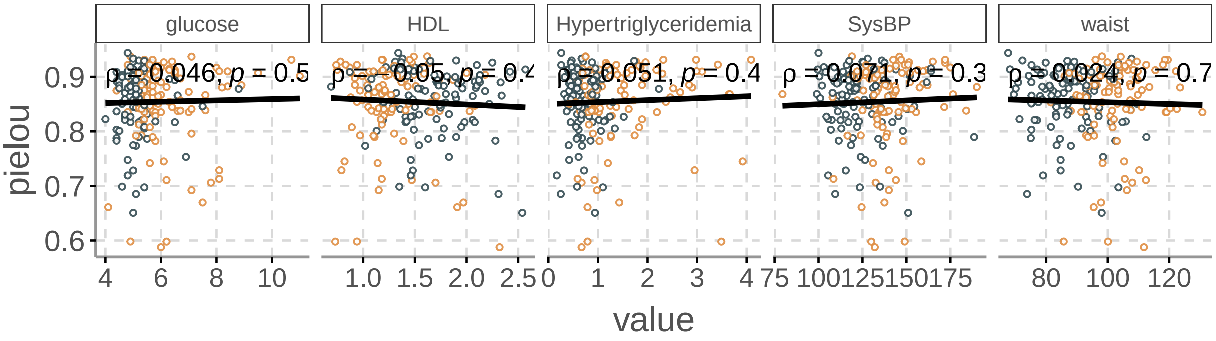

Supplementary Figure 6: ANCOM-BC analysis on the most common bacterial families

```
bacterial_ANCOM <- subset_taxa(raw.16s.ps, gsub("_.+$", "", Family) %in%
                                  most_common_hosts$family)
bacterial_ANCOM <- phyloseq::tax_glom(bacterial_ANCOM, "Family", NArm = TRUE)
taxa_names(bacterial_ANCOM) <- tax_table(bacterial_ANCOM)[,"Family"]

out_bac <- ancombc(bacterial_ANCOM, group = "met_syn", p_adj_method = "BH",
                   zero_cut = 0.8, lib_cut = 0, struc_zero = T, neg_lb = F,
                   tol = 1e-5, max_iter = 100, conserve = T, alpha = 0.001, global = T,
                   formula = "roken + leeftijd + geslacht + alcohol + metformin + met_syn")

bac_ANCOM <- tibble(species = row.names(as.data.frame(out_bac$res$beta)),
                    logFoldChange = as.data.frame(out_bac$res$beta)$met_synYES,
                    se = as.data.frame(out_bac$res$se)$met_synYES,
                    q_val = as.data.frame(out_bac$res$q_val)$met_synYES,
                    met_syn = ifelse(logFoldChange > 0, "YES", "NO"))

bac_ANCOM %>%
  ggdotchart(x = "species",
             y = "logFoldChange",
             color = "met_syn",
```

```
            rotate = T,
            ggtheme = PAdJ_theme(),
            sorting = "descending",
            size = 1,
            dot.size = 3,
            xlab = "VC",
            ylab = "log fold change") +
geom_linerange(aes(ymin = logFoldChange-se,
                   ymax = logFoldChange+se,
                   color = met_syn),
                 position=position_dodge(.9)) +
gghighlight::gghighlight(q_val <= 0.05, use_direct_label = F,
                         unhighlighted_params = list(colour = NULL, shape = 21)) +
theme(axis.text.y = element_text(face = "italic"),
      legend.position = "") +
geom_hline(yintercept = 0,
            color = "tomato1",
            linetype = "dashed",
            size = 0.5) +
scale_color_manual(values = MetS.colors,
                   name = "differentially abundant (q < 0.05)",
                   labels = c("controls", "MetS")) +
scale_y_continuous(limits = c(-2.5,2.5))
```

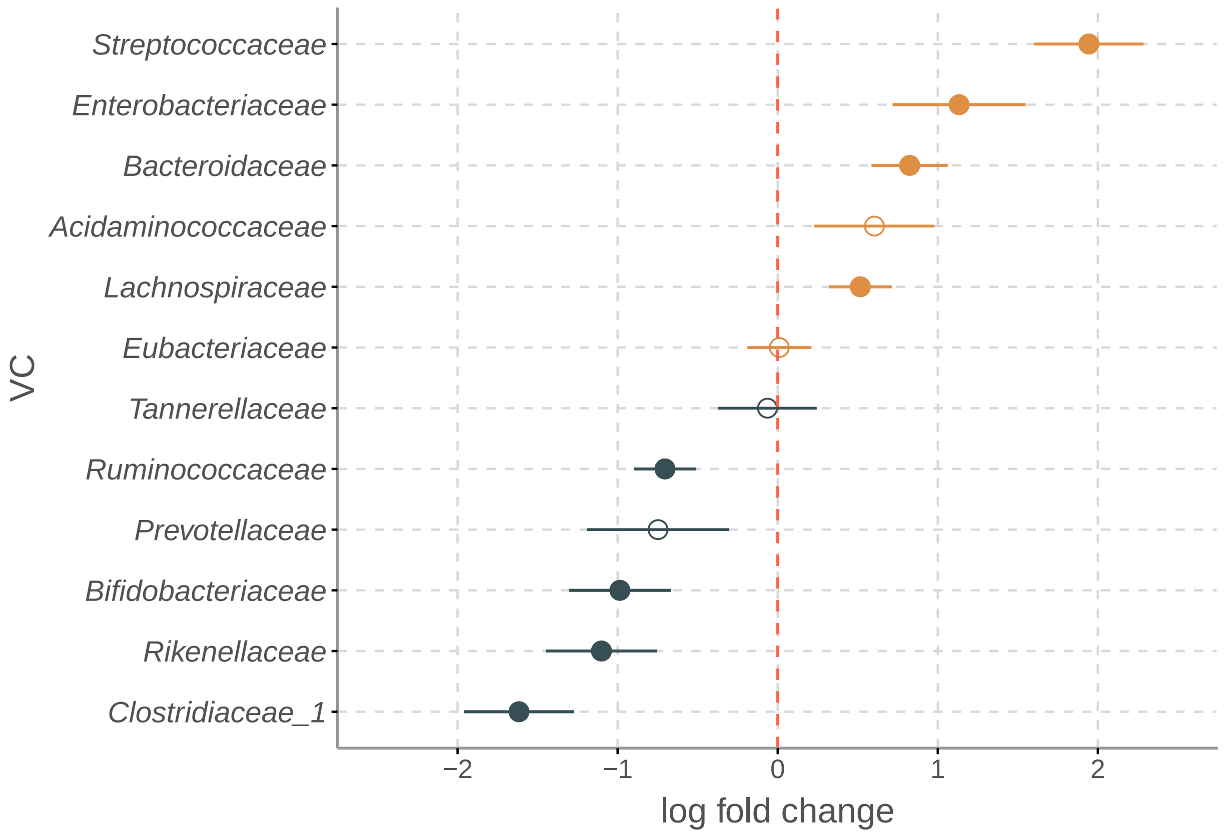

Supplementary Figure 8: Prevalence of Ca. Heliusviridae phages in two validation cohorts

```
heliusviridae %>%
  inner_join(VCs %>% mutate(contigName = gsub("_S.+$", "", contigName)),
             by = "contigName") %>%
  select(VC_Subcluster, group) %>%
  distinct() %>%
  inner_join(relative.bulk.psmelt[,c(1:3, 10)], by = c("VC_Subcluster"="OTU")) %>%
  group_by(Sample) %>%
  summarise(rel_abundance = sum(Abundance)) %>%
  ungroup() %>%
  filter(!is.na(Sample)) %>%
  mutate(present = ifelse(rel_abundance > 0,
                          "present",
                          "absent")) %>%
  group_by(present) %>%
  summarise(participants = n_distinct(Sample)) %>%
  ungroup() %>%
  mutate(study = "this study") -> helius_numbers_1


rio::import("validation/T2D_read_counts.csv") %>%
  group_by(sample) %>%
  summarise(total_reads = sum(mapped)) %>%
  ungroup() %>%
  mutate(present = ifelse(total_reads > 0,
                          "present",
                          "absent")) %>%
  group_by(present) %>%
  summarise(participants = n_distinct(sample)) %>%
  ungroup() %>%
  mutate(study = "Ma, et al (T2D)") -> helius_numbers_2

rio::import("validation/hyper_read_counts.csv") %>%
  group_by(sample) %>%
  summarise(total_reads = sum(mapped)) %>%
  ungroup() %>%
  mutate(present = ifelse(total_reads > 0,
                          "present",
                          "absent")) %>%
  group_by(present) %>%
  summarise(participants = n_distinct(sample)) %>%
  ungroup() %>%
  mutate(study = "Han, et al (hypertension)") -> helius_numbers_3

rbind(helius_numbers_1, helius_numbers_2, helius_numbers_3) %>%
  ggplot(aes(x = study ,
             y = participants,
             color = present,
             fill = present)) +
  geom_bar(stat = "identity",
```

```
          position = position_stack(),
          alpha = 0.5,
          size = 1,
          width = 0.5) +
  PAdJ_theme() +
  theme(legend.position = "top",
        panel.grid.major.y = element_blank(),
        strip.background = element_rect(fill = "white"),
        axis.line.x = element_blank(),
        axis.line.y = element_blank()) +
  scale_color_manual(name = expression(italic("heliusviridae")),
                     values = c("grey80", "forestgreen")) +
  scale_fill_manual(name = expression(italic("heliusviridae")),
                    values = c("grey80", "forestgreen")) +
  scale_x_discrete(name = "study",
                   expand = c(0.2,0),
                   labels = c(expression("Han,"~italic("et al")~"(hypertension)"),
                              expression("Ma,"~italic("et al")~"(T2D)"),
                              "This study")) +
  scale_y_continuous(name = "number of participants",
                     expand = c(0,0),
                     limits = c(0,205)) +
  coord_flip()
```

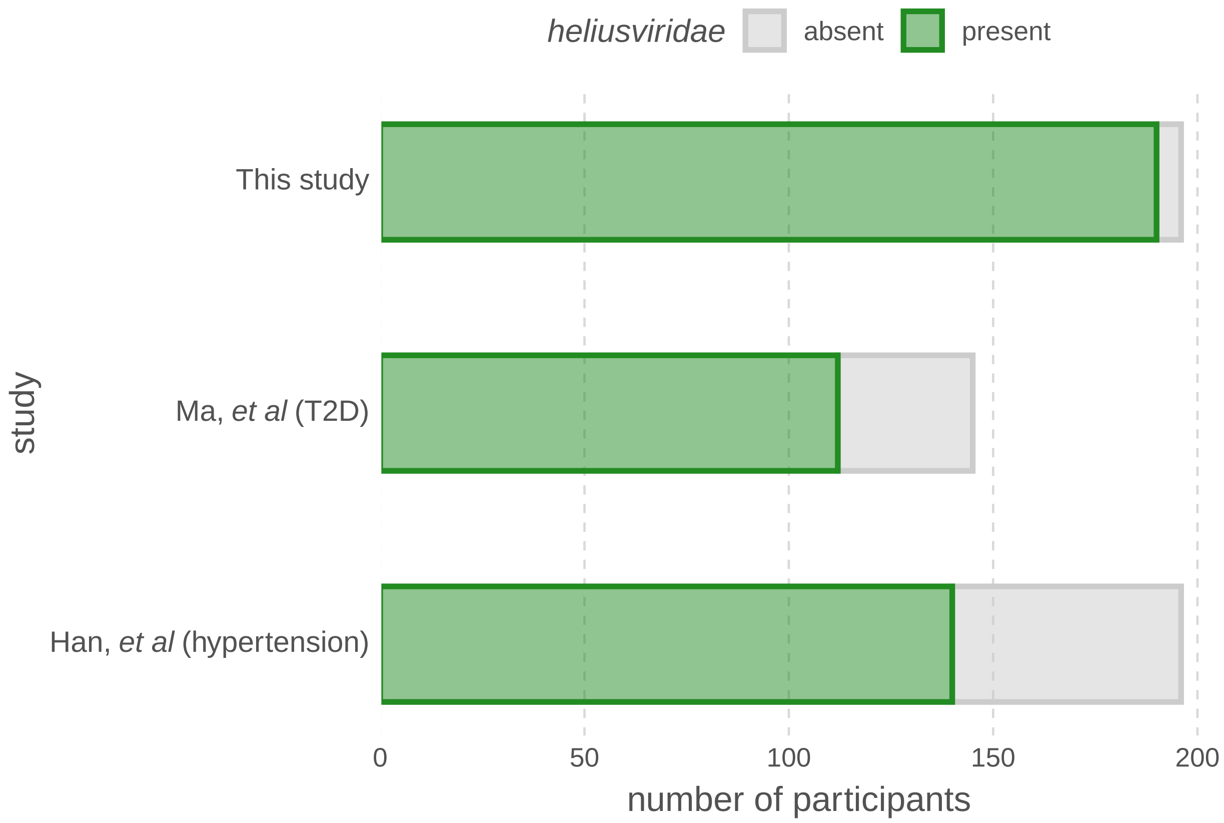

```r
sessionInfo()
```

```
## R version 4.1.1 (2021-08-10)
## Platform: x86_64-pc-linux-gnu (64-bit)
## Running under: Ubuntu 20.04.3 LTS
##
## Matrix products: default
## BLAS:   /usr/lib/x86_64-linux-gnu/blas/libblas.so.3.9.0
## LAPACK: /usr/lib/x86_64-linux-gnu/lapack/liblapack.so.3.9.0
##
## locale:
##  [1] LC_CTYPE=en_US.UTF-8       LC_NUMERIC=C
##  [3] LC_TIME=nl_NL.UTF-8        LC_COLLATE=en_US.UTF-8
##  [5] LC_MONETARY=nl_NL.UTF-8    LC_MESSAGES=en_US.UTF-8
##  [7] LC_PAPER=nl_NL.UTF-8       LC_NAME=C
##  [9] LC_ADDRESS=C               LC_TELEPHONE=C
## [11] LC_MEASUREMENT=nl_NL.UTF-8 LC_IDENTIFICATION=C
##
## attached base packages:
## [1] stats4    parallel  stats     graphics  grDevices utils     datasets
## [8] methods   base
##
## other attached packages:
##  [1] RColorBrewer_1.1-3  gplots_3.1.3        ANCOMBC_1.2.2
##  [4] microbiome_1.14.0   phyloseq_1.36.0     Biostrings_2.60.2
##  [7] GenomeInfoDb_1.28.4 XVector_0.32.0      IRanges_2.26.0
## [10] S4Vectors_0.30.2    BiocGenerics_0.38.0 vegan_2.6-2
## [13] lattice_0.20-45     permute_0.9-7       ggpubr_0.4.0
## [16] viridis_0.6.2       viridisLite_0.4.0   forcats_0.5.1
## [19] stringr_1.4.0       dplyr_1.0.9         purrr_0.3.4
## [22] readr_2.1.2         tidyr_1.2.0         tibble_3.1.7
## [25] ggplot2_3.3.6       tidyverse_1.3.1
##
## loaded via a namespace (and not attached):
##   [1] Rtsne_0.16             colorspace_2.0-3       ggsignif_0.6.3
##   [4] rio_0.5.29             ellipsis_0.3.2         gghighlight_0.3.2
##   [7] fs_1.5.2               rstudioapi_0.13        farver_2.1.0
##  [10] ggrepel_0.9.1          fansi_1.0.3            lubridate_1.8.0
##  [13] xml2_1.3.3             codetools_0.2-18       splines_4.1.1
##  [16] knitr_1.39             ade4_1.7-19            jsonlite_1.8.0
##  [19] nloptr_2.0.1           broom_0.8.0            cluster_2.1.2
##  [22] dbplyr_2.1.1           pheatmap_1.0.12        compiler_4.1.1
##  [25] httr_1.4.3             backports_1.4.1        assertthat_0.2.1
##  [28] Matrix_1.3-4           fastmap_1.1.0          cli_3.3.0
##  [31] htmltools_0.5.2        tools_4.1.1            igraph_1.3.1
##  [34] gtable_0.3.0           glue_1.6.2             GenomeInfoDbData_1.2.6
##  [37] reshape2_1.4.4         Rcpp_1.0.8.3           carData_3.0-5
##  [40] Biobase_2.52.0         cellranger_1.1.0       vctrs_0.4.1
##  [43] rhdf5filters_1.4.0     multtest_2.48.0        ape_5.6-2
##  [46] nlme_3.1-152           iterators_1.0.14       xfun_0.30
##  [49] rbibutils_2.2.8        openxlsx_4.2.5         rvest_1.0.2
##  [52] lifecycle_1.0.1        gtools_3.9.2           rstatix_0.7.0
##  [55] zlibbioc_1.38.0        MASS_7.3-54            scales_1.2.0
```

```
##  [58] hms_1.1.1             NbClust_3.0.1         biomformat_1.20.0
##  [61] rhdf5_2.36.0          curl_4.3.2            yaml_2.3.5
##  [64] gridExtra_2.3         stringi_1.7.6         highr_0.9
##  [67] foreach_1.5.2         caTools_1.18.2        zip_2.2.0
##  [70] Rdpack_2.3            rlang_1.0.2           pkgconfig_2.0.3
##  [73] bitops_1.0-7          evaluate_0.15         Rhdf5lib_1.14.2
##  [76] labeling_0.4.2        tidyselect_1.1.2      ggsci_2.9
##  [79] plyr_1.8.7            magrittr_2.0.3        R6_2.5.1
##  [82] generics_0.1.2        DBI_1.1.2             foreign_0.8-81
##  [85] pillar_1.7.0          haven_2.5.0           withr_2.5.0
##  [88] mgcv_1.8-37           survival_3.2-13       abind_1.4-5
##  [91] RCurl_1.98-1.6        modelr_0.1.8          crayon_1.5.1
##  [94] car_3.0-13            KernSmooth_2.23-20    utf8_1.2.2
##  [97] tzdb_0.3.0            rmarkdown_2.14        grid_4.1.1
## [100] readxl_1.4.0          data.table_1.14.2     reprex_2.0.1
## [103] digest_0.6.29         munsell_0.5.0
```
