# Supplementary material for: Gut virome profiling identifies a widespread bacteriophage family associated with metabolic syndrome
Source: Nat Commun. 2022 Jun 23;13:3594. doi: 10.1038/s41467-022-31390-5 (PMC9226167; doi:10.1038/s41467-022-31390-5)
Supplement: Supplementary file 11 — Reporting Summary [file 41467_2022_31390_MOESM11_ESM.pdf]

nature portfolio

Corresponding author(s): Dr. Hilde Herrema

Last updated by author(s): 30/05/2022

Reporting Summary

Nature Portfolio wishes to improve the reproducibility of the work that we publish. This form provides structure for consistency and transparency in reporting. For further information on Nature Portfolio policies, see our Editorial Policies and the Editorial Policy Checklist.

Statistics

For all statistical analyses, confirm that the following items are present in the figure legend, table legend, main text, or Methods section.

n/a	Confirmed	
☐	☒	The exact sample size (*n*) for each experimental group/condition, given as a discrete number and unit of measurement
☐	☒	A statement on whether measurements were taken from distinct samples or whether the same sample was measured repeatedly
☐	☒	The statistical test(s) used AND whether they are one- or two-sided<br>*Only common tests should be described solely by name; describe more complex techniques in the Methods section.*
☐	☒	A description of all covariates tested
☐	☒	A description of any assumptions or corrections, such as tests of normality and adjustment for multiple comparisons
☐	☒	A full description of the statistical parameters including central tendency (e.g. means) or other basic estimates (e.g. regression coefficient) AND variation (e.g. standard deviation) or associated estimates of uncertainty (e.g. confidence intervals)
☐	☒	For null hypothesis testing, the test statistic (e.g. *F*, *t*, *r*) with confidence intervals, effect sizes, degrees of freedom and *P* value noted<br>*Give P values as exact values whenever suitable.*
☒	☐	For Bayesian analysis, information on the choice of priors and Markov chain Monte Carlo settings
☒	☐	For hierarchical and complex designs, identification of the appropriate level for tests and full reporting of outcomes
☒	☐	Estimates of effect sizes (e.g. Cohen's *d*, Pearson's *r*), indicating how they were calculated

Our web collection on statistics for biologists contains articles on many of the points above.

Software and code

Policy information about availability of computer code

Data collection	No software was used for data collection.
Data analysis	R 4.1.0, fastp v0.23.1, bowtie2 v2.4.0, metaSPAdes v3.14.1, Biostrings v3.12, virSorter v1.0.6, VirFinder v1.1, CAT v5.1.2, BBTools v38.84, Prodigal v2.6.2, Contact2 v0.9.18, CheckV v 0.7.0-1, metaphlan v3.0.13, coverM filter v0.5.0, samtools idxstats v1.10, bedtools genomecov v2.29.2, ggpubr v0.4.0, ggplot2 v3.3.2, phyloseq v1.33.0, microbiome v1.11.2, vegan v2.6-2, CRISPRdetect v2.4, BLASTn v2.12.0+, ANCOM-BC v1.0.2 , BLAST v2.9.0+, Easyfig v2.2.5, Diamond v2.0.4, PROKKA v1.14.6, InterProScan v5.48-83.0, Clustal Omega v1.2.4, trimAl v1.4.rev15, IQ-Tree v2.0.3,

For manuscripts utilizing custom algorithms or software that are central to the research but not yet described in published literature, software must be made available to editors and reviewers. We strongly encourage code deposition in a community repository (e.g. GitHub). See the Nature Portfolio guidelines for submitting code & software for further information.

Data

Policy information about availability of data

All manuscripts must include a data availability statement. This statement should provide the following information, where applicable:

- Accession codes, unique identifiers, or web links for publicly available datasets
- A description of any restrictions on data availability
- For clinical datasets or third party data, please ensure that the statement adheres to our policy

The sequencing data generated in this study have been deposited in the European Genome-Phenome Archive database under accession code EGAS00001006260 [https://ega-archive.org/datasets/EGAD00001008765]. The sequencing data are available under restricted access for restrictions imposed by the signed consent of

participants, access can be obtained by submitting a proposal to the HELIUS Executive Board as outlined at http://www.heliusstudy.nl/en/researchers/collaboration, by email: heliuscoordinator@amsterdamumc.nl. The HELIUS Executive Board will check proposals for compatibility with the general objectives, ethical approvals and informed consent forms of the HELIUS study. There are no other restrictions to obtaining the data and all data requests will be processed in the same manner. The data generated in this study are provided in the Source Data file. The human genome data used in this study is available at the National centre for biotechnology information (NCBI) under accession GRCh37 [https://www.ncbi.nlm.nih.gov/assembly/GCF_000001405.13/]. The CRISPR spacer dataset derived from the PATRIC database is available from Supplementary Table 1 of reference 92 [https://academic.oup.com/nar/article/48/21/12074/5997439#supplementary-data]. The reads from the validation cohorts are available from NCBI under the NCBI BioProject accession numbers PRJNA646512 [https://www.ncbi.nlm.nih.gov/bioproject/PRJNA646512], PRJEB13870 [https://www.ncbi.nlm.nih.gov/bioproject/PRJEB13870], PRJNA422434 [https://www.ncbi.nlm.nih.gov/bioproject/PRJNA422434], and PRJNA573942 [https://www.ncbi.nlm.nih.gov/bioproject/PRJNA573942].

Field-specific reporting

Please select the one below that is the best fit for your research. If you are not sure, read the appropriate sections before making your selection.

☒ Life sciences ☐ Behavioural & social sciences ☐ Ecological, evolutionary & environmental sciences

For a reference copy of the document with all sections, see nature.com/documents/nr-reporting-summary-flat.pdf

Life sciences study design

All studies must disclose on these points even when the disclosure is negative.

Sample size	No sample-size calculation was performed. Sample size was determined according to the availability of faecal matter for metagenomic sequencing among the HELIUS cohort. The sample size of 196 people was high enough to support statistical analysis of the results
Data exclusions	No participants were excluded from the study.
Replication	Replicability of the results was assured by including a large number of participants from diverse backgrounds (half were ethnically Dutch, half were African-Surinamese). Each participant provided a single sample. Analysis of differentially abundant viruses only analysed those present in >10% of participants, which, given the high inter-individual diversity of gut phage populations, can be considered as common phages. Commonality of the Ca. Heliusviridae was verified in four previously published datasets All attempts at replication were successful
Randomization	Samples were allocated into groups based on clinical measurements of metabolic syndrome-related clinical parameters. Randomization was not relevant to this study, as no treatments were given to the participants.
Blinding	Blinding was irrelevant to this study, as no treatment was given to the participants. Furthermore, samples were selected from a large cohort based on knowledge of clinical criteria (i.e., whether participants had metabolic syndrome or not, which medicines they used, and whether they had supplied fecal material for analysis). This made blinding logically impossible.

Reporting for specific materials, systems and methods

We require information from authors about some types of materials, experimental systems and methods used in many studies. Here, indicate whether each material, system or method listed is relevant to your study. If you are not sure if a list item applies to your research, read the appropriate section before selecting a response.

Materials & experimental systems

n/a	Involved in the study	
☒	☐	Antibodies
☒	☐	Eukaryotic cell lines
☒	☐	Palaeontology and archaeology
☒	☐	Animals and other organisms
☐	☒	Human research participants
☒	☐	Clinical data
☒	☐	Dual use research of concern

Methods

n/a	Involved in the study	
☒	☐	ChIP-seq
☒	☐	Flow cytometry
☒	☐	MRI-based neuroimaging

Human research participants

Policy information about studies involving human research participants

Population characteristics	Participants were roughly evenly divided by ethnicity, with European Dutch comprising 49 controls and 49 MetS participants, and African Surinamese 50 controls and 49 MetS participants. The MetS group contained 55 women and had a median age of 58 (mean 56.8±8.09), and the controls 71 and had a median age of 50 (mean 49.1±12). Of the 196 participants, 26 used metformin, of whom 2 were controls who did not concur to the MetS criteria.
Recruitment	The Healthy Life in an Urban Setting (HELIUS) cohort includes some 25,000 ethnically diverse participants from Amsterdam, the Netherlands. The cohort details were published previously: Snijder, M. B. et al. Cohort profile: The Healthy Life in an Urban Setting (HELIUS) study in Amsterdam, the Netherlands. BMJ Open 7, 1–11 (2017). Inclusion into the current study was based on availablilty of sample material for analysis. Due to the increasing chance at developing MetS with increasing age, the average age, the MetS group was notably older than the controls. Additionally, the MetS group contained more men than the control group. Both of these biases were controlled for in statistical analyses across the study. This is further detailed in the methods and mentioned in the figure legends.
Ethics oversight	The study was approved by the HELIUS study board. The HELIUS cohort conformed to all relevant ethical considerations. It complied with the Declaration of Helsinki (6th, 7th revisions), and was approved by the Amsterdam University Medical Centers Medical Ethics Committee. All participants provided written informed consent

Note that full information on the approval of the study protocol must also be provided in the manuscript.
